# Vascular uptake on ^18^F-sodium fluoride positron emission tomography: precursor of vascular calcification?

**DOI:** 10.1007/s12350-020-02031-5

**Published:** 2020-01-23

**Authors:** Annemarie M. den Harder, Jelmer M. Wolterink, Jonas W. Bartstra, Wilko Spiering, Sabine R. Zwakenberg, Joline W. Beulens, Riemer H. J. A. Slart, Gert Luurtsema, Willem P. Mali, Pim A. de Jong

**Affiliations:** 1 Department of Radiology, Utrecht University Medical Center, P.O. Box 85500, E01.132, 3508 GA Utrecht, The Netherlands; 2 Image Sciences Institute, University Medical Center Utrecht, Utrecht University, Utrecht, The Netherlands; 3 Department of Vascular Medicine, University Medical Center Utrecht, Utrecht University, Utrecht, The Netherlands; 4 Julius Centre for Health Sciences and Primary Care, University Medical Center Utrecht, Utrecht University, Utrecht, The Netherlands; 5 Department of Epidemiology and Biostatistics, Amsterdam University Medical Center, Amsterdam, The Netherlands; 6 Medical Imaging Center, Department of Nuclear Medicine and Molecular Imaging, University Medical Center Groningen, University of Groningen, Groningen, The Netherlands

**Keywords:** Calcification, positron emission tomography, computed tomography, atherosclerosis, medial artery calcification

## Abstract

**Background:**

Microcalcifications cannot be identified with the present resolution of CT; however, ^18^F-sodium fluoride (^18^F-NaF) positron emission tomography (PET) imaging has been proposed for non-invasive identification of microcalcification. The primary objective of this study was to assess whether ^18^F-NaF activity can assess the presence and predict the progression of CT detectable vascular calcification.

**Methods and Results:**

The data of two longitudinal studies in which patients received a ^18^F-NaF PET-CT at baseline and after 6 months or 1-year follow-up were used. The target to background ratio (TBR) was measured on PET at baseline and CT calcification was quantified in the femoral arteries at baseline and follow-up. 128 patients were included. A higher TBR at baseline was associated with higher calcification mass at baseline and calcification progression (*β* = 1.006 [1.005-1.007] and *β* = 1.002 [1.002-1.003] in the studies with 6 months and 1-year follow-up, respectively). In areas without calcification at baseline and where calcification developed at follow-up, the TBR was .11–.13 (*P* < .001) higher compared to areas where no calcification developed.

**Conclusion:**

The activity of ^18^F-NaF is related to the amount of calcification and calcification progression. In areas where calcification formation occurred, the TBR was slightly but significantly higher.

**Electronic supplementary material:**

The online version of this article (10.1007/s12350-020-02031-5) contains supplementary material, which is available to authorized users.

## Introduction

Atherosclerosis is a major cause of disability and death worldwide.^1^ The amount of arterial calcification is a surrogate imaging marker of the burden of atherosclerotic disease. In an early stage, calcification in the form of microcalcifications can be histologically observed. Microcalcifications are associated with inflammation and unstable high-risk plaques. In a later stage, extensive macrocalcifications develop which are thought to stabilize the plaque.^2^,^3^ Computed tomography (CT)-based quantification of calcification is commonly performed for the coronary arteries, where it is an established predictor of cardiovascular disease.^4^ However, the present resolution of CT allows identification of macrocalcifications, but not of microcalcifications. Therefore, ^18^F-sodium fluoride (^18^F-NaF) positron emission tomography (PET) imaging has been proposed for non-invasive identification of areas with microcalcification, to bridge the gap between what can be measured with histology and with CT. However, the availability of data on ^18^F-NaF PET is limited and validation studies are challenging.

Higher ^18^F-NaF uptake is associated with cardiovascular risk factors.^5^–^7^ In bone imaging, ^18^F-NaF binds to the surface of hydroxyapatite crystals. Subsequently, the hydroxyl ion is exchanged with the ^18^fluoride ion on the surface of hydroxyapatite to form fluorapatite. Vascular ^18^F-NaF uptake might therefore be a measure of the calcification surface area and able to visualize smaller calcifications than CT as these microcalcifications have a large surface area relative to the amount of calcium.^8^ It has also been proposed that ^18^F-NaF specifically binds areas of microcalcification, and therefore can identify areas of active calcification.^9^ We performed a longitudinal study to assess whether ^18^F-NaF activity is associated with incident and progressive vascular calcification on CT in the femoral arteries, which is an arterial bed where motion artifacts do not play a major role.

## Methods

For this study, data from two longitudinal medication studies (TEMP and VITACAL trial, trialregister.nl NL4956 and NL5147) in which patients underwent an ^18^F-NaF PET-CT at baseline and after 1 year (TEMP) or 6 months (VITACAL) follow-up were used. In both studies, TBR on PET and calcium mass on CT were measured at baseline and follow-up.

### TEMP

The Treatment of Ectopic Mineralization in Pseudoxanthoma Elasticum Trial (TEMP) was a single center, randomized, double-blind, placebo-controlled trial conducted in the University Medical Center Utrecht in the Netherlands. Patients with a clinical diagnosis of Pseudoxanthoma Elasticum (PXE),^10^ aged ≥ 18 years and with evidence of arterial calcification on a CT of the lower limbs were included. In total, 74 patients were randomized between 1 year treatment with a bisphosphonate (etidronate) and placebo. At baseline and after 1-year treatment, a full body ^18^F-NaF PET-CT was performed. The study was previously described in detail.^11^

### VITACAL

The VItamin K and vascular calcification (VITACAL) study was a single center, randomized, double-blind, placebo-controlled trial conducted in the University Medical Center Utrecht in the Netherlands. Patients diagnosed with diabetes mellitus type 2, aged ≥ 40 with presence of arterial disease (Ankle Brachial Index < .9 and/or diagnosed with arterial disease by physician) were included. Patients were randomized between 6 months treatment with 360 microgram vitamin K (menaquinone-7) daily and placebo. At baseline and after 6 months treatment a full body ^18^F-NaF PET-CT was performed. The study was previously described in detail.^12^

### Data Acquisition

The CT and PET acquisition parameters were described previously.^11^,^12^ In all patients, calcifications in both femoral arteries were quantified on CT using dedicated software (iX Viewer, Image Sciences Institute, University Medical Center Utrecht, Utrecht, The Netherlands). A threshold of 130 Hounsfield Units was applied, and the observer manually selected arterial calcifications above this threshold with a single mouse click. Subsequently, the calcification mass score was calculated. The mass equivalent score was computed as the product of the mean attenuation in a lesion and the volume of that lesion. Scoring was started a centimeter below the bifurcation of the communal femoral artery up till the femur condyles.

On PET the standardized uptake value (SUV_max_) was determined by manually drawing a circular region of interest (ROI) around the femoral artery on every other slice (slice thickness 5 mm, increment 4 mm). The mean SUV_background_ was determined by using the average of three ROIs drawn in the superior vena cava at the level of the aortic arch on consecutive slices. The target to background ratio (TBR) was calculated by dividing the SUV_max_ by the SUV_background_. The observer was blinded for treatment status and clinical information.

**Figure 1:**
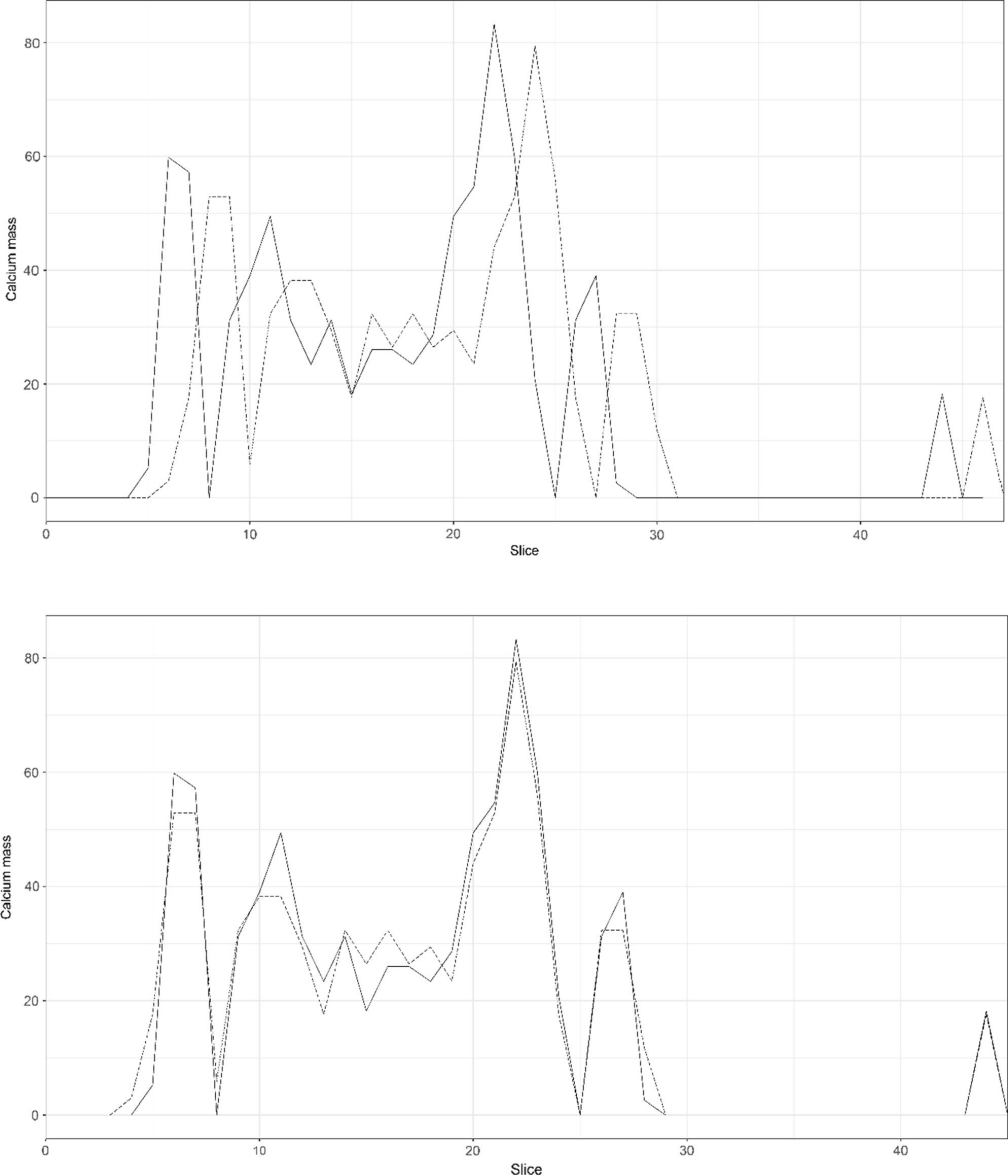
Example of alignment. Example of alignment of baseline and follow-up acquisition using the amount of calcification. The continuous line shows calcification at baseline, and the dashed line represents the follow-up acquisition. In the upper figure (before translation), the two lines do not overlap. By moving the dashed follow-up line two slices to the left, a better fit is achieved

To determine calcification progression in individual image slices, slices at baseline and follow-up were aligned. For each lower limb in each acquisition, calcium mass score profiles along the femoral artery were determined. An example is provided in Figure 1. Because calcification progression in 6 months or 1 year is typically limited, these profiles should correlate for baseline and follow-up. In patients where there was poor alignment, a translation of the follow-up CT scan was applied to create a better fit. The translation was performed by one observer (AH), after which profiles with and without translation were anonymized and scored by two observers (JB, PJ). Each observer chose which plot fitted better and provided a score: (1) perfect overlap, (2) moderate overlap, and (3) poor overlap. All plots in which only one of the observers scored 3 or when the observers had a different plot preference were discussed in a consensus meeting with all three observers. In the consensus meeting, it was decided whether to include the data or not. If both observers scored 3, data were excluded from further analysis. Plots of all patients are provided in Online Appendix 1 (TEMP) and Online Appendix 2 (VITACAL). The result of the matching was one final dataset that included per patient, per lower limb, per slice the calcium mass at baseline and follow-up, the TBR at baseline, and the delta calcium mass. The delta calcium mass was calculated by subtracting the calcium mass at baseline from the calcium mass at follow-up.

### Statistical Analysis

Statistical analysis was performed with RStudio version 1.1.414 (RStudio Team, Boston, USA). Data are provided as mean ± standard deviation in case of a normal distribution and as median [interquartiles] if non-normally distributed. Categorical variables are provided as *N* (percentage).

A multilevel linear model was used since data were clustered per patient and per side. The natural log transformation of the TBR (as measured at baseline) was used as dependent variable, adjusted for age, gender, BMI and renal function (creatinine level). Subsequently, stratified analyses per treatment group were performed. Presented are the results after back transformation.

Subgroup analysis was performed in slices without calcium at baseline. Slices in which calcium progression occurred were compared to slices without calcium at follow-up.

Finally, we investigated whether high TBR values were associated with calcium progression. This was only investigated in the placebo group, since treatment might interfere with calcium progression. From all slices without calcium at baseline, the slice with the highest TBR at baseline was selected in each patient in each lower limb. This was defined as the ‘hottest slice’. If multiple slices had the same highest TBR, they were all marked as the hottest slice. The calcium mass at follow-up on these hottest slices was compared to the remaining slices using the Mann–Whitney-U test.

## Results

### TEMP

**Figure 2:**
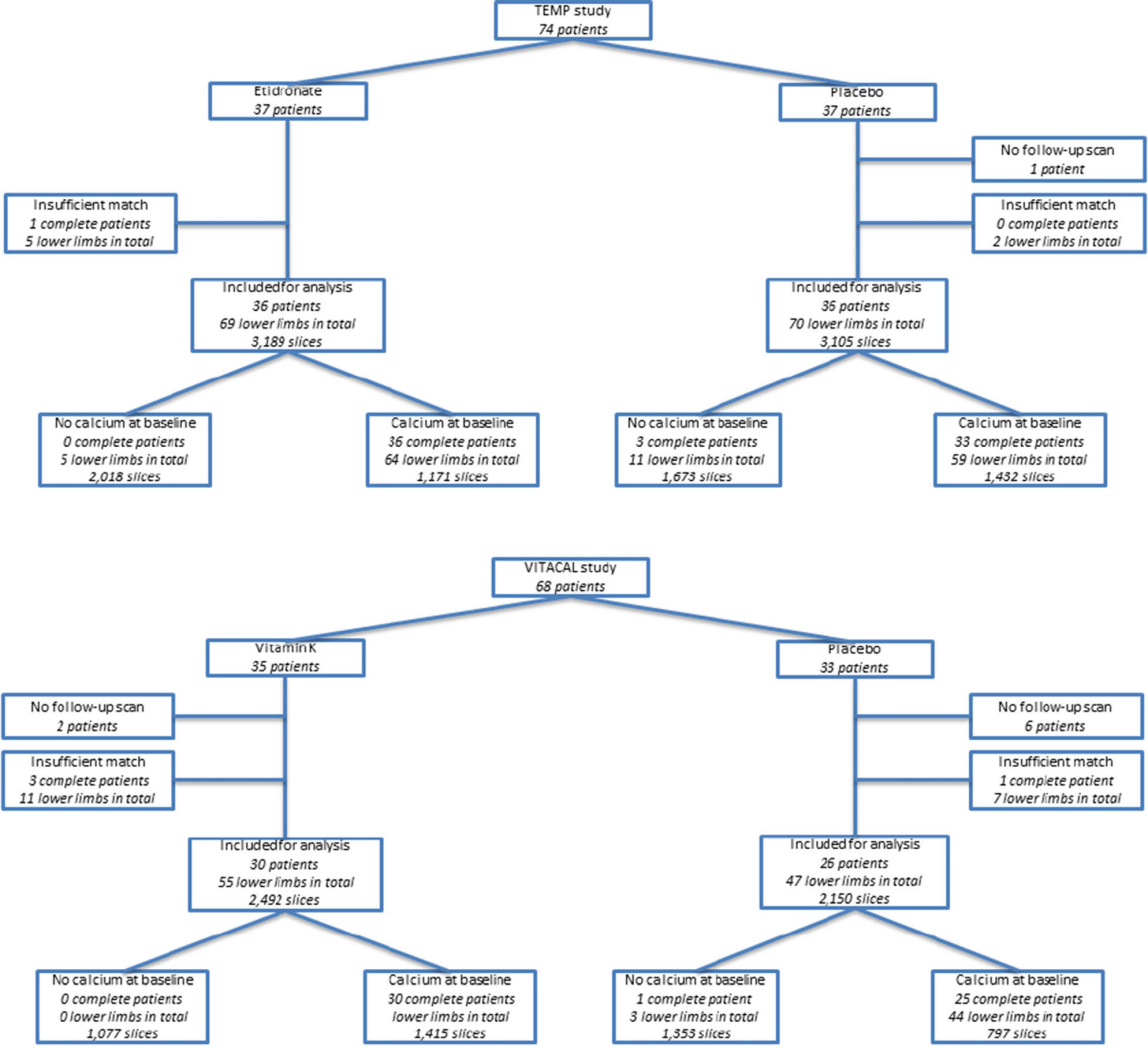
Flowchart of the TEMP and VITACAL study

**Figure 3:**
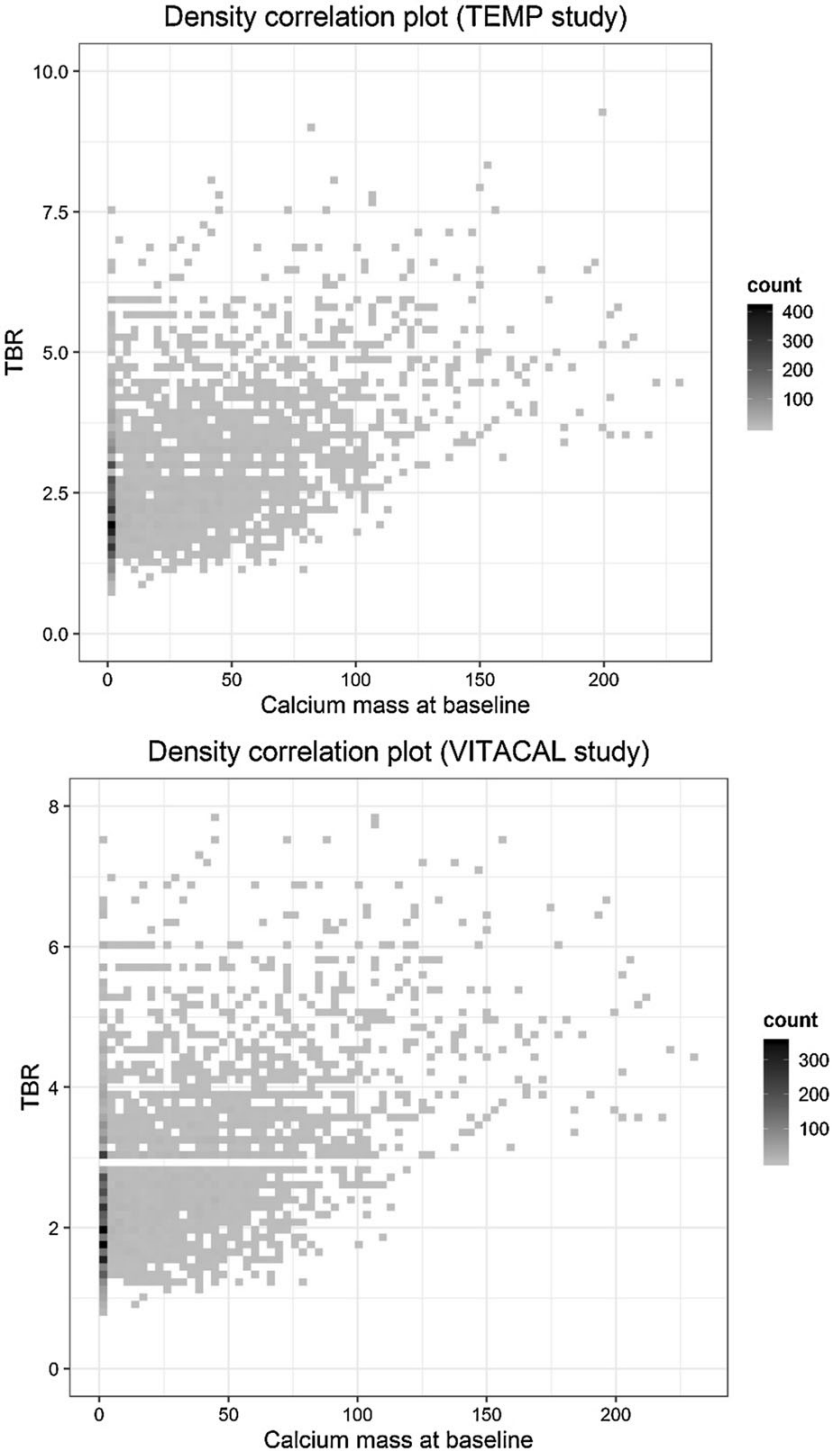
Correlation between calcium mass and TBR. Density plot of the correlation between the calcium mass at baseline and the TBR at baseline for the TEMP and VITACAL study

In total 74 patients were included. One patient did not receive the follow-up scan and was therefore excluded. After the consensus meeting seven lower limbs in six different patients (in one patient both limbs, in five patients one limb) were excluded because the baseline and follow-up data could not be matched sufficiently. Ultimately, 72 patients (139 lower limbs) were included with 6,294 slices in total. Forty-one percent (2,603/6,294) of the CT slices showed calcium at baseline. Of those slices, 1,108 slices (43%) showed calcium progression at follow-up. The baseline TBR of those slices was 3.2 compared to 3.1 in slices without calcium progression (*P* = .67). Baseline characteristics are provided in Table 1. A flowchart is provided in Figure 2. Figure 3 shows the correlation between the TBR and the calcium mass.

**Table 1:** Baseline characteristics

TEMP study		
Per patient (*N* = 72)	Etidronate (*N* = 36)	Placebo (*N* = 36)
Age (years)	56.7 ± 8.6	57.3 ± 8.1
Gender (male)	19 (53%)	19 (53%)
BMI (kg · m^2^)	26.6 ± 4.6	25.7 ± 3.3
Systolic blood pressure (mmHg)	143 ± 20	137 ± 18
Diabetes Mellitus type 2	3 (8%)	1 (3%)
Triglycerides (mmol · L^−1^)	1.4 ± .8	1.5 ± 1.4
Total cholesterol (mmol · L^−1^)	5.2 ± 1.3	5.4 ± 1.4
HDL (mmol · L^−1^)	1.6 ± .5	1.5 ± .3
LDL (mmol · L^−1^)	3.0 ± 1.1	3.2 ± 1.1
Creatinine level (µmol · L^−1^)	71 ± 15	70 ± 13
Number of slices	3,189 (51%)	3,105 (49%)

TEMP study		
Per slice (*N* = 6,294)	Etidronate (*N* = 3,189)	Placebo (*N* = 3,105)
TBR at baseline	2.7 ± 1.2	2.6 ± 1.1
Calcium mass at baseline	.0 [.0–23.8]	.0 [.0–31.7]
Calcium mass at follow-up	.0 [.0–22.9]	.0 [.0–32.5]
Delta calcium mass (all slices)	− .9 ± 11.4	.4 ± 11.1
Delta calcium mass (slices with calcium at baseline)	− 2.9 ± 18.2	.2 ± 15.4
Delta calcium mass (slices with no calcium at baseline but with calcium at follow-up)	12.4 ± 13.4	13.2 ± 11.5

VITACAL study		
Per patient (*N* = 55)	Vitamin K (*N* = 30)	Placebo (*N* = 26)
Age (years)	68.2 ± 7.5	70.8 ± 7.8
Gender (male)	25 (83%)	19 (73%)
BMI (kg · m^−2^)	30.9 ± 5.9	31.2 ± 5.2
Smoking status		
Current	4 (13%)	2 (8%)
Stopped	22 (73%)	14 (54%)
Never	4 (13%)	10 (38%)
Systolic blood pressure (mmHg)	141 ± 21	142 ± 17
Diabetes mellitus type 2	30 (100%)	26 (100%)
HbA1c (mmol · mol^−1^)	58 ± 16	61 ± 18
Coronary artery disease	19 (63%)	15 (58%)
Cerebrovascular disease (CVA/TIA)	9 (30%)	8 (31%)
Abdominal aortic aneurysm	2 (7%)	4 (15%)
Peripheral artery disease	6 (20%)	8 (31%)
Triglycerides (mmol · L^−1^)	3.7 ± 4.9	2.4 ± 1.6
Total cholesterol (mmol · L^−1^)	4.6 ± 1.4	4.4 ± 1.2
HDL (mmol · L^−1^)	1.1 ± .3	1.2 ± .3
LDL (mmol · L^−1^)	2.2 ± .9	2.2 ± 1.0
Creatinine level (µmol · L^−1^)	80 ± 22	90 ± 26
Number of slices	2,492	2,150

VITACAL study		
Per slice (*N* = 4,548)	Vitamin K (*N* = 2,492)	Placebo (*N* = 2,056)
TBR at baseline	2.3 ± .9	2.1 ± .9
Calcium mass at baseline	.0 [.0–12.3]	.0 [.0–6.2]
Calcium mass at follow-up	.0 [.0–12.9]	.0 [.0–6.6]
Delta calcium mass (all slices)	.7 ± 5.6	.5 ± 5.9
Delta calcium mass (slices with calcium at baseline)	1.3 ± 7.0	1.4 ± 9.7
Delta calcium mass (slices with no calcium at baseline but with calcium at follow-up)	3.6 ± 3.5	3.5 ± 5.2

Baseline characteristics of the TEMP and VITACAL study

*BMI*, Body Mass Index; *TBR*, Target to Background Ratio

Results of univariate analysis are provided in Online Appendix 3. Multivariate analysis (Table 2) showed that a higher TBR was positively associated with both an increase in the delta calcium mass (*β* = 1.002 [1.002-1.003], *P* < .001) and the amount of calcium at baseline (*β* = 1.006 [1.005-1.006], *P* < .001). Analysis stratified per group (placebo or etidronate) showed the same direction and effect sizes (Online Appendix 4).

**Table 2:** Multilevel linear regression model

Variable	Regression coefficient *β* [95% CI]	*P* value
TEMP study		
Age (years)	1.002 [.994–1.010]	.684
Gender (male)	1.028 [.878–1.204]	.738
BMI (kg · m^−2^)	.987 [.970–1.004]	.141
Creatinine level (µmol · L^−1^)	.997 [.992–1.003]	.369
Baseline calcium mass	1.006 [1.005–1.006]	< .001
Delta calcium mass	1.002 [1.002–1.003]	< .001
VITACAL study		
Age (years)	1.002 [.994–1.010]	.443
Gender (male)	1.009 [.857–1.188]	.917
BMI (kg · m^−2^)	1.011 [.999–1.023]	.093
Creatinine level (µmol · L^−1^)	.999 [.996–1.001]	.309
Baseline calcium mass	1.014 [1.013–1.014]	< .001
Delta calcium mass	1.006 [1.005–1.007]	< .001

Results of the multilevel linear regression model with TBR at baseline as dependent variable

**Figure 4:**
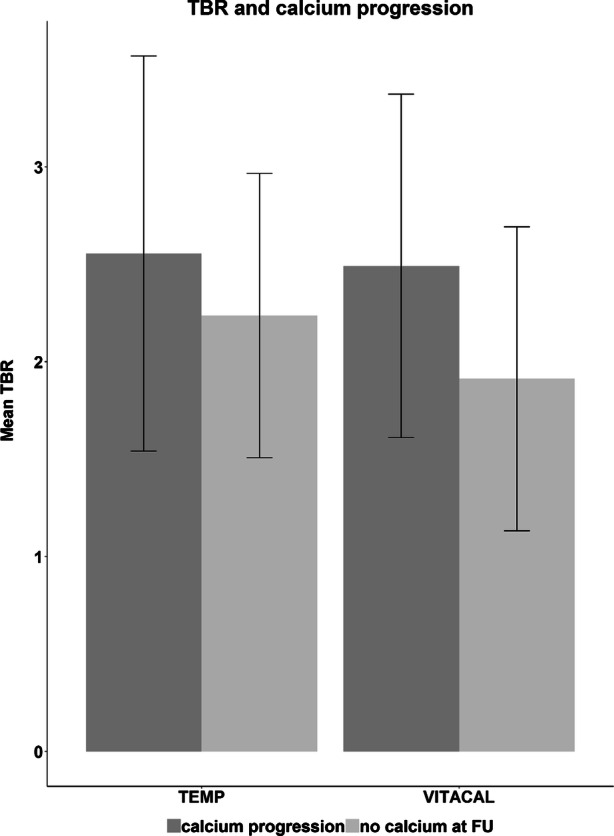
^18^F-NaF activity and calcium progression. Relationship between baseline TBR and calcium progression in the placebo groups in slices without calcium at baseline. Slices in which calcium progression is seen at follow-up have a higher TBR at baseline in both studies (*P* < .001 in both studies). *FU*, Follow-Up; *TBR*, Target to Background Ratio

There were 3,691 slices without calcium at baseline. In 8% (286 slices) of those slices, calcium developed at follow-up. Figure 4 shows that slices in which calcium developed at follow-up have a higher TBR at baseline. In multivariate analysis, slices without CT calcium at baseline in which calcium developed at follow-up showed a TBR at baseline which was .13 higher compared to slices in which no calcium formation occurred (*β* = 1.131 [1.105-1.159], *P* < .001).

**Figure 5:**
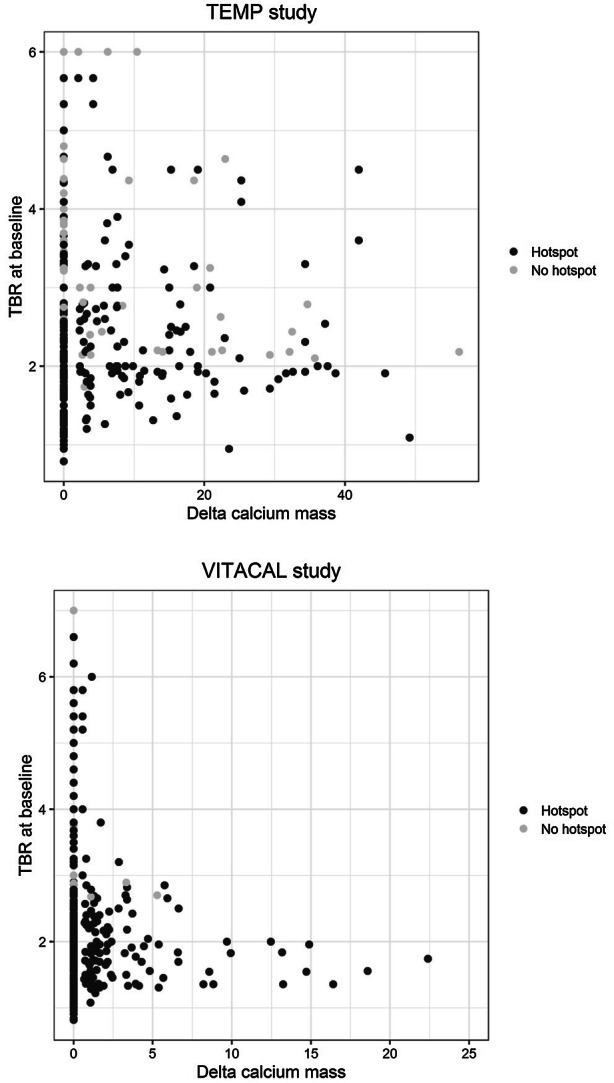
Calcification progression in hottest slices. The figure shows the relationship between the TBR and the calcification progression (delta calcium mass) for hotspots compared to the other slices

The hottest slice analysis in slices without calcium at baseline in the placebo group showed that the progression of the calcium mass at follow-up was significantly higher for the hottest slice (slice with the highest TBR at baseline, *P* < .001). The hottest slice (148 slices) showed a mean calcium mass score at follow-up of 3.204 (SD 8.684) vs 1.064 (SD 4.728) in the 1,525 other slices (*P* < .001). In 20% (30/148) of the hottest slices and in 8% (129/1,525) of the remaining slices calcium developed at follow-up. Figure 5 shows the mean calcium mass for the hottest slice compared to the other slices.

### VITACAL

In total 68 patients were included in the VITACAL study. Eight patients did not receive the follow-up scan and were therefore excluded. After the consensus meeting 18 lower limbs in 14 different patients (in four patient both limbs, in ten patients one limb) were excluded. Therefore 56 patients (101 lower limbs) were included with 4,642 slices in total. Forty-eight percent (2,212/4,642) of the slices showed calcium at baseline. Of those slices, 1,152 slices (25%) showed calcium progression at follow-up. The baseline TBR of those slices was 2.7 compared to 2.6 in slices without calcium progression (*P* = .01). Baseline characteristics are provided in Table 1. Figure 3 shows the correlation between the TBR and the calcium mass.

Multivariate analysis (Table 2) showed that a higher TBR was associated with both an increase in the delta calcium mass (*β* = 1.006 [1.005-1.007], *P* < .001) and the amount of calcium at baseline (*β* = 1.014 [1.013-1.014], *P* < .001). Analysis stratified per group (placebo or vitamin K) showed the same direction and effect sizes (supplement 4).

There were 2,430 slices without calcium at baseline. In 13% (322 slices) of those slices, calcium developed at follow-up. Figure 4 shows that slices in which calcium develop at follow-up have a higher TBR at baseline. In multivariate analysis, slices without CT calcium at baseline in which calcium developed at follow-up show a TBR at baseline which is .11 higher compared to slices in which no calcium formation occurred (*β* = 1.113 [1.084-1.142], *P* < .001).

The hottest slice analysis in slices without calcium at baseline in the placebo group showed that the progression of the calcium mass at follow-up was not significantly different for the hottest slice (slice with the highest TBR at baseline). The hottest slice (32 slices) showed a mean calcium mass score at follow-up of .304 (SD 1.098) vs .345 (SD 1.097) in the 1,321 other slices (*P* = .960). In 3/32 (9%) of the hottest slices and in 130/1,321 (10%) of the remaining slices calcium developed at follow-up. Figure 5 shows the mean calcium mass for the hottest slice compared to the other slices.

## Discussion

In this longitudinal study, ^18^F-NaF PET uptake showed a weak positive relation with the amount of CT calcification, CT calcification progression and newly developed CT calcifications. Although a higher TBR in areas without calcium at baseline was related to the occurrence of calcification at follow-up, the difference was small (.11-.13) and therefore the clinical relevance might be limited.

Longitudinal studies which assess the relationship between ^18^F-NaF activity and disease progression are limited and involve small patient groups. Jenkins et al.^13^ assessed aortic valve calcification in 99 patients. ^18^F-NaF activity was strongly correlated to both the amount of calcium at baseline and calcium progression. However, ^18^F-NaF activity was not able to independently predict clinical outcomes (cardiovascular death and aortic valve replacement). Ishiwata et al.^14^ retrospectively evaluated 34 patients who underwent a PET-CT because of malignancy or orthopedic diseases. Follow-up was after 1 year (for calcified plaques) or more than 2 years (for ^18^F-NaF active sites) with a large range in follow-up. The aorta and the common iliac artery were studied. There was no correlation between ^18^F-NaF activity and baseline calcium; however, the difference in calcium volume and Agatston calcium score were associated with a higher ^18^F-NaF activity. No correction for potential confounders was performed. De novo calcification occurred in 19/96 (20%) of the hotspots and was related to a longer follow-up interval of on average 5 years, but was not related to a higher TBR at baseline. In the current study de novo calcifications occurred in 20% (TEMP) and 9% (VITACAL) of the hotspots.

It would be very promising if a non-invasive biomarker could distinguish between microcalcifications in high-risk plaques and macrocalcifications in relatively stable plaques. Ex vivo research was performed by Irkle et al.^9^ in excised carotid endarterectomy specimens. Electron microscopy was compared with immunohistochemical analysis and ^18^F-NaF adsorption. Tissue fluoride was only found in the presence of calcification and microcalcifications showed greater levels of fluoride compared to macrocalcifications. ^18^F-NaF did only bind to the surface of the micro- and macrocalcifications. Recently, Creager et al.^15^ in vitro and ex vivo confirmed that ^18^F-NaF binds to the surface of both micro- and macrocalcifications. ^18^F-NaF is a marker of active calcification which is more likely to occur in the early stages of plaque formation, and not a specific marker of microcalcification. This explains the relationship between the amount of calcification and ^18^F-NaF activity, which was also reported in several earlier studies.^5^,^16^

Of special interest are areas without calcification, as measured on CT, but with increased ^18^F-NaF activity. These might represent areas with microcalcifications, not yet detectable by CT. In both studies we found that the hottest slice at baseline had a higher calcification mass at follow-up. In the literature, several different methods are used to define hotspots. Hotspots can be defined visually, using a fixed cut-off or using a study or patient specific cut-off as was performed in the current study. Since ^18^F-NaF activity is dependent on many variables such as age, BMI, renal function, injected dose and PET technology,^17^,^18^ it is difficult to define a fixed cut-off TBR.

This study has several limitations. First, both studies were clinical trials evaluating a bisphosphonate and vitamin K, respectively. Since the TBR was defined at baseline, it is not possible that this was influenced by treatment. However, the delta calcium can be influenced since the TEMP trial showed that bisphosphonate treatment reduced the amount of arterial calcification.^11^ Subgroup analysis in the placebo group, however, showed the same results. Second, because the TEMP and the VITACAL concerned very different patient populations and follow-up time the results were not pooled, although pooling of results would have increased the power of the study. Third, the inclusion criteria of both studies resulted in a study population with a high prevalence of femoral artery macrocalcifications, while ^18^F-NaF activity might allow identification of microcalcifications. Fourth, the coronary arteries, carotid bifurcation and aorta are vascular beds in which atherosclerosis is dominant, but in the femoral artery also medial arterial calcifications occur.^19^,^20^ In PXE patients, calcification of elastin fibers in the medial arterial wall occur which is different from atherosclerosis which is characterized by calcification of the intimal layer.^21^ Therefore, the results in PXE patients might not be generalizable to the atherosclerosis in general. In patients with diabetes, which was an inclusion criterion of the VITACAL study, medial artery calcifications of the lower limb are common.^22^ This may have influenced the associations between PET and CT in our study as the surface area of medial arterial calcification relative to atherosclerotic microcalcification is uncertain. However, the femoral artery is an arterial bed where motion artifacts do not play a major role. Furthermore, detection of iliofemoral plaques may identify earlier manifestations of atherosclerosis and can be detected in young individuals before the development of coronary calcifications.^23^ Fifth, the length of follow-up was relatively short, since progression of calcification is a slow process. Finally, we choose to use the calcium mass equivalent score for quantification of calcification in the femoral arteries, instead of the Agatston score which is typically used for calcium quantification in the coronary arteries. This is based on previous studies which show that the calcium mass allows for more robust quantification and shows better reproducibility than the Agatston score.^24^,^25^

## Conclusions

In conclusion this is the first longitudinal study showing that ^18^F-NaF activity in the femoral arteries is associated with the amount of CT calcification and CT calcification progression. Areas in which CT calcification occurred at follow-up, showed a slightly higher TBR at baseline, although the differences were small. This supports the concept that PET can detect calcifications below the resolution of CT to some extent, which may be of interest for studies in persons who do not have CT detectable calcifications.

## New Knowledge Gained

This study showed that ^18^F-NaF activity in the femoral arteries is related to the amount of calcification and calcification progression. To some extent, PET-CT can detect calcifications below the resolution of CT, and identify locations where calcifications are likely to develop on CT. This may be of interest for intervention studies in the early phases of the calcification process.

## Electronic supplementary material

Below is the link to the electronic supplementary material.

Online Appendix 1: Plots of relationship between TBR at baseline and the calcium mass at baseline and follow-up for the TEMP study (PDF 6962 kb)

Online Appendix 2: Plots of relationship between TBR at baseline and the calcium mass at baseline and follow-up for the VITACAL study (PDF 5181 kb)

Online Appendix 3: Results of univariate linear regression (DOCX 14 kb)

Online Appendix 4: Results of multilevel linear regression models stratified per treatment group for both studies (DOCX 20 kb)

Supplementary material 5 (M4A 5455 kb)

Supplementary material 6 (PPTX 284 kb)

## Abbreviations

^18^F-NaF: ^18^F-sodium fluoride
BMI: Body Mass Index
CT: Computed Tomography
PET: Positron Emission Tomography
PXE: Pseudoxanthoma Elasticum
ROI: Region Of Interest
SUV: Standardized Uptake Value
TBR: Target to Background Ratio
TEMP: Treatment of Ectopic Mineralization in Pseudoxanthoma Elasticum
