# Supplementary figures and images for: Vascular uptake on 18F-sodium fluoride positron emission tomography: precursor of vascular calcification?
Source: J Nucl Cardiol. 2020 Jan 23;28(5):2244–54. doi: 10.1007/s12350-020-02031-5 (PMC8648691; doi:10.1007/s12350-020-02031-5)

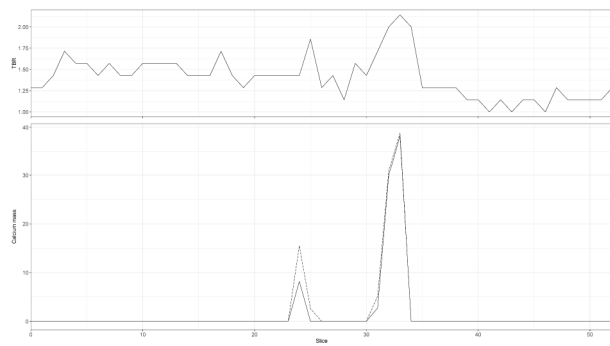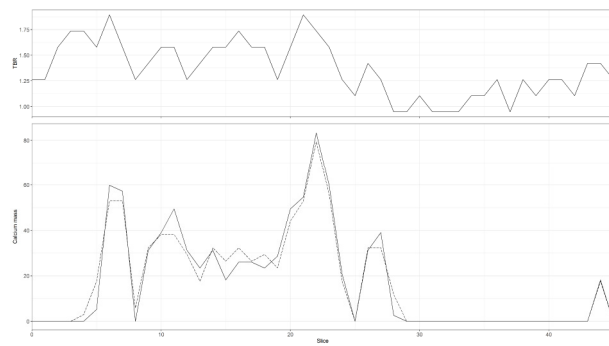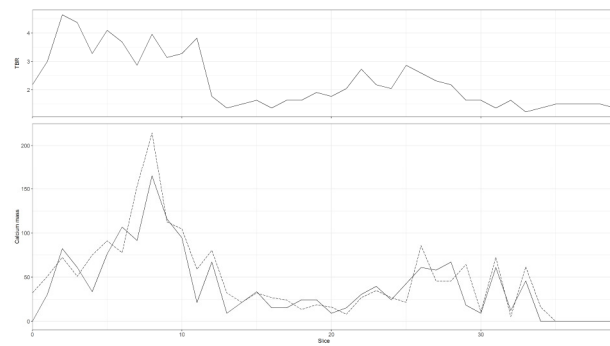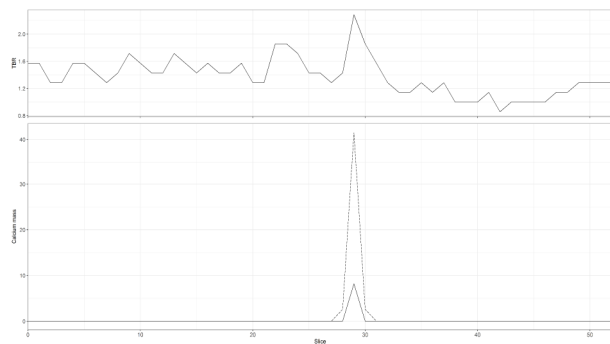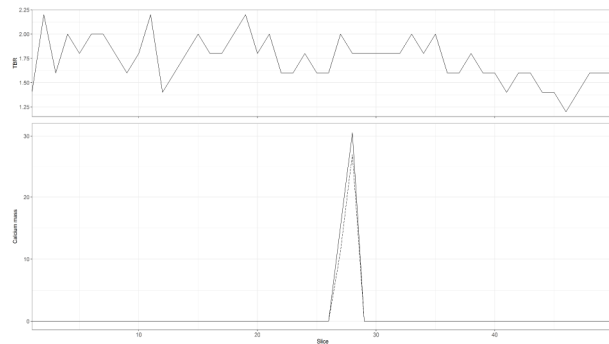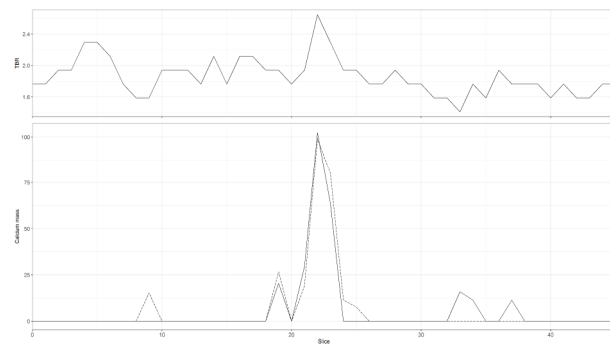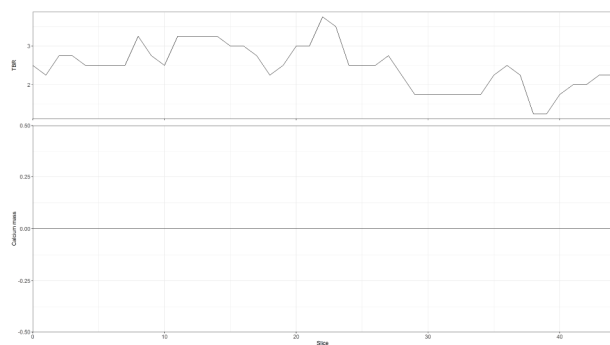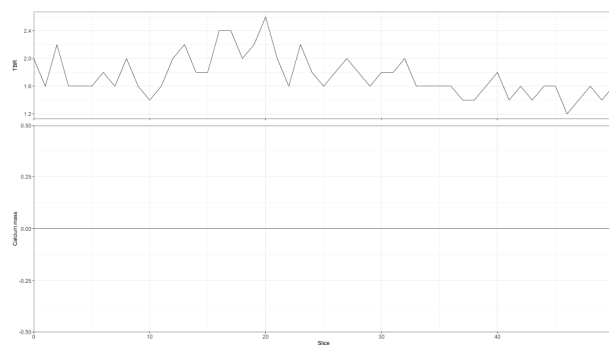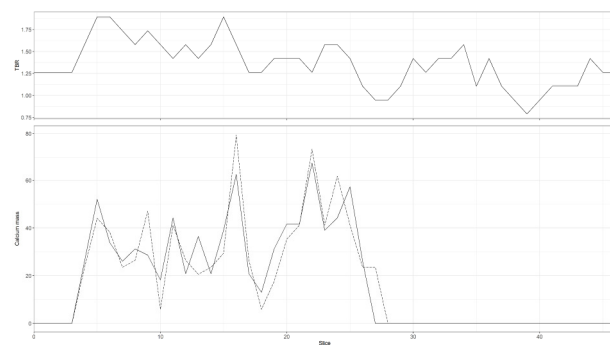

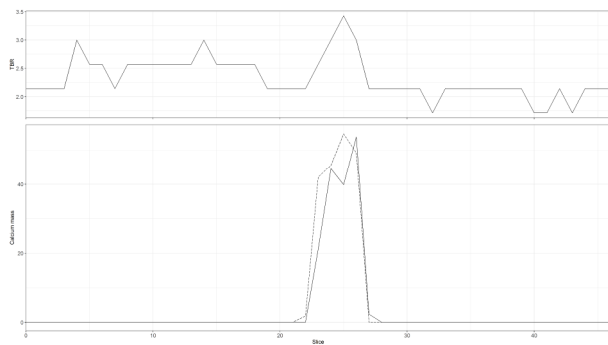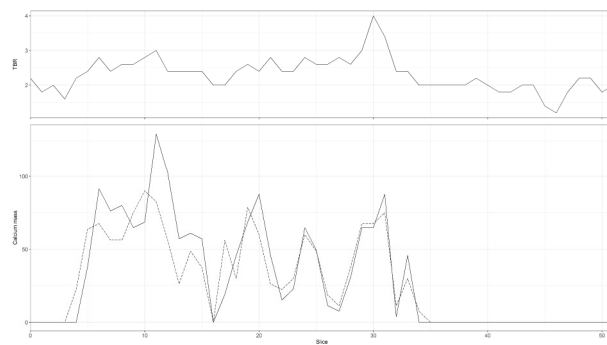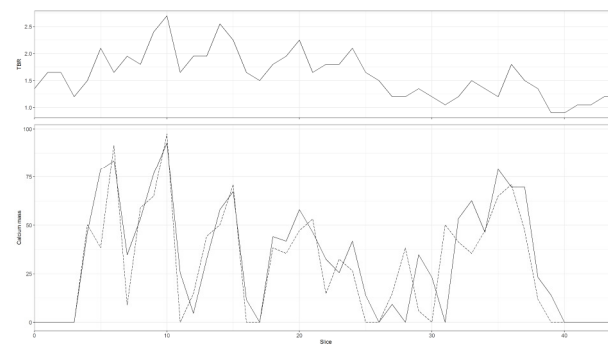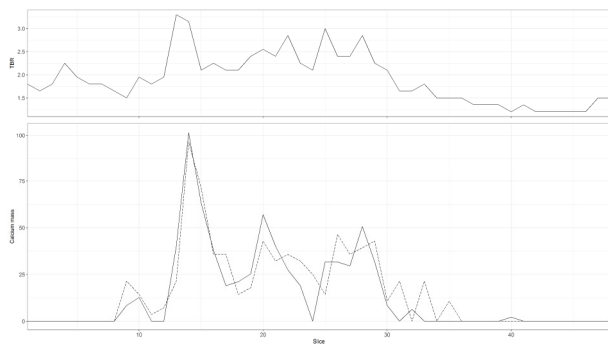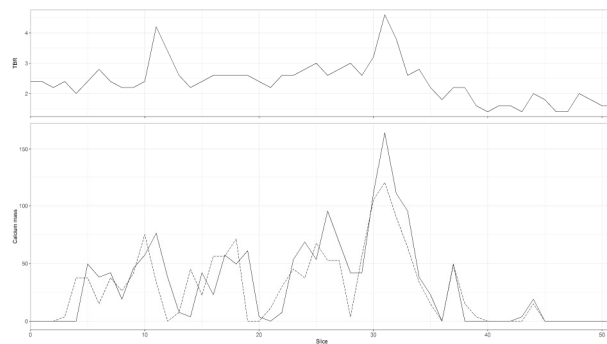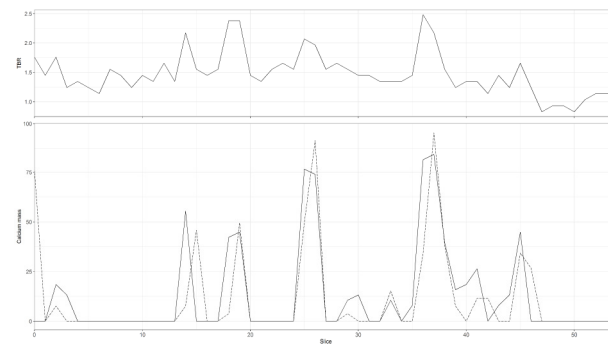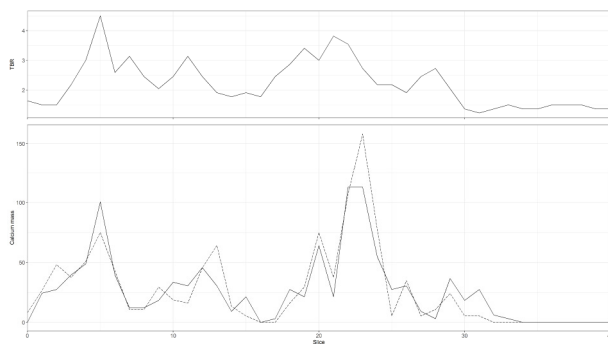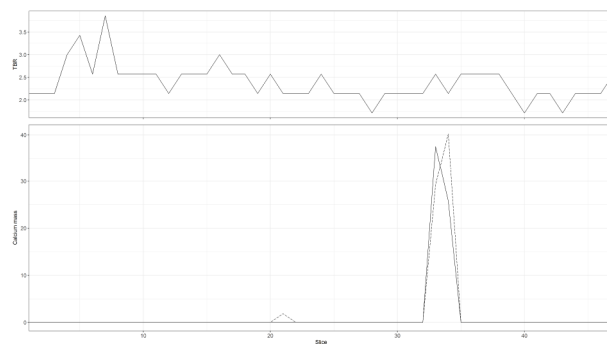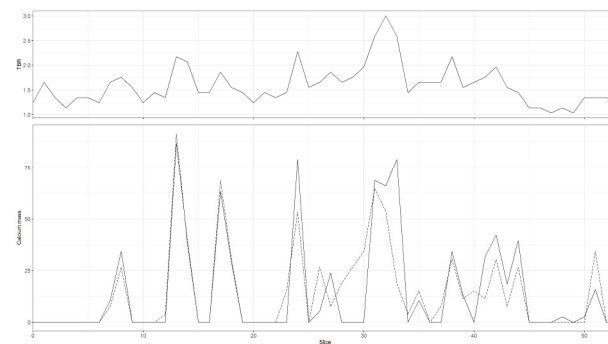

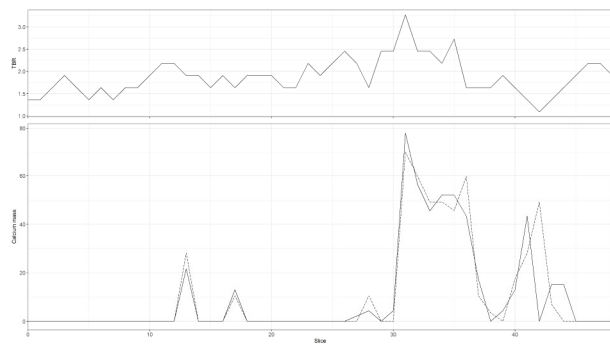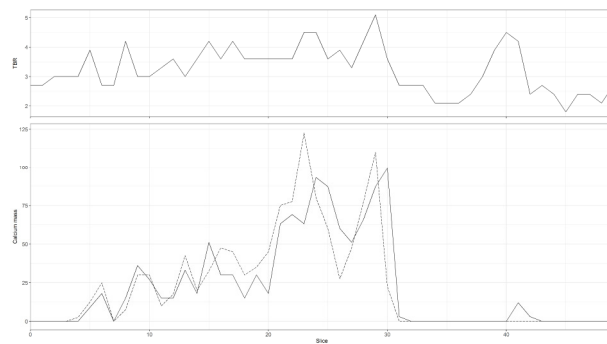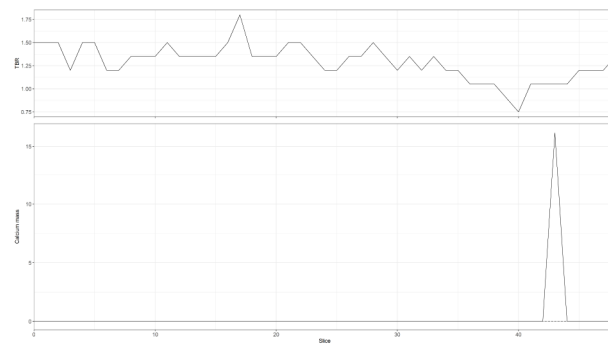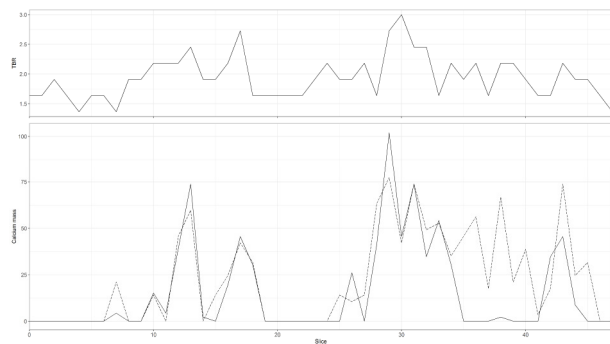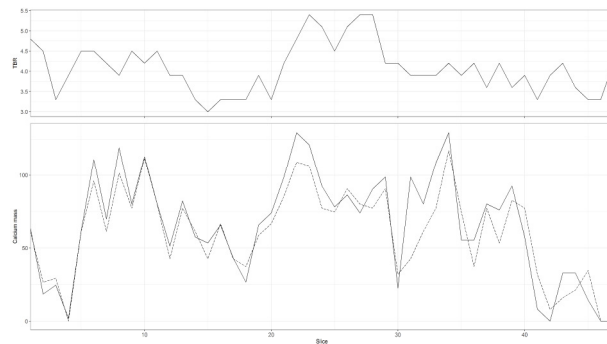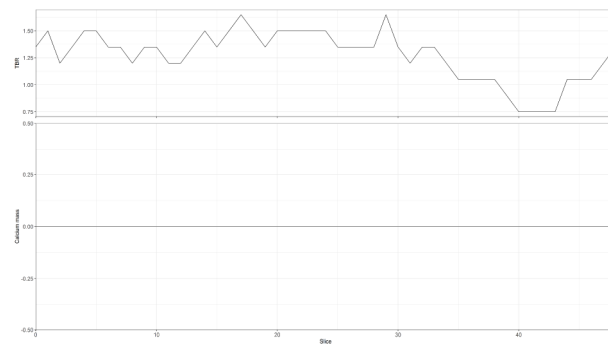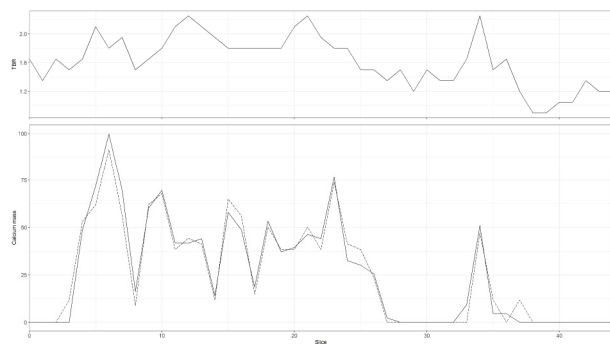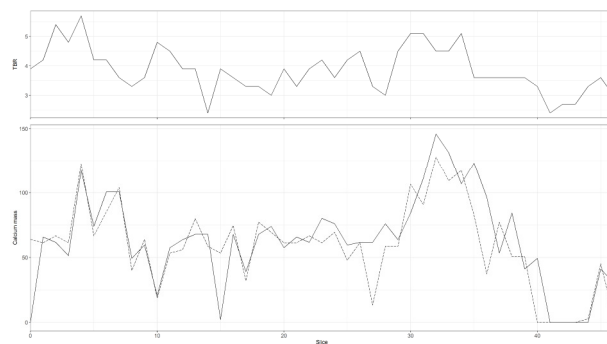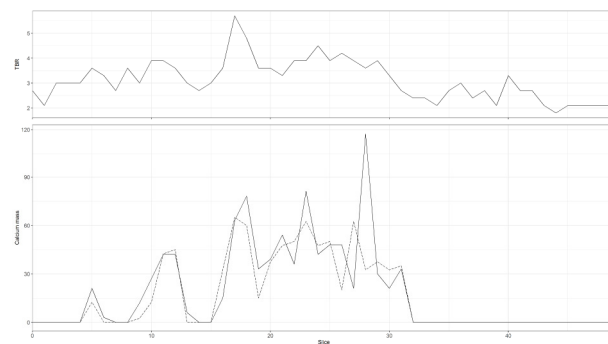

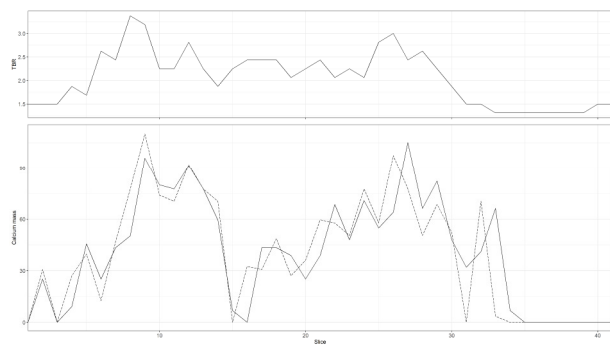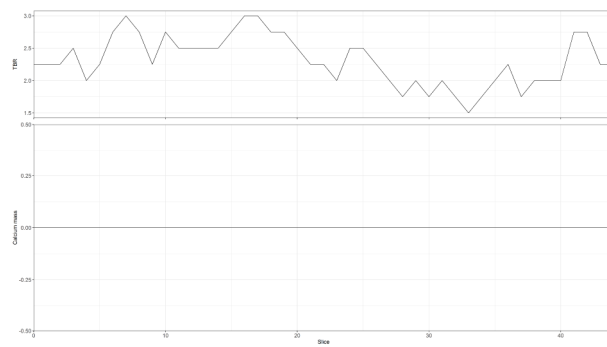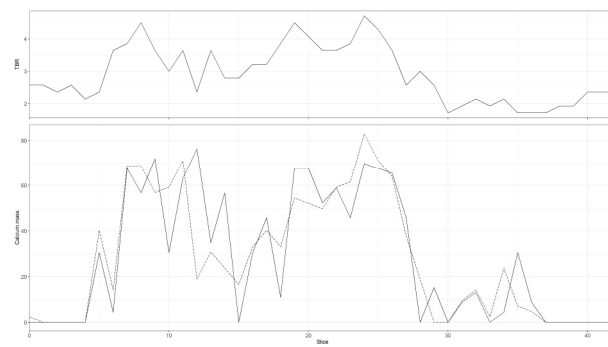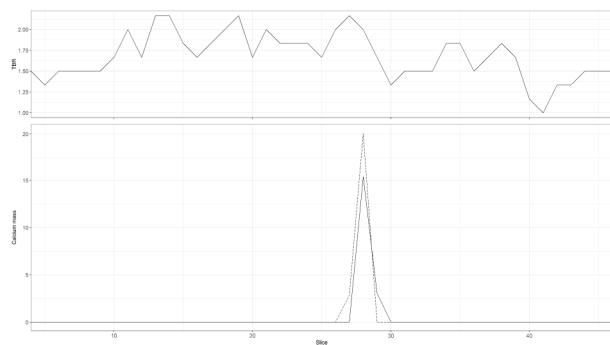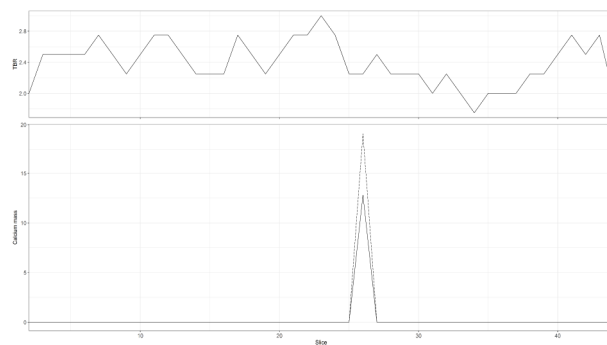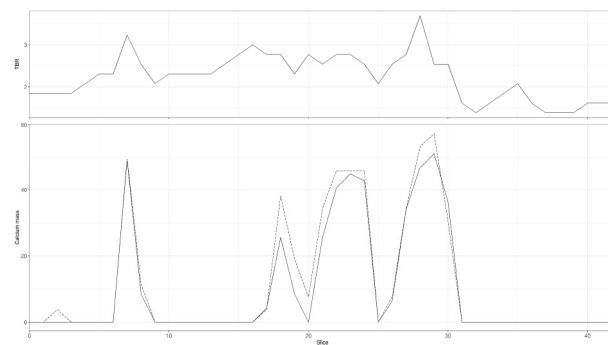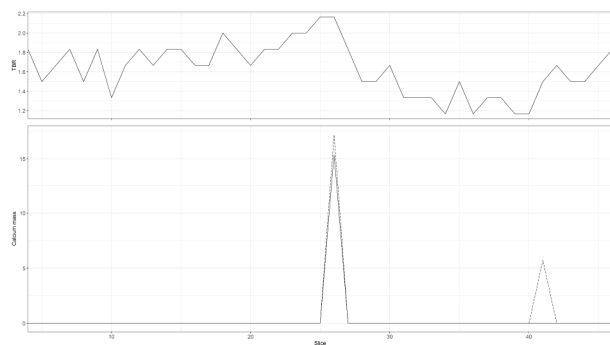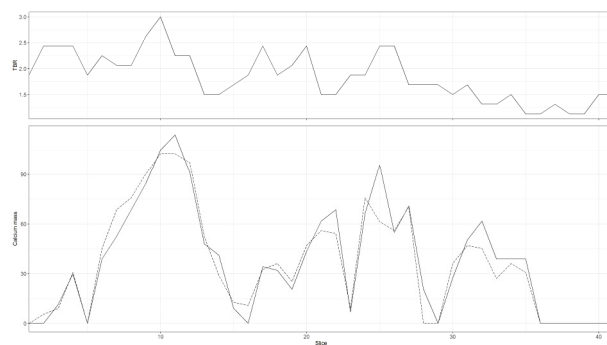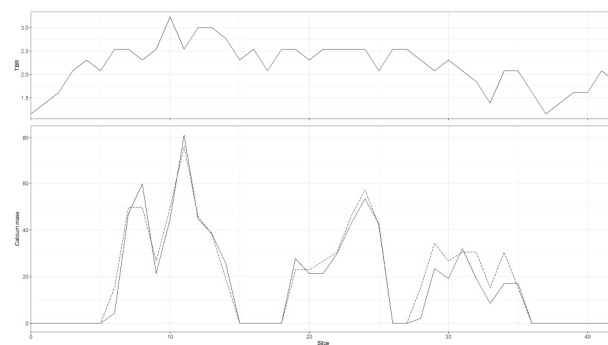

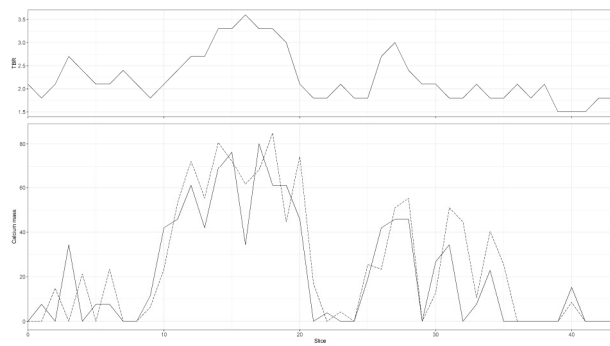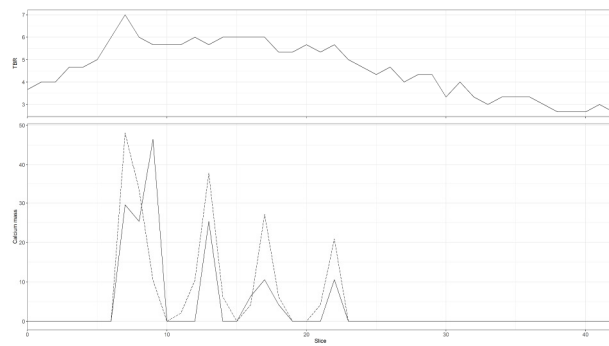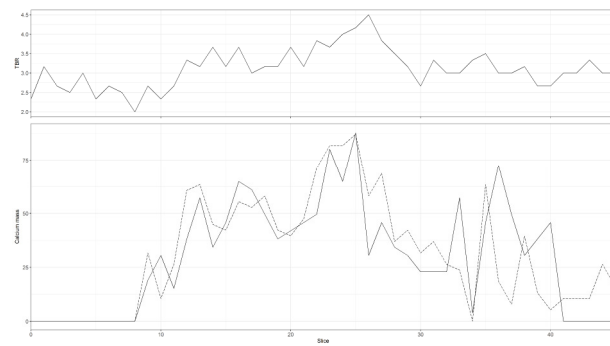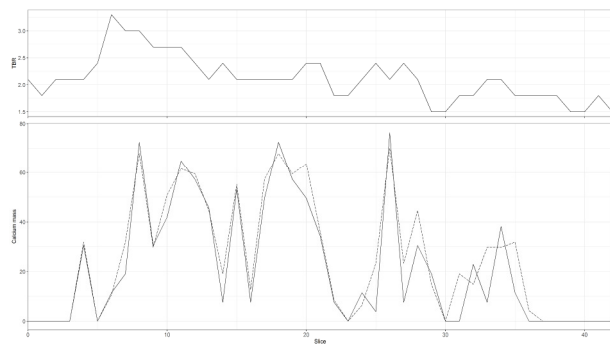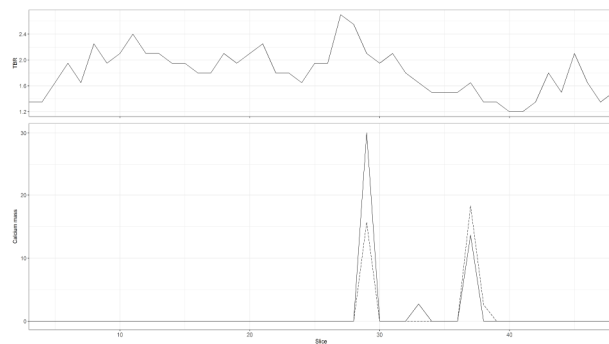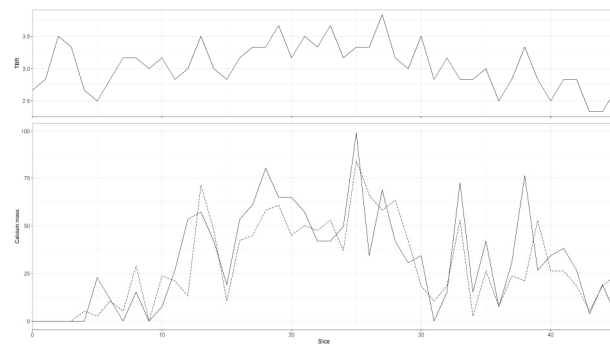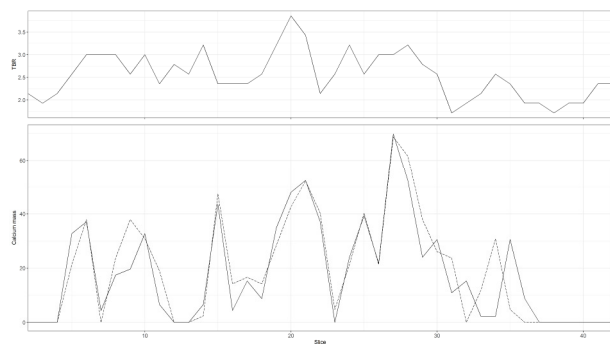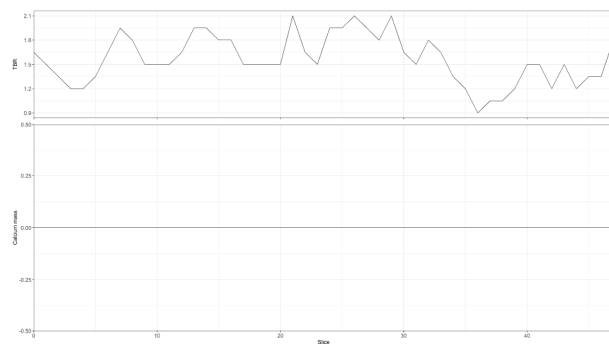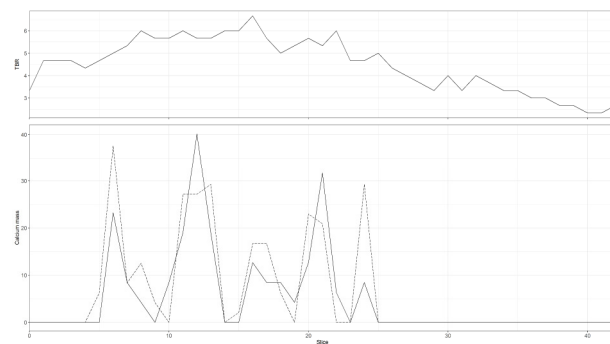



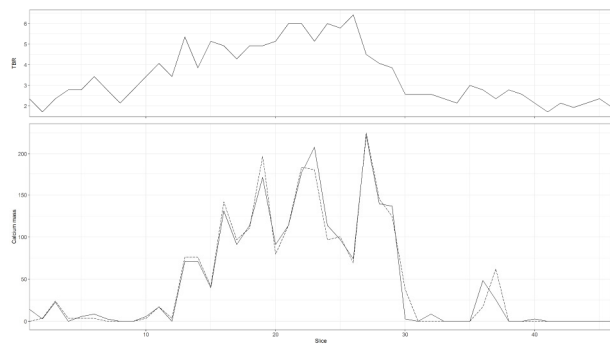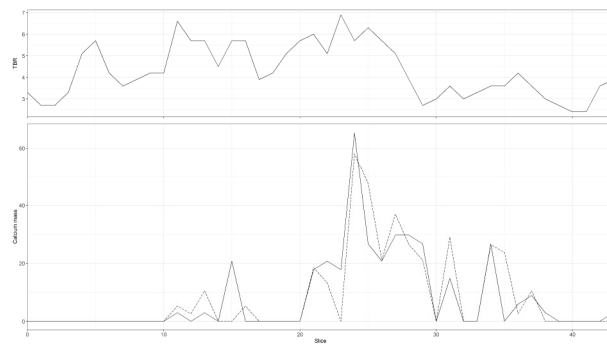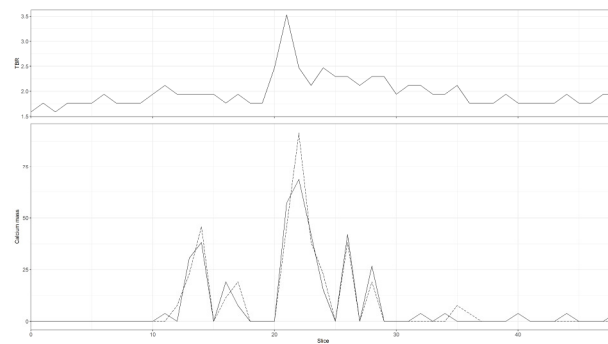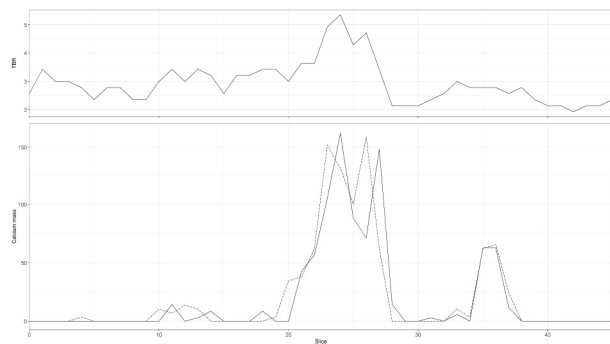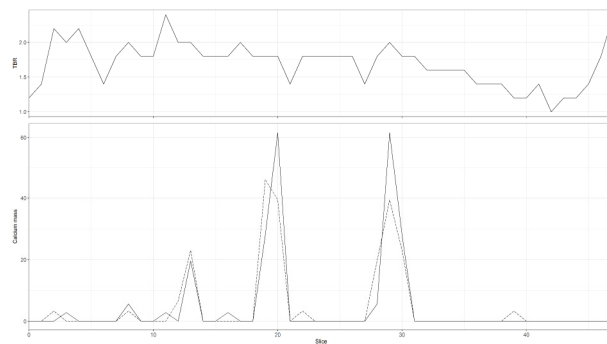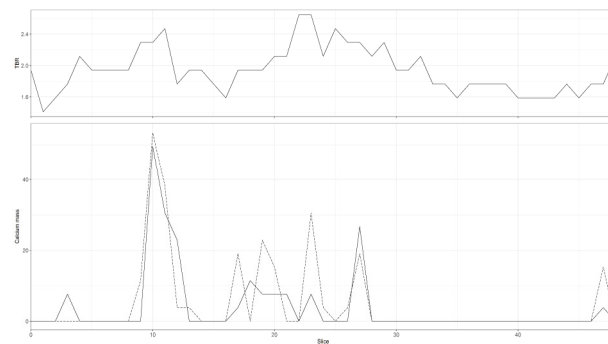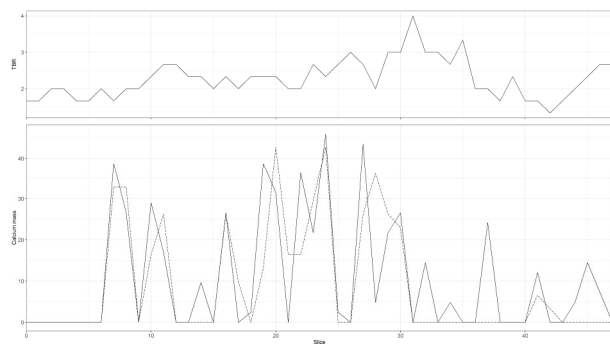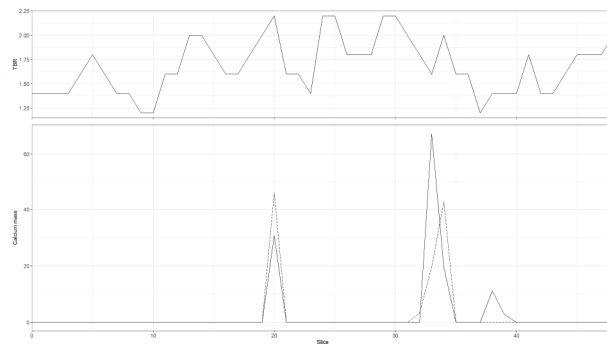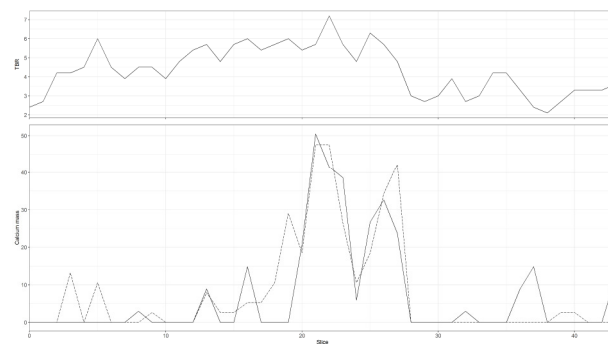

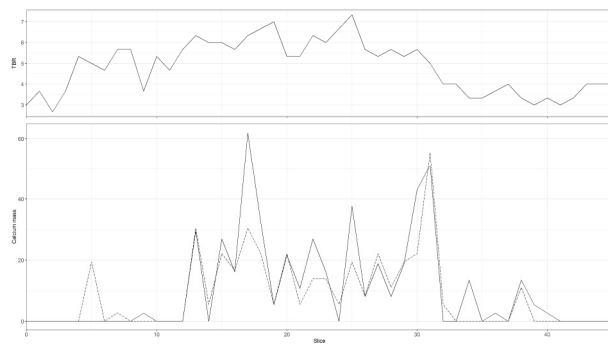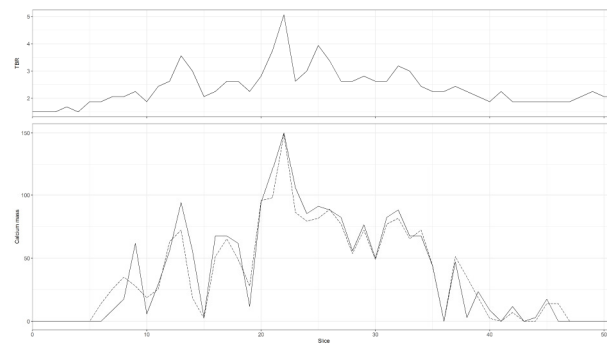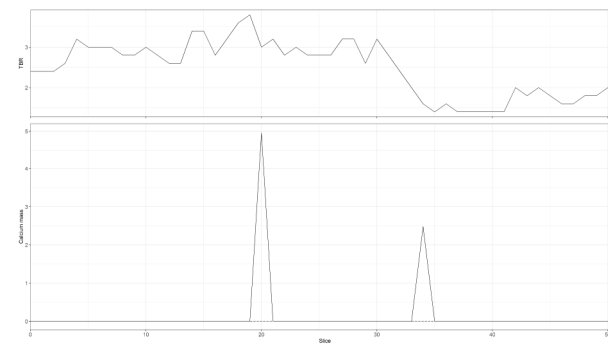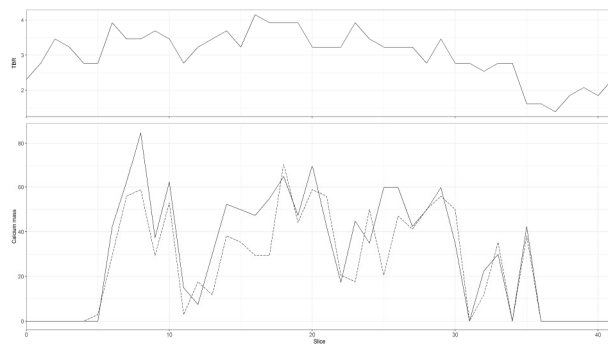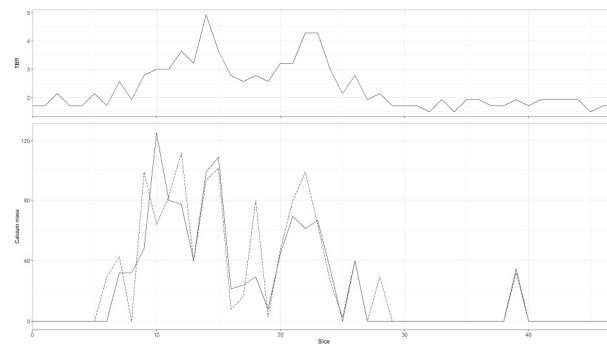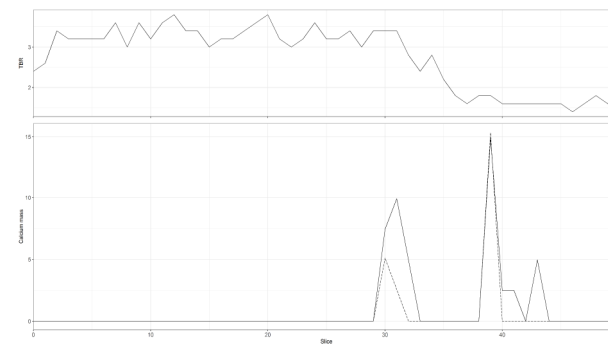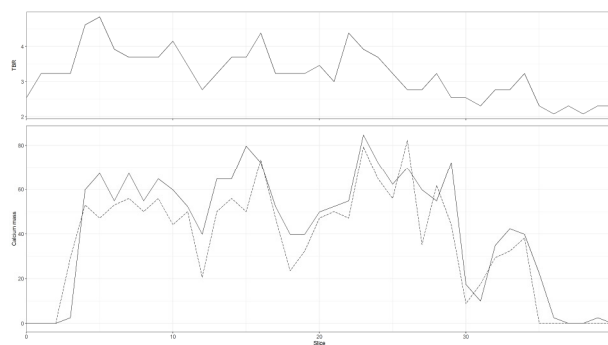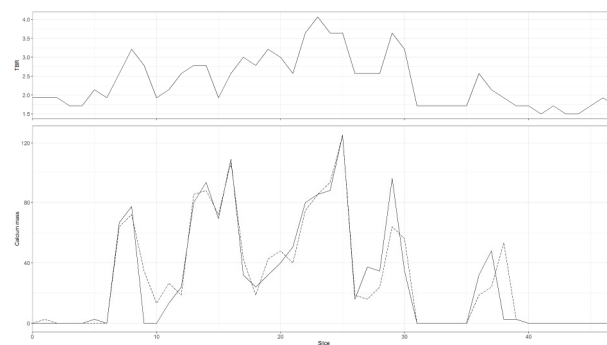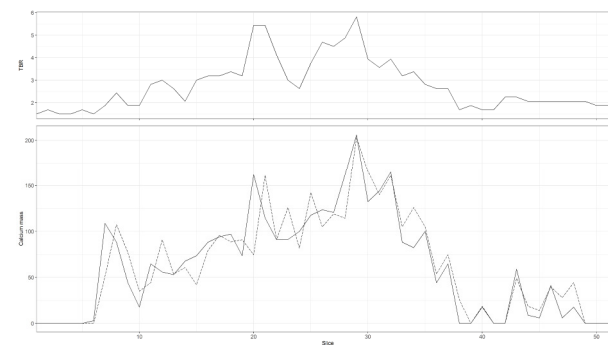

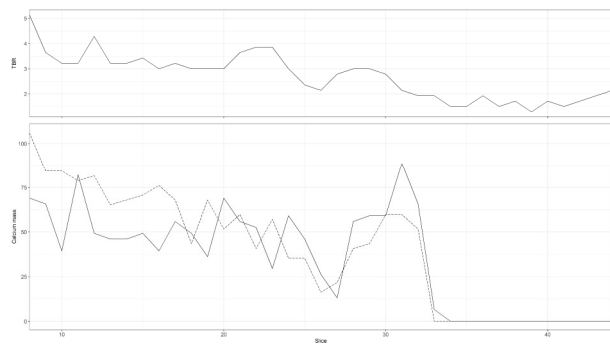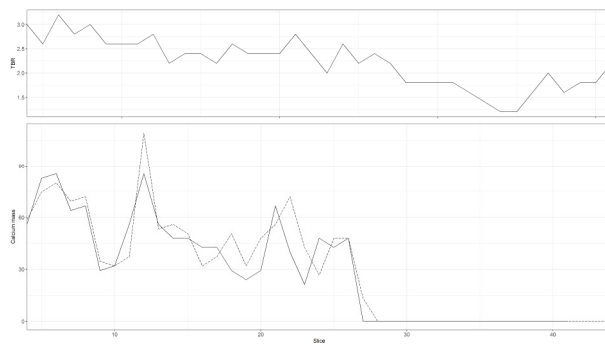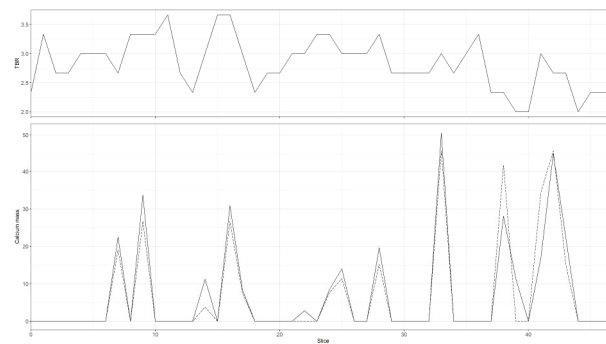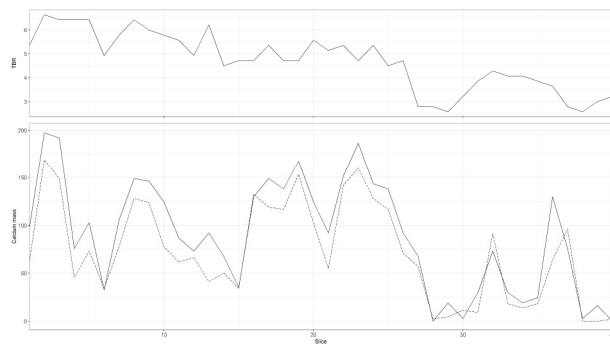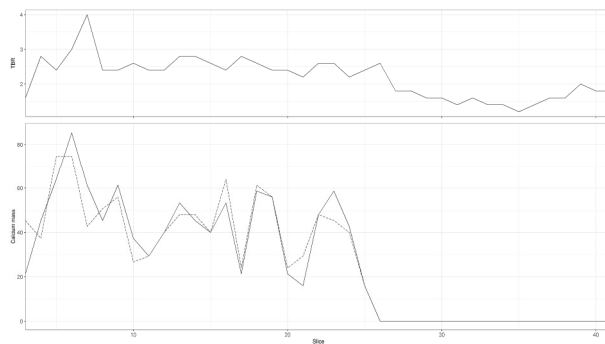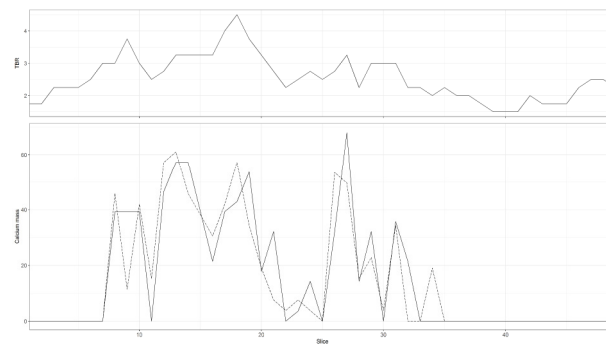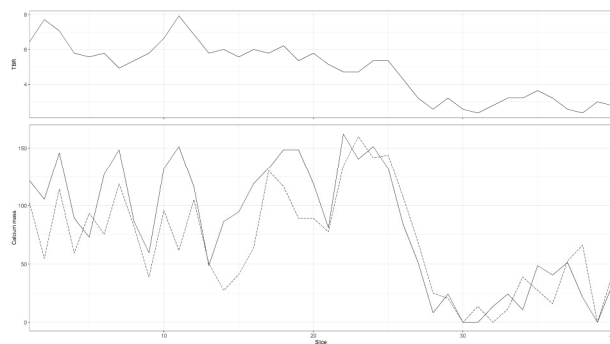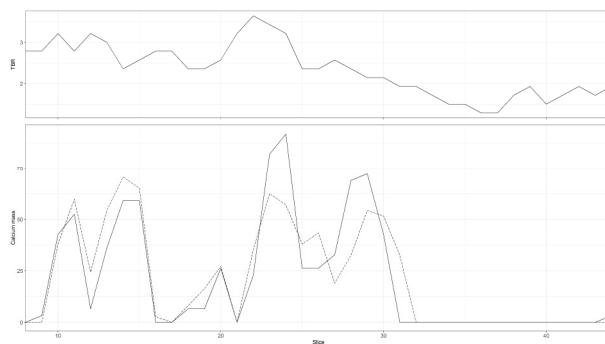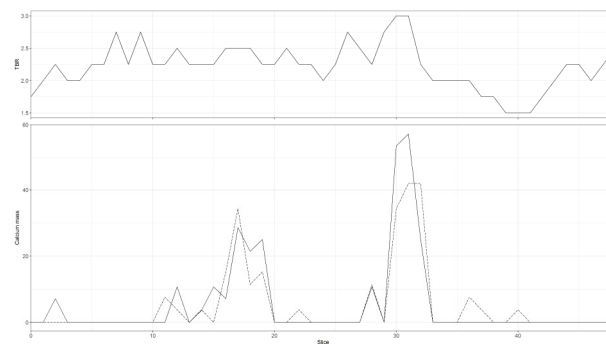

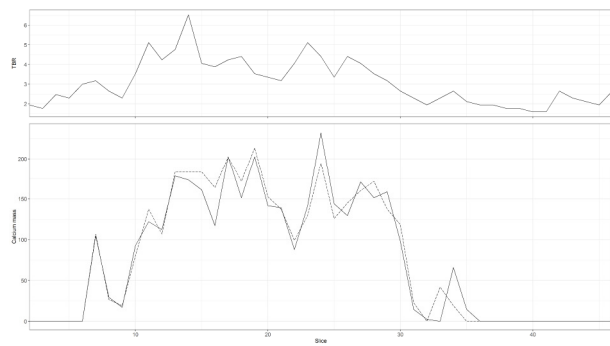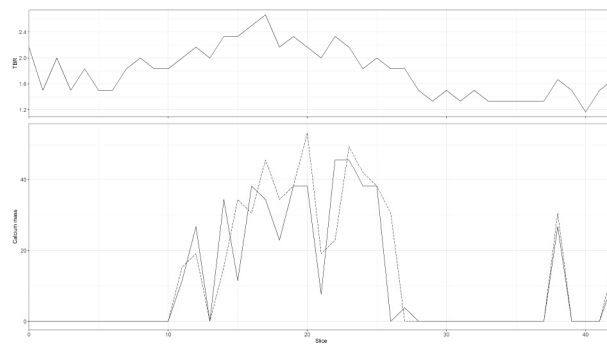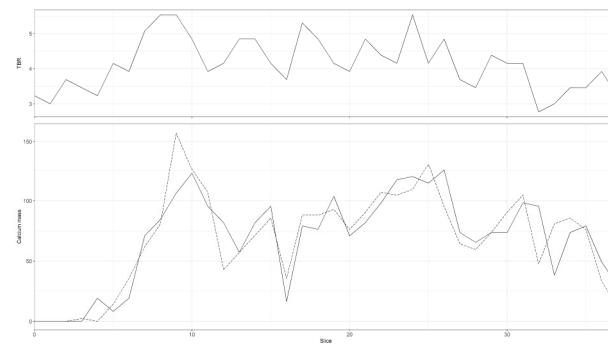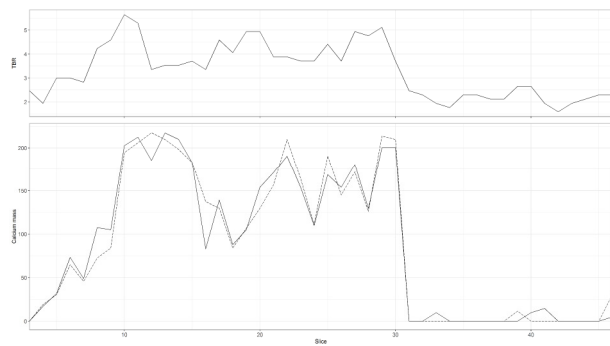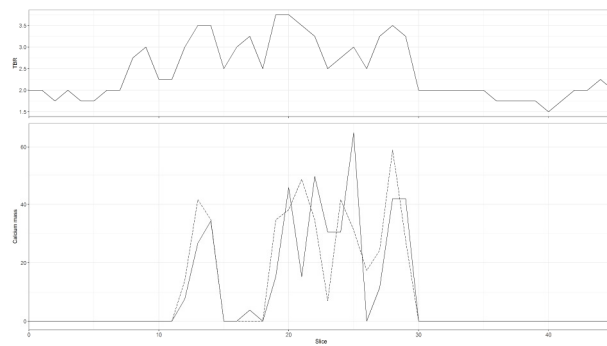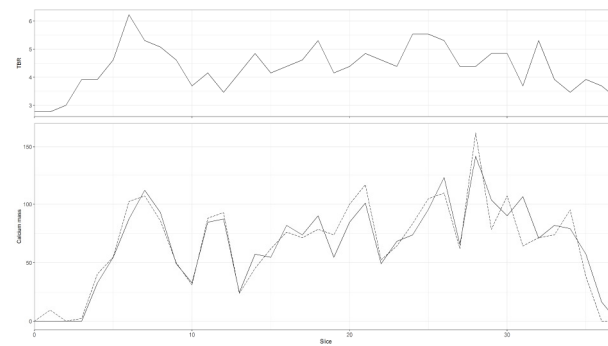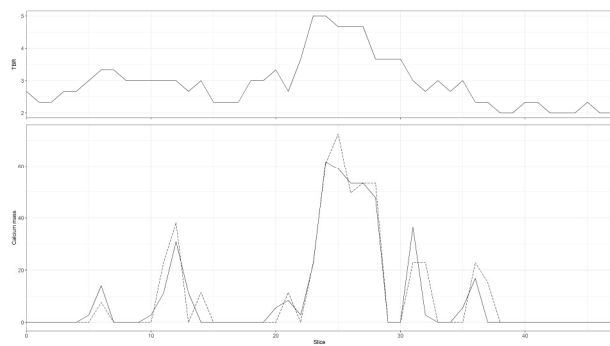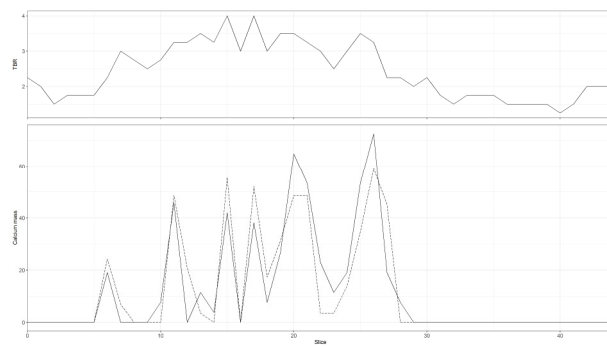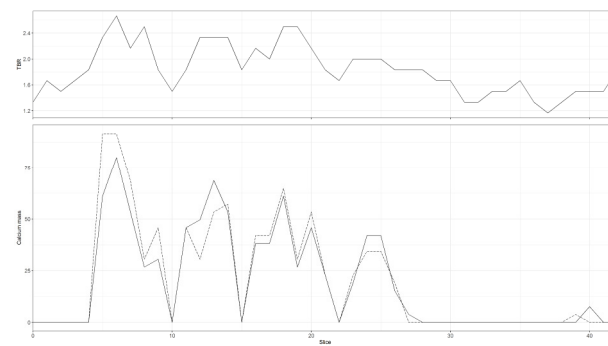

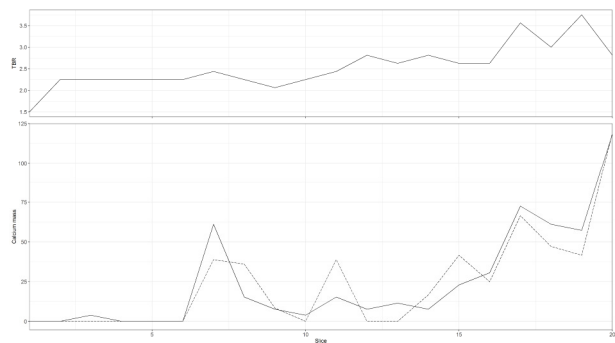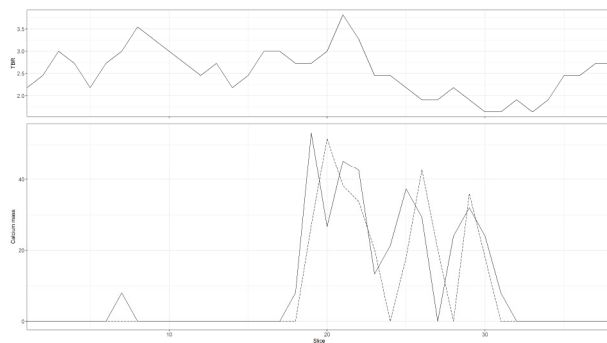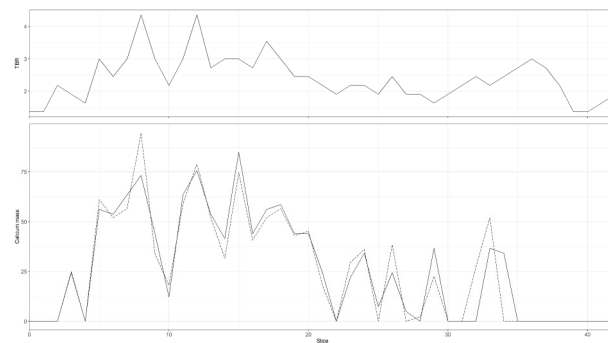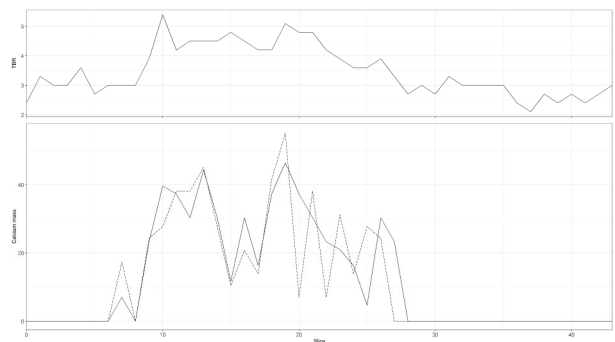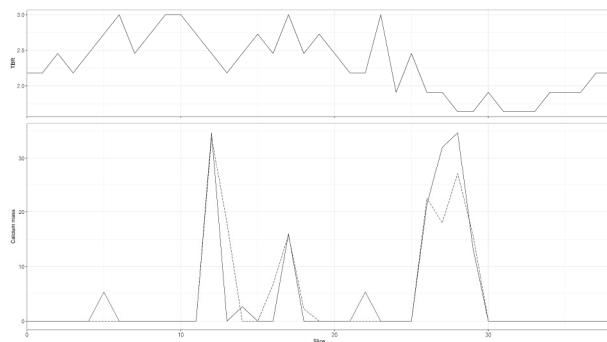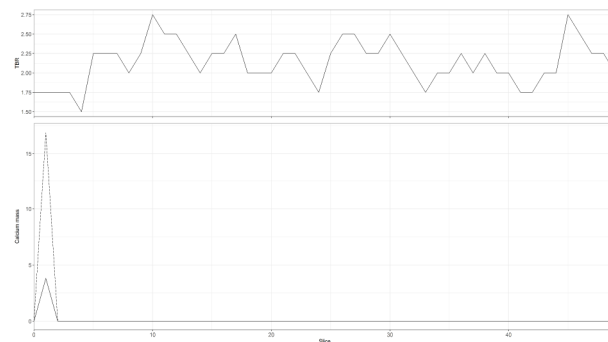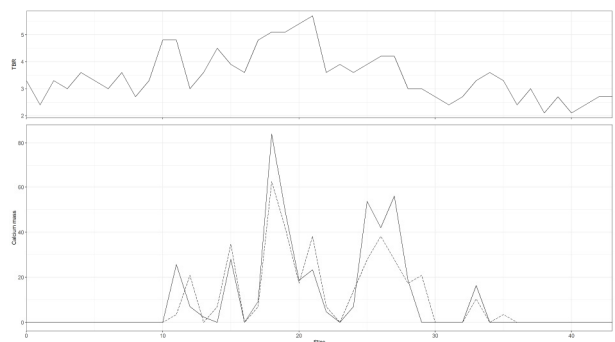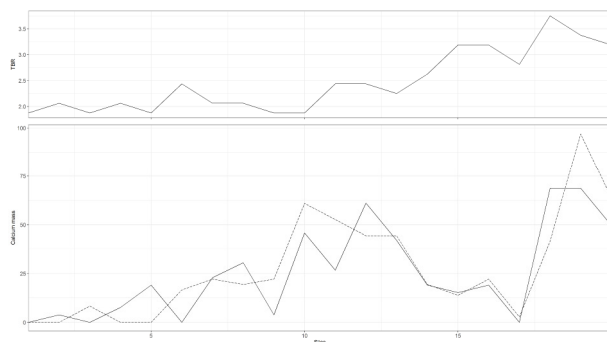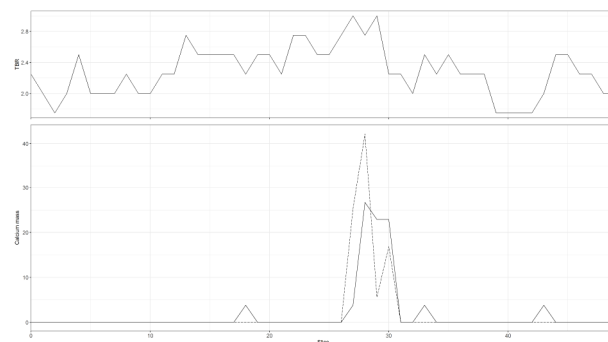

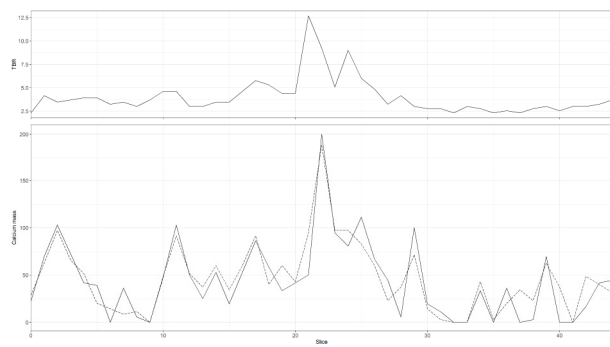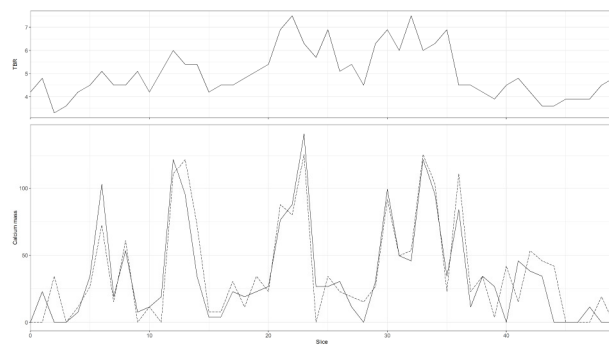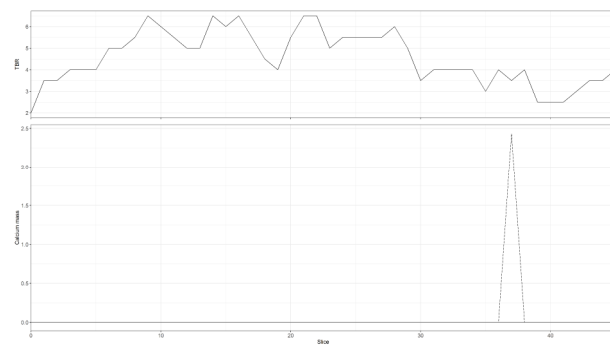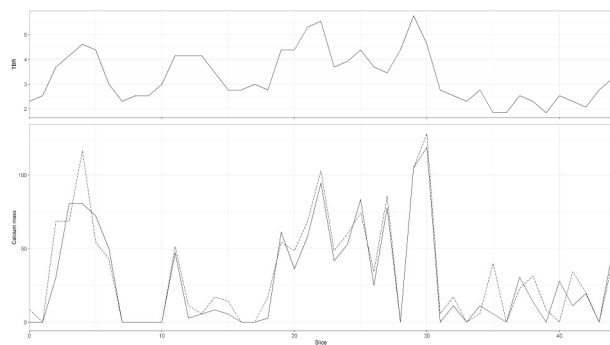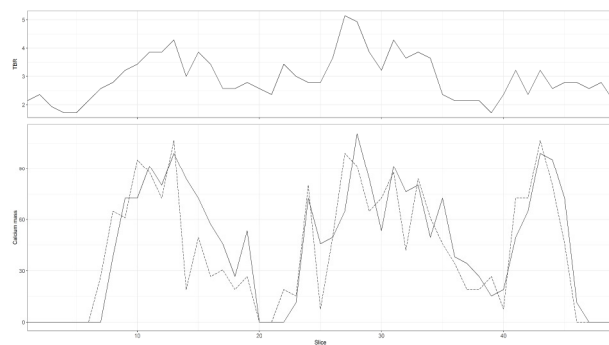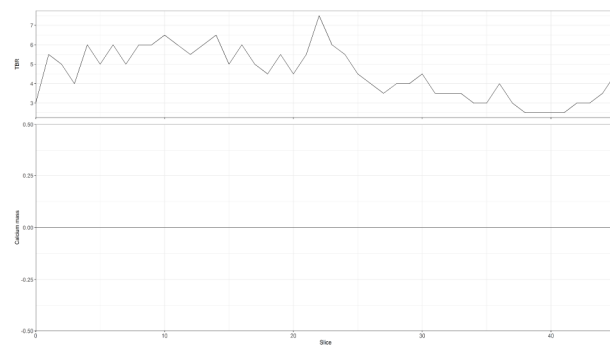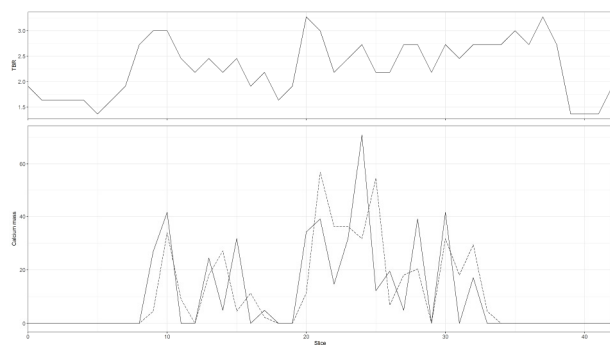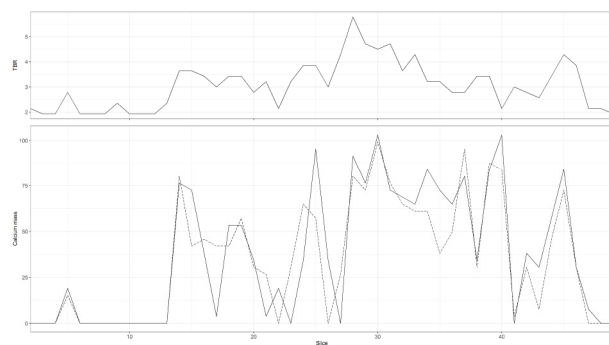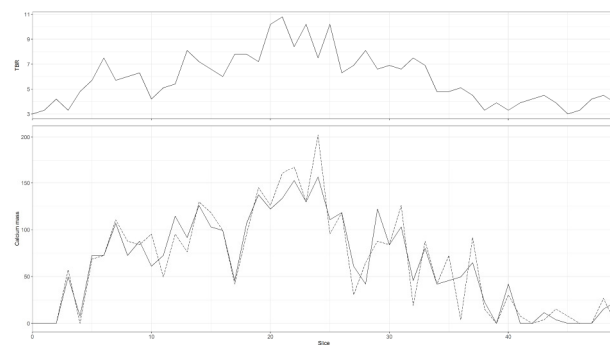

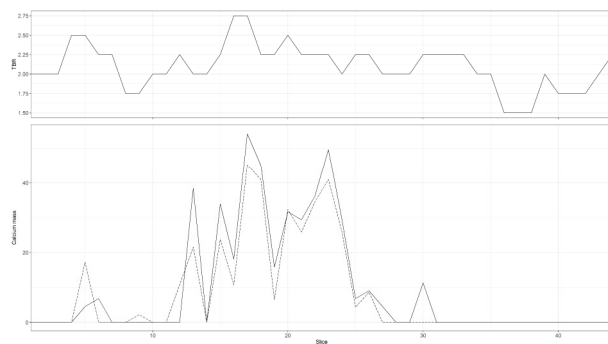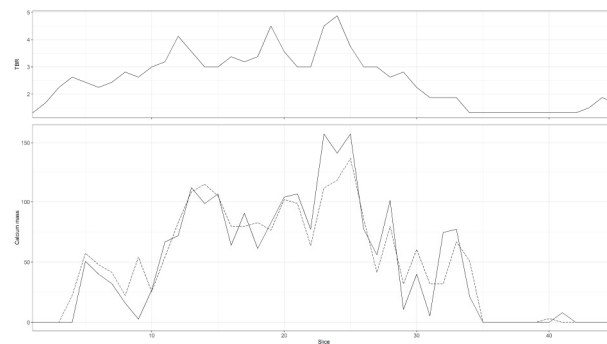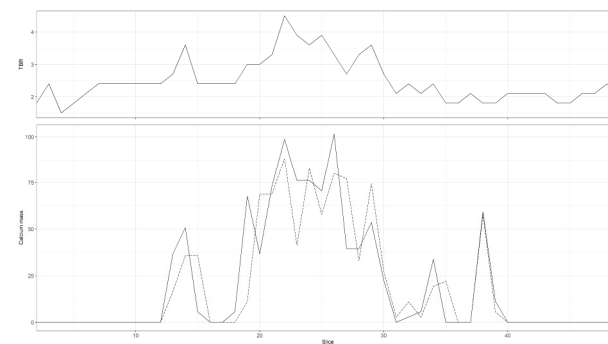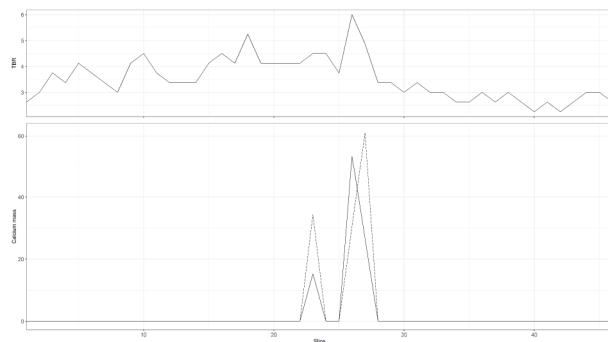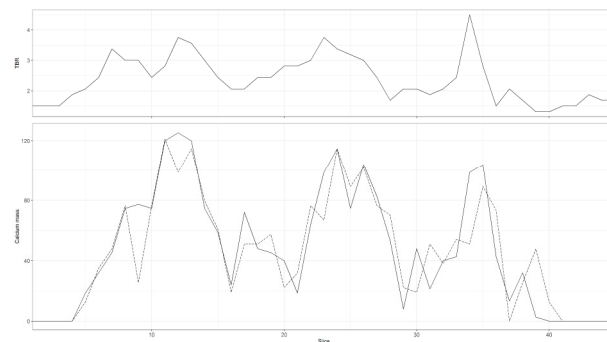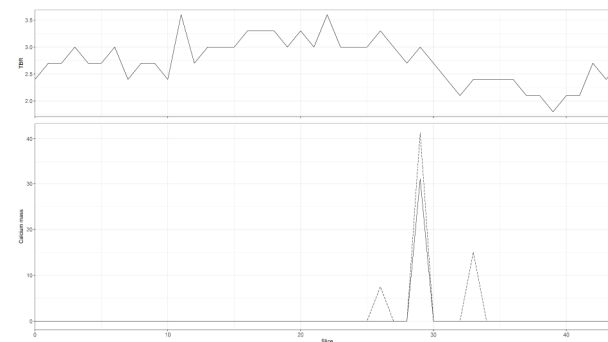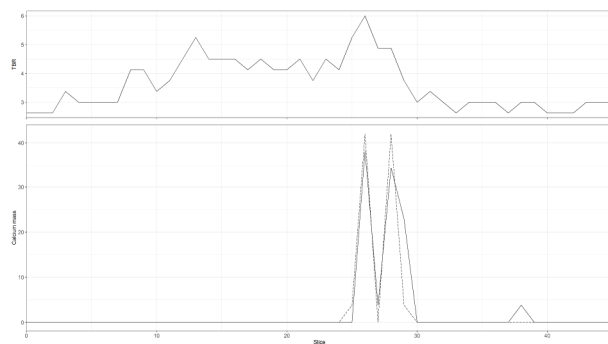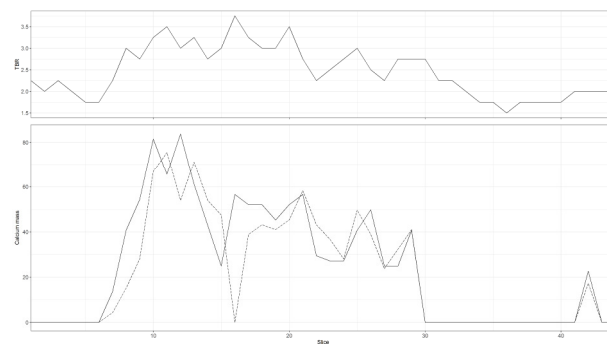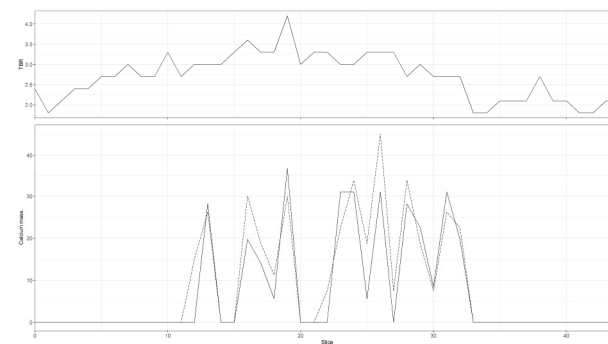

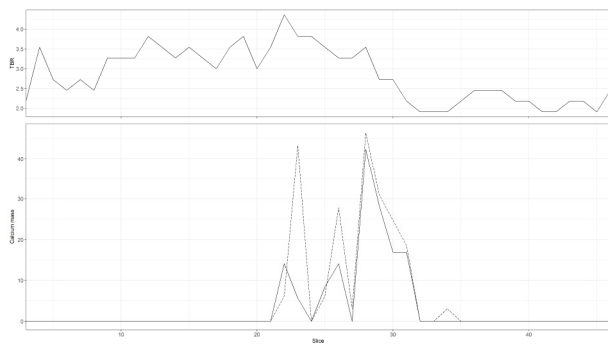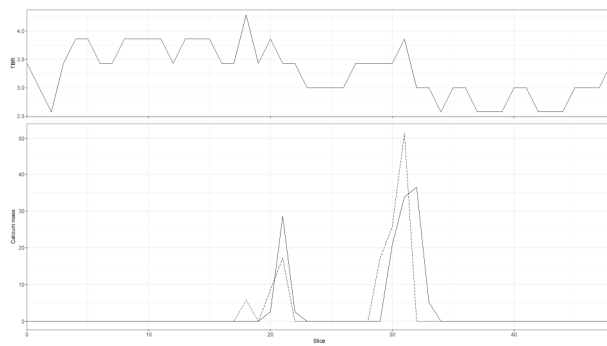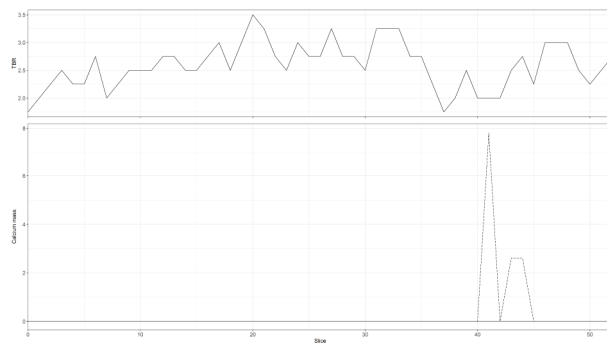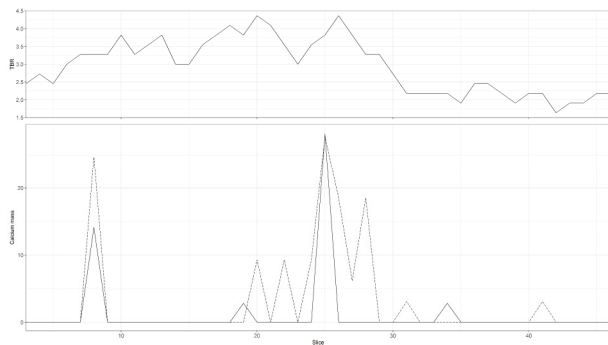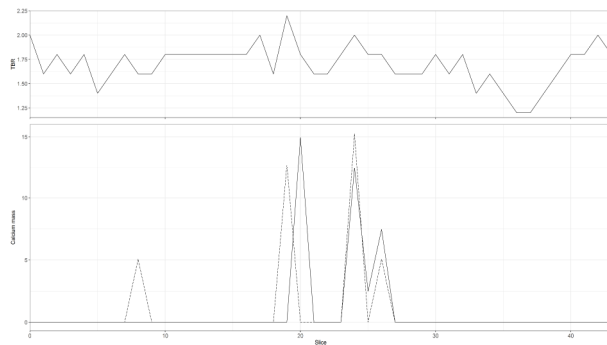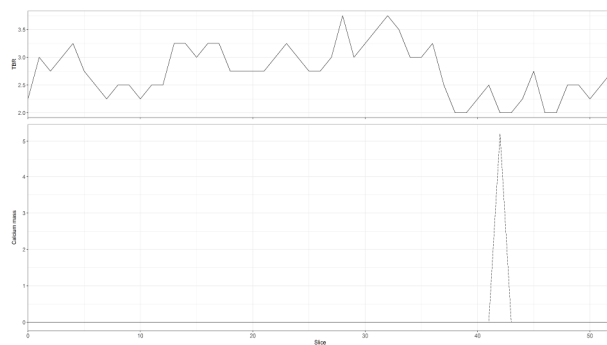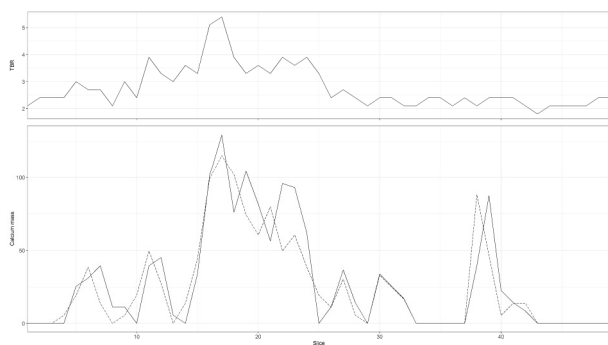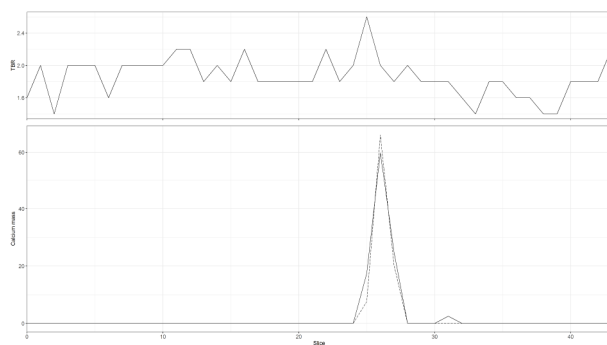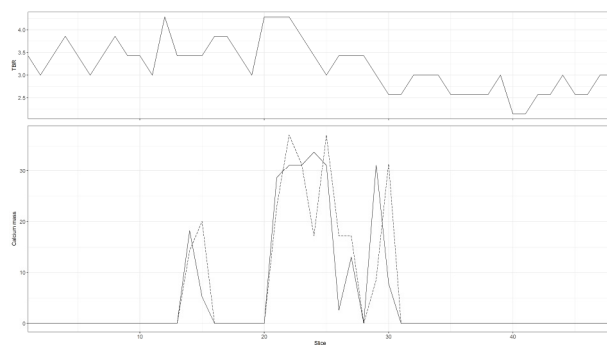

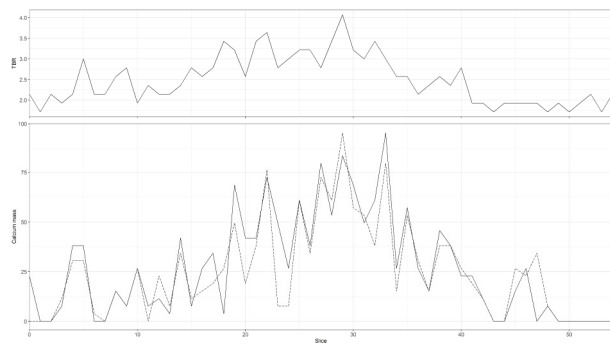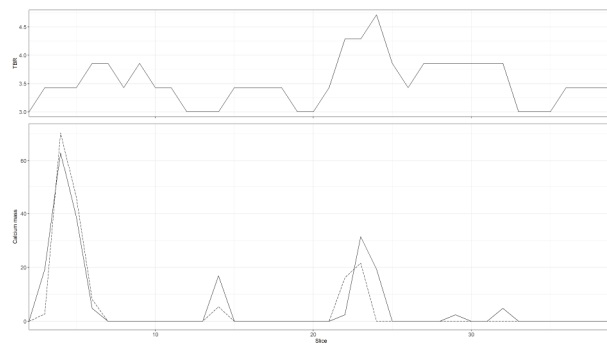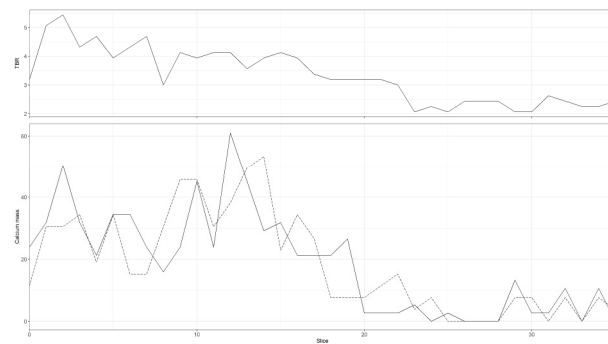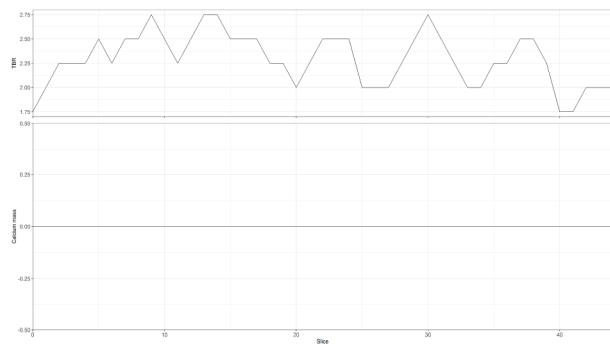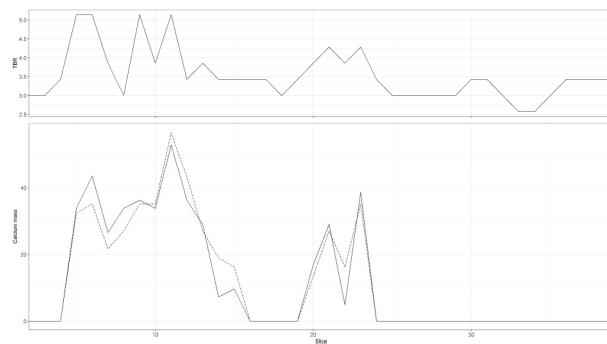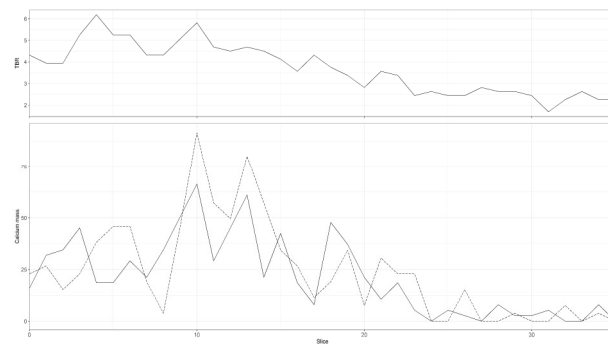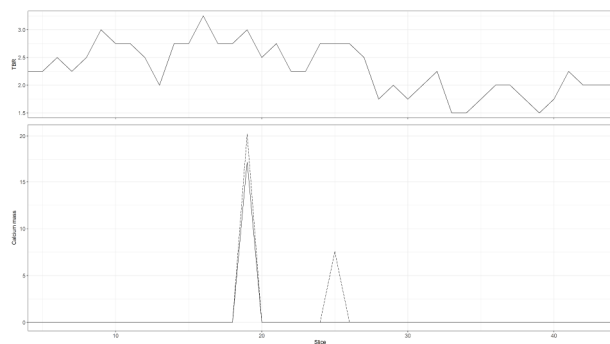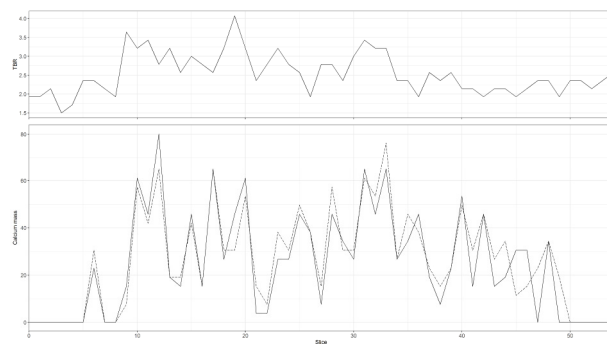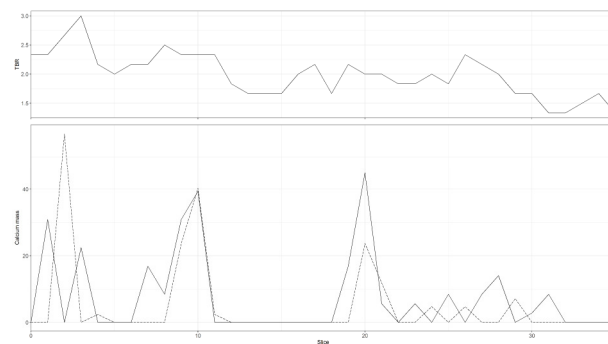

Supplement: Supplementary file 1 — Online Appendix 1: Plots of relationship between TBR at baseline and the calcium mass at baseline and follow-up for the TEMP study (PDF 6962 kb) [file 12350_2020_2031_MOESM1_ESM.pdf]

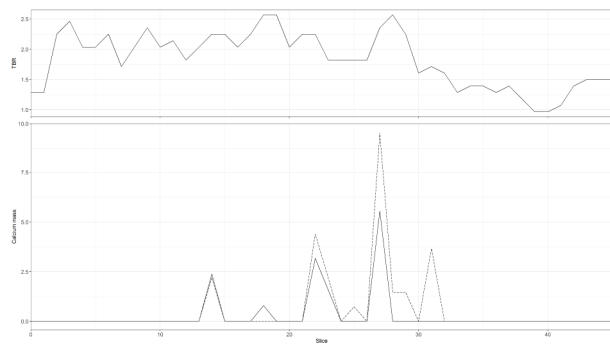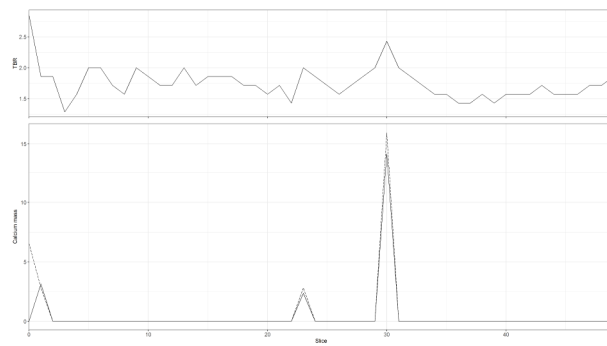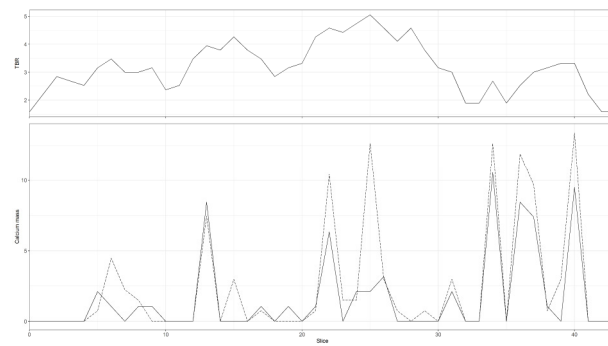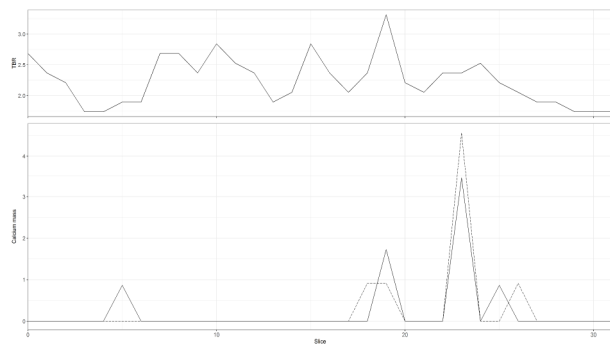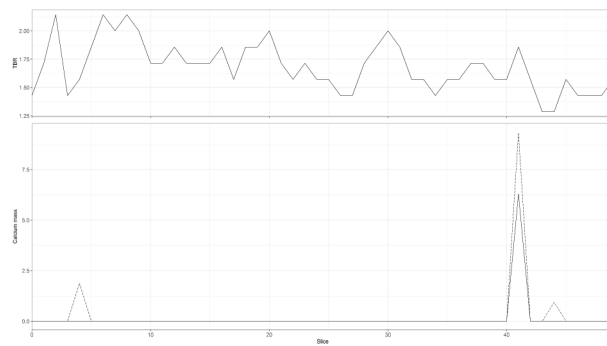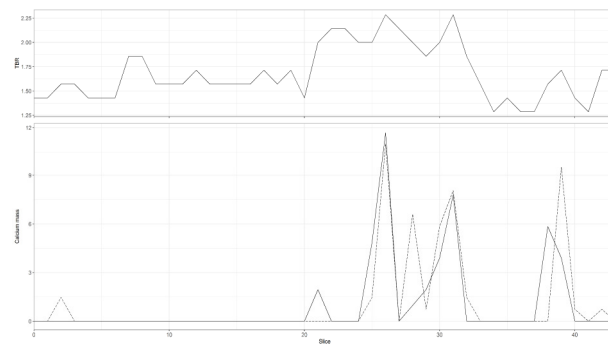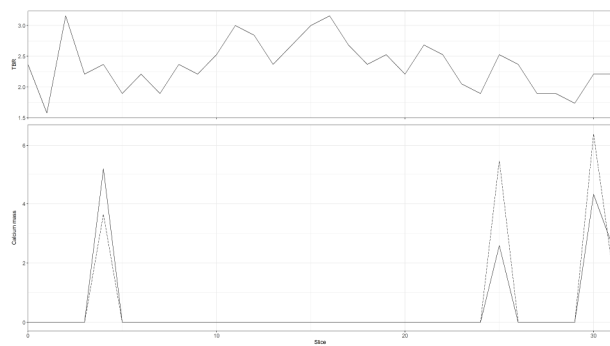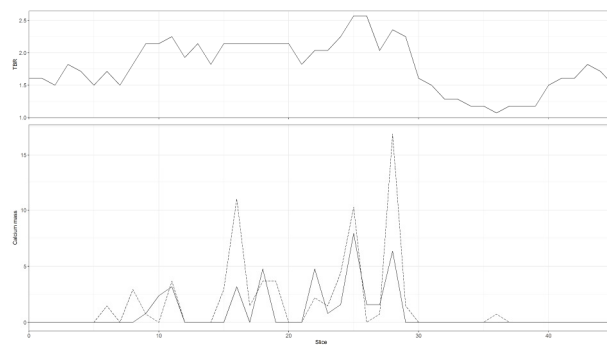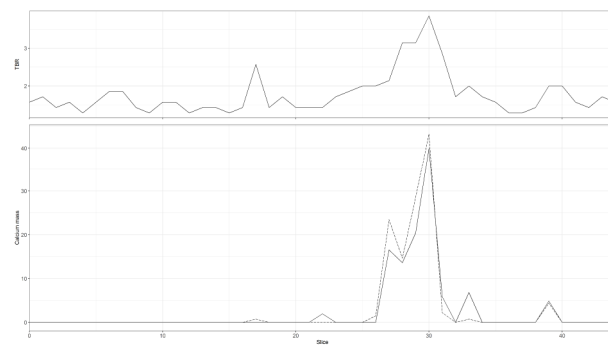

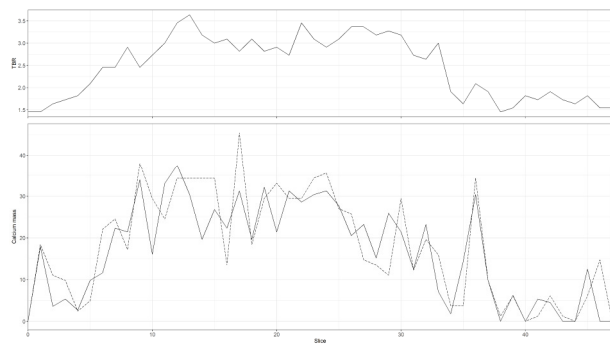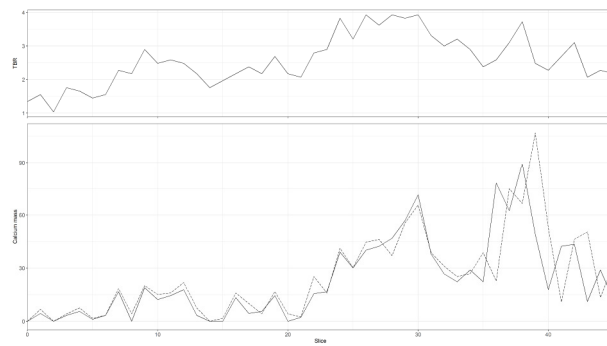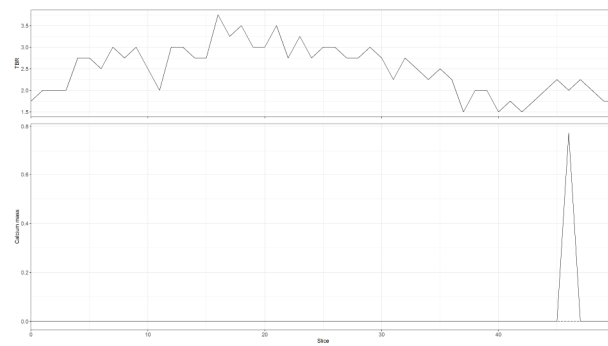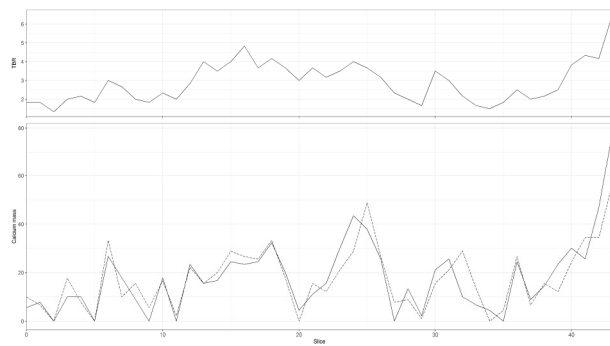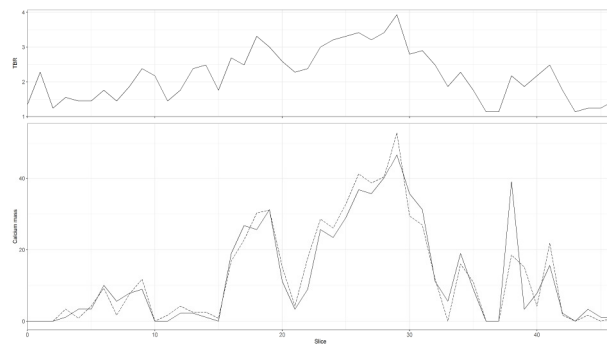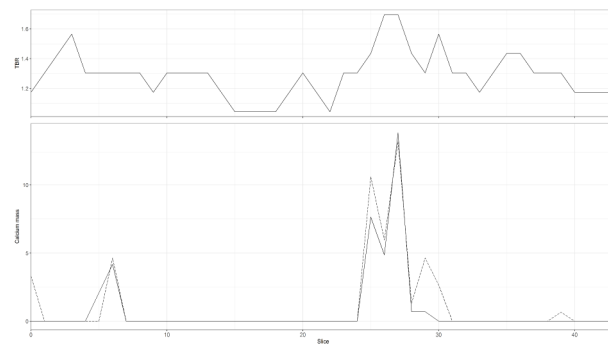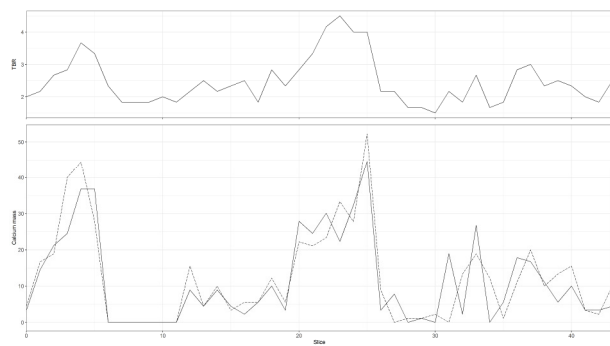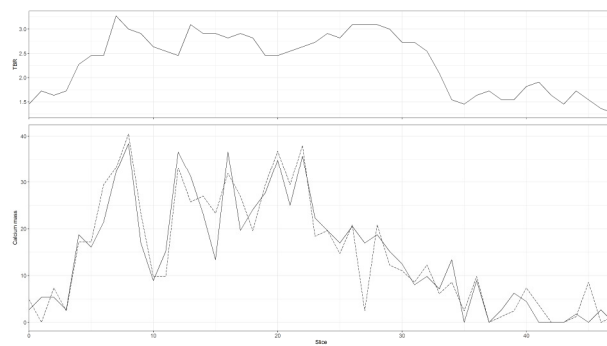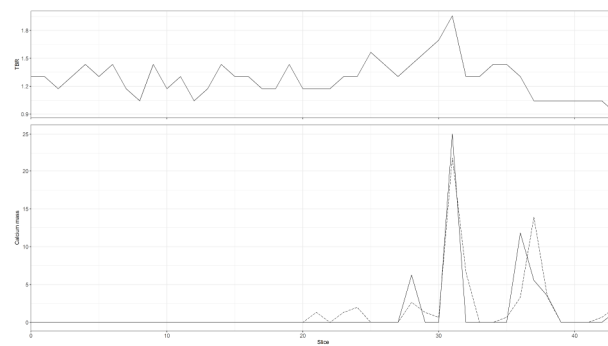

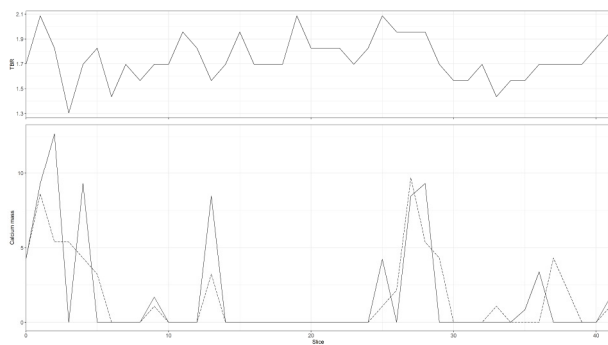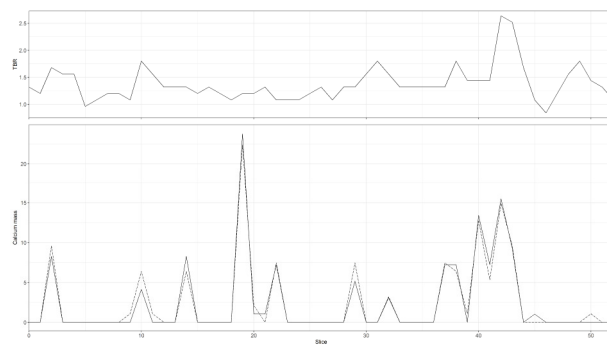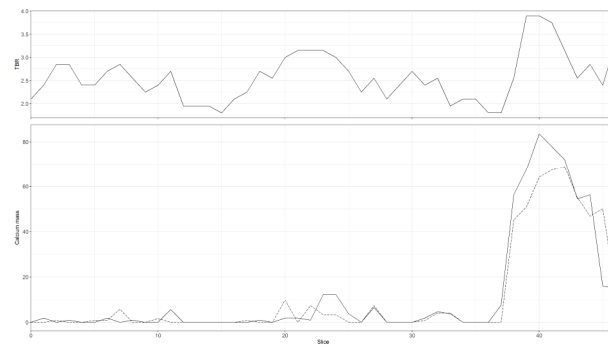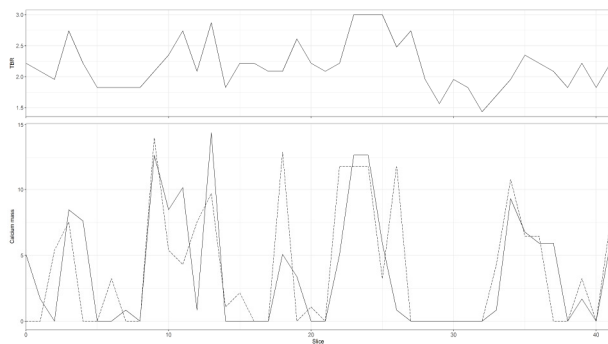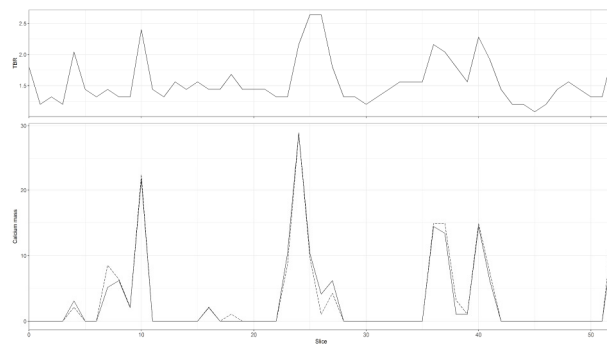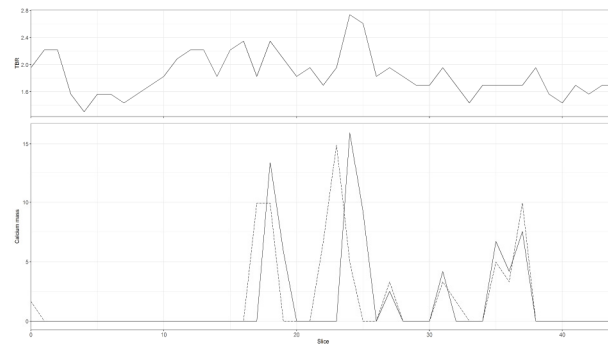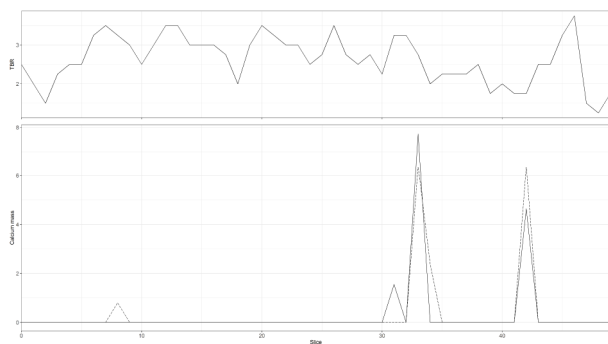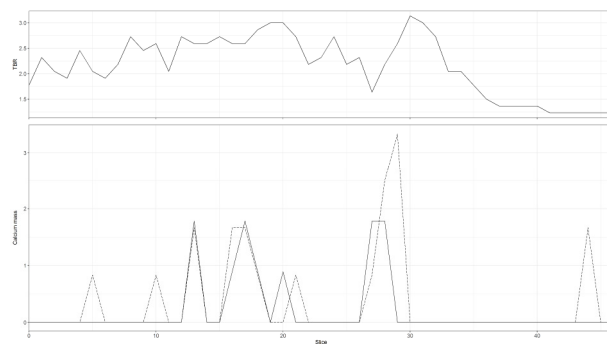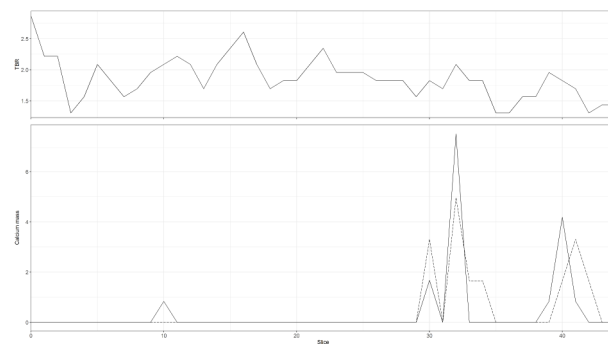

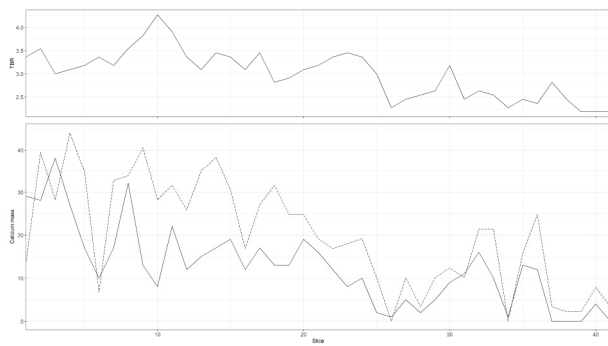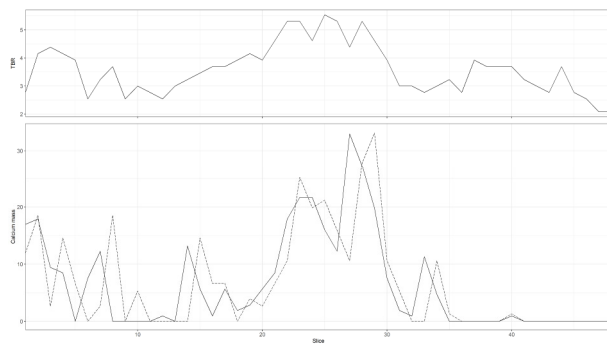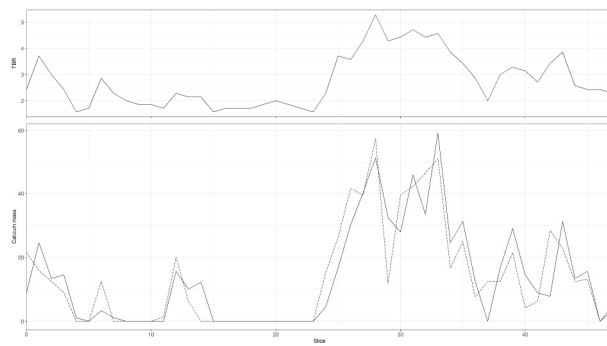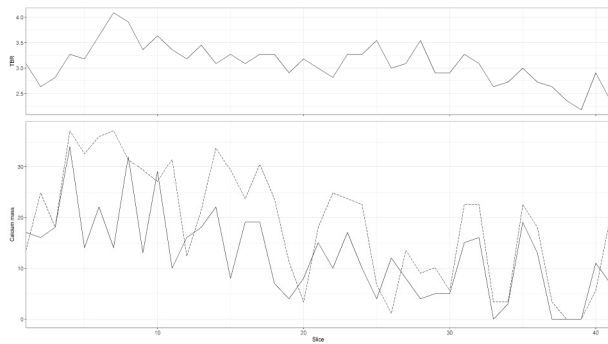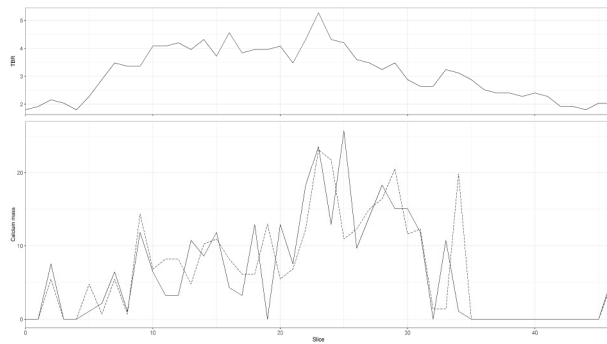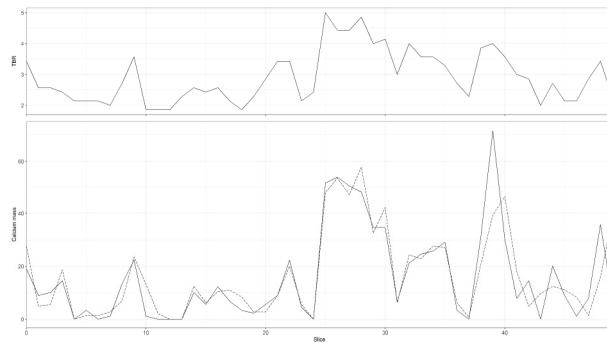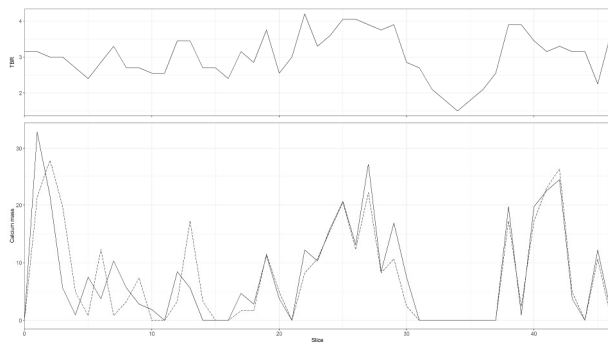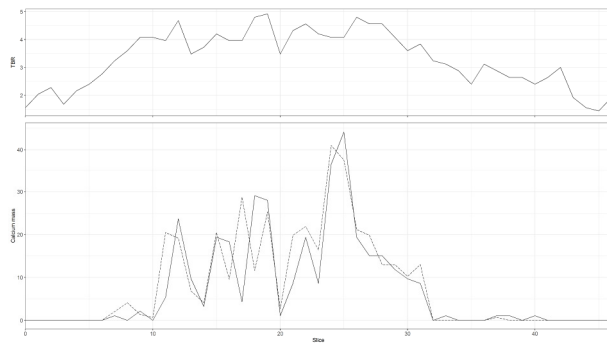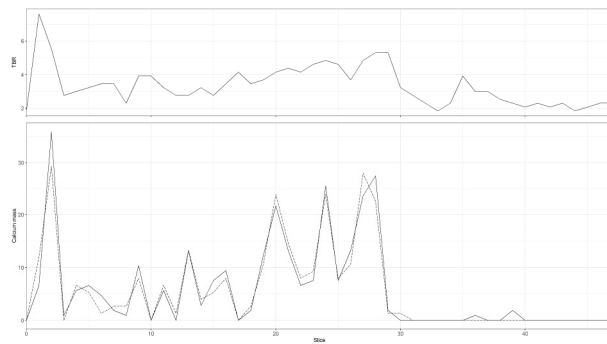

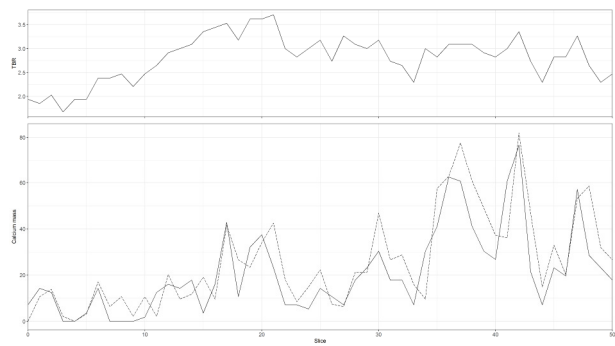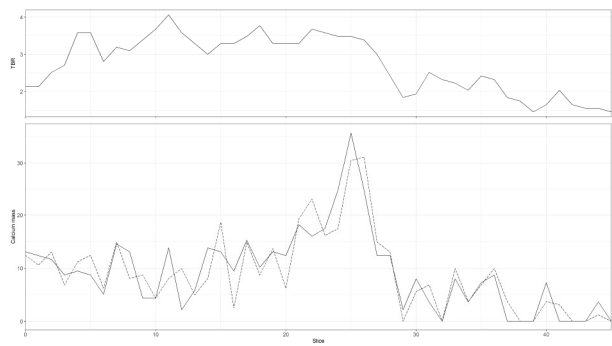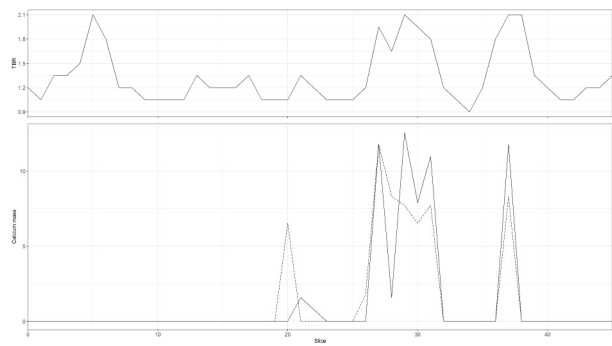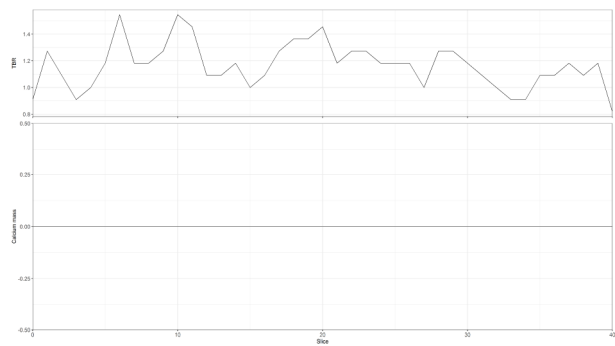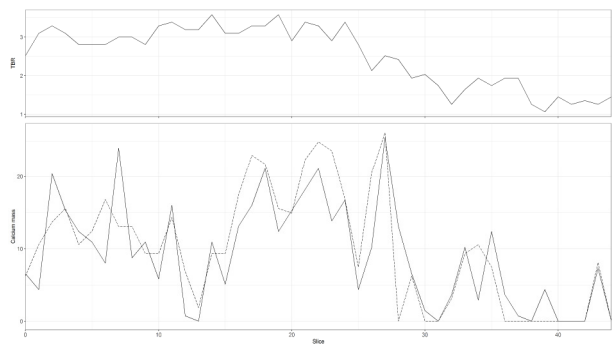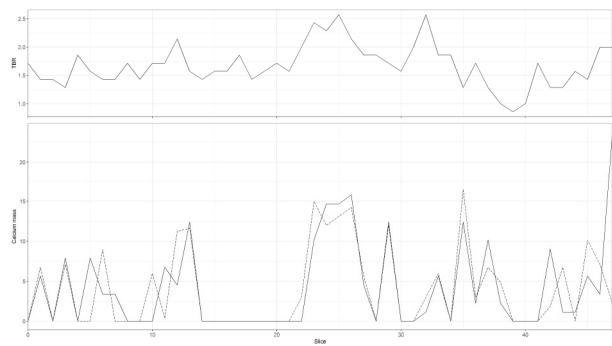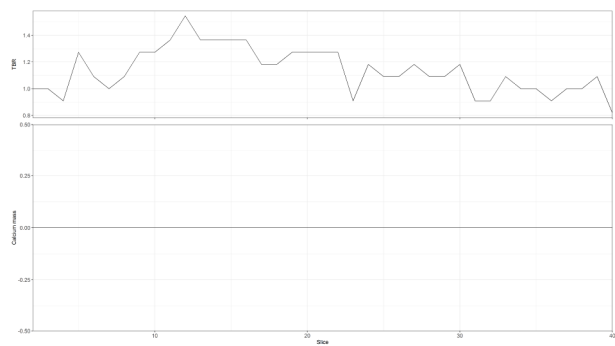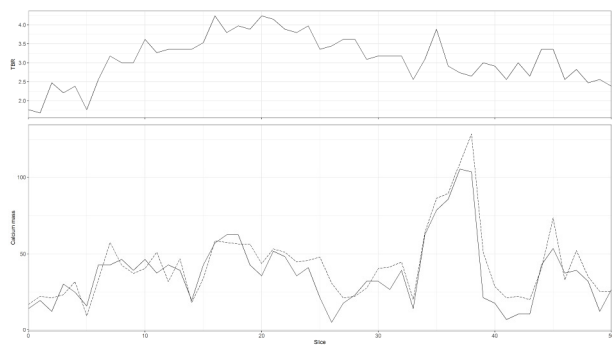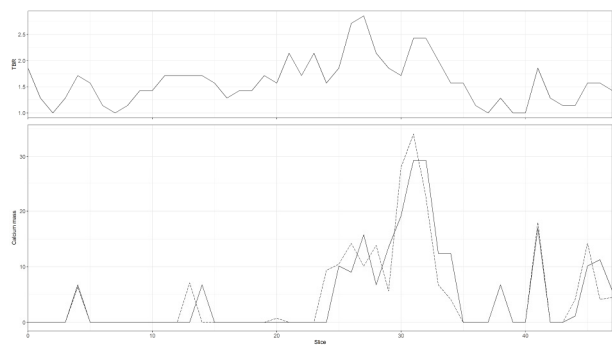

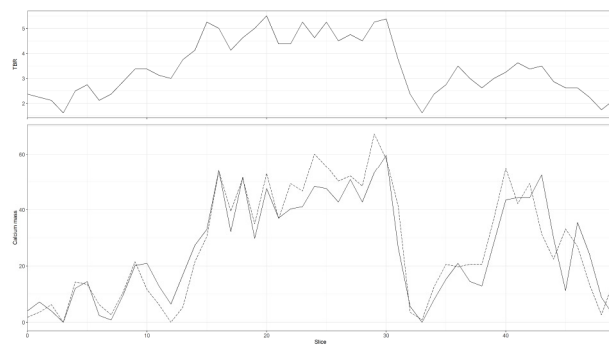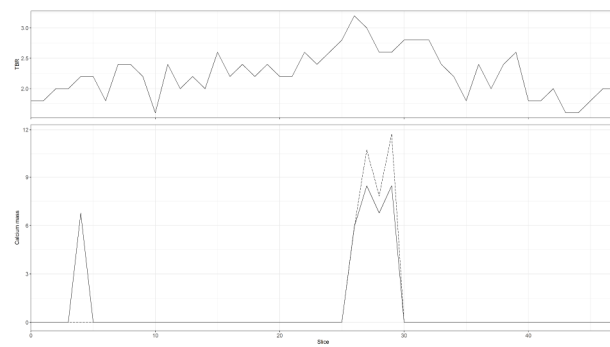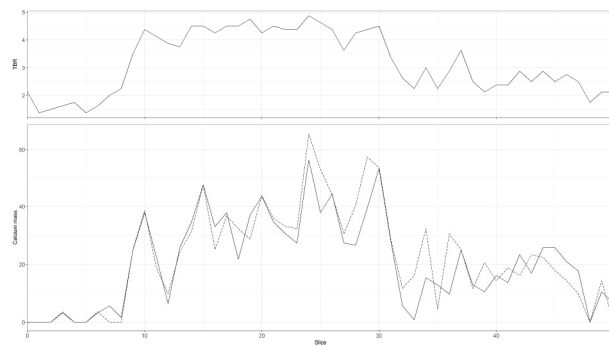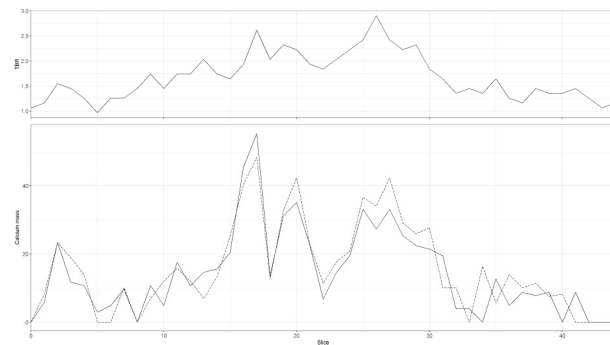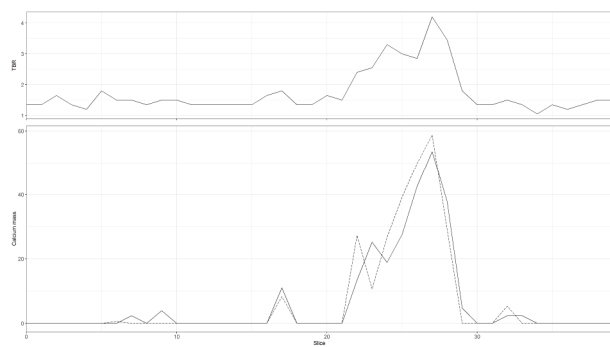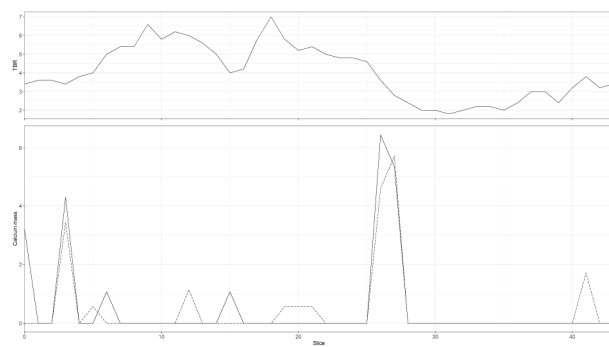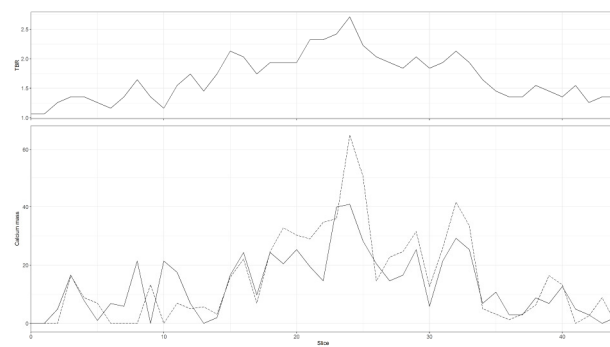

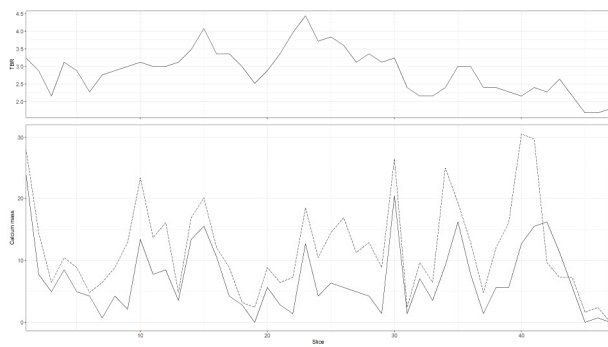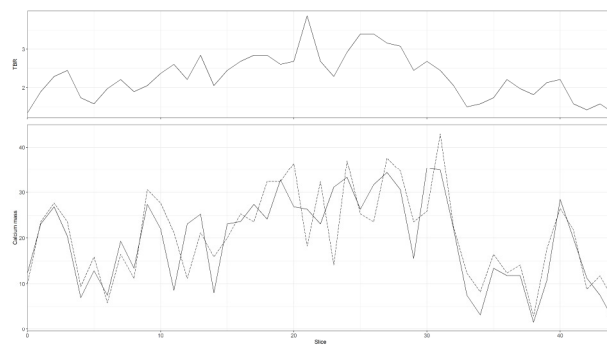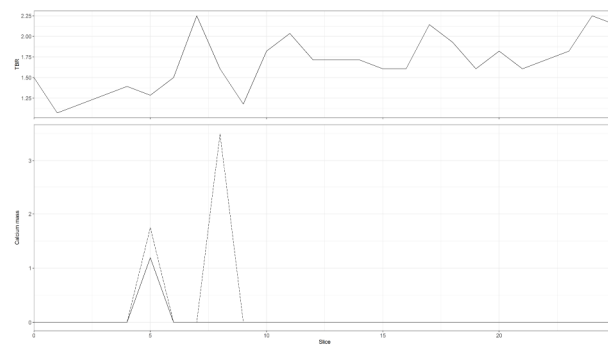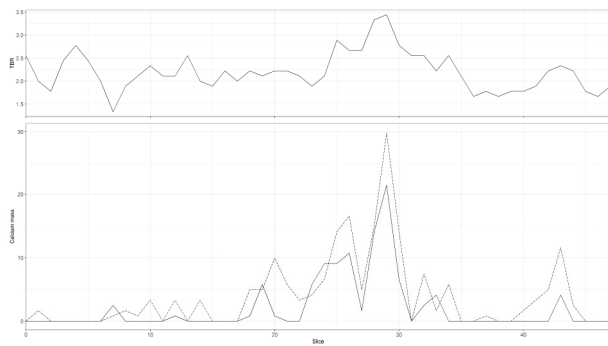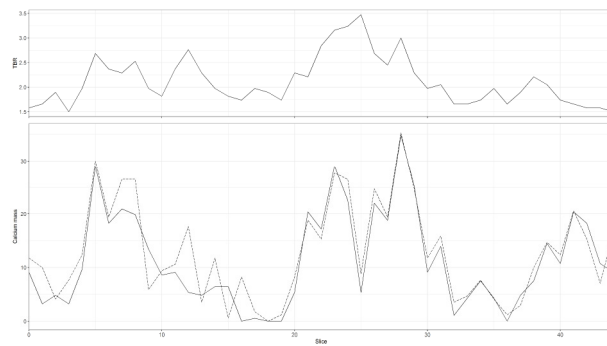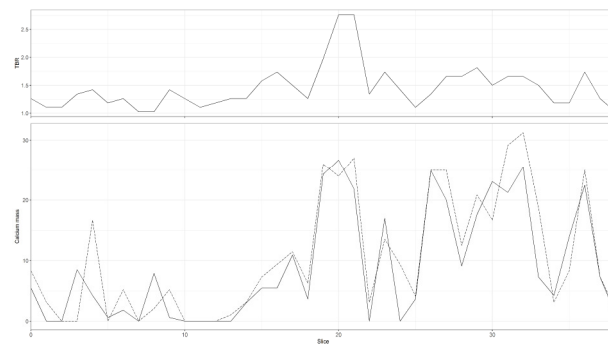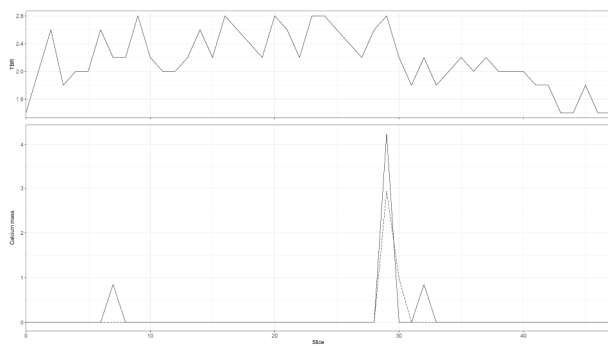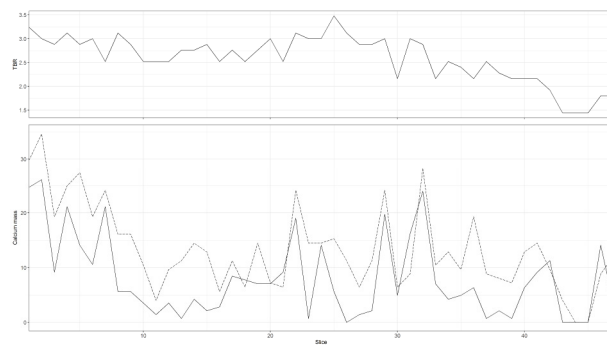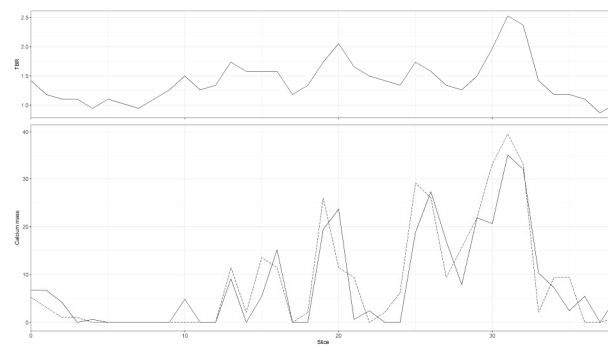

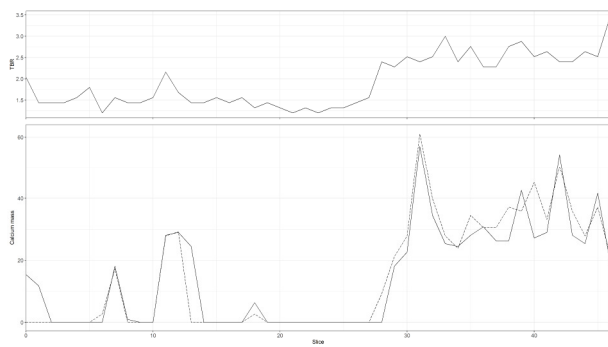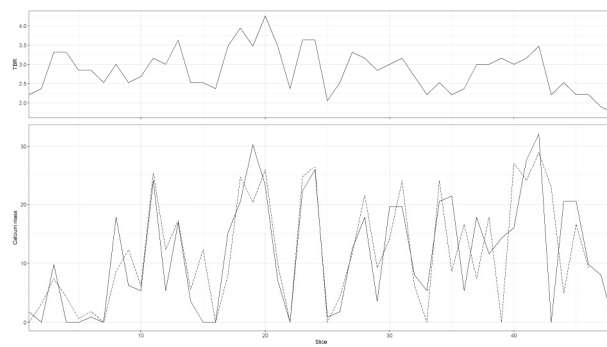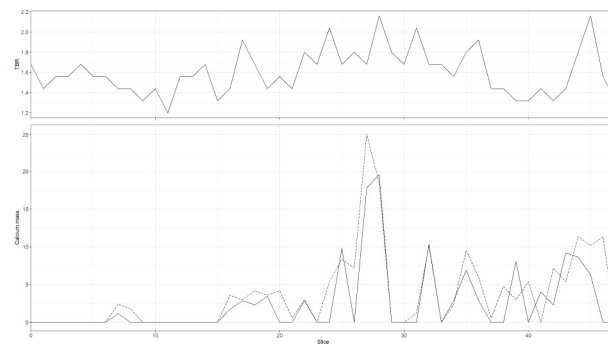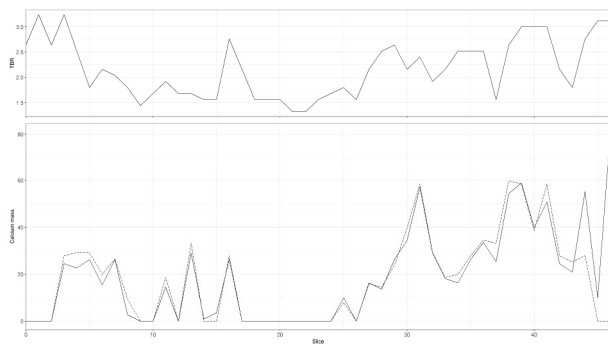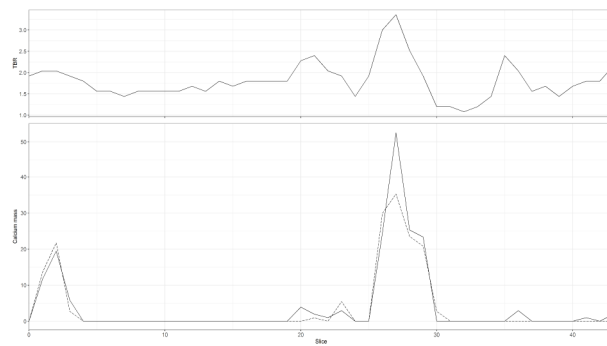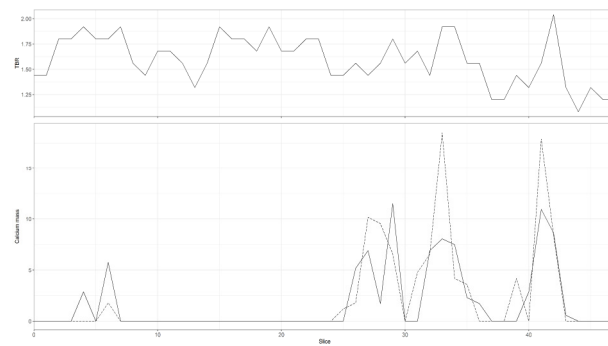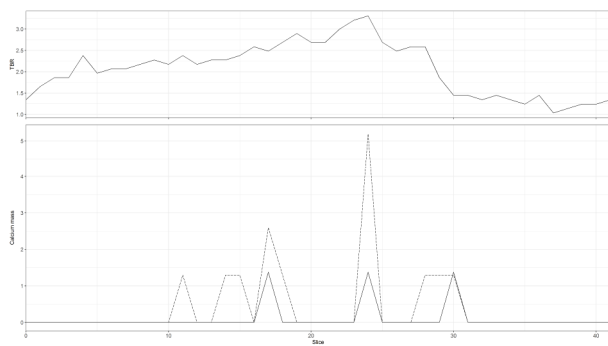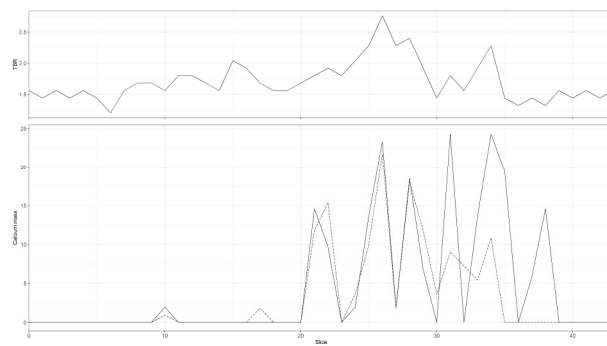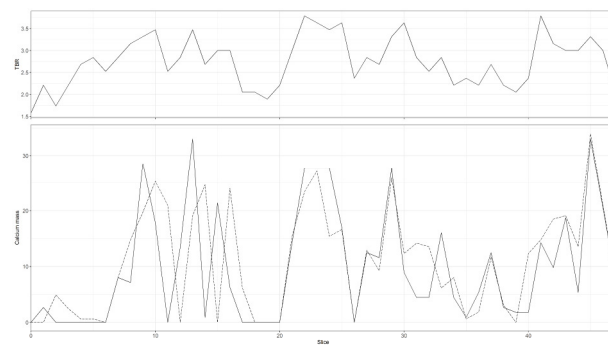

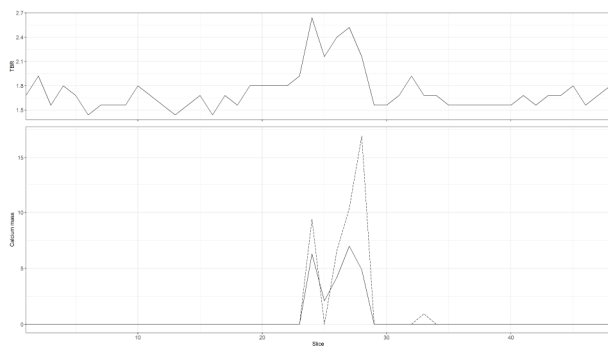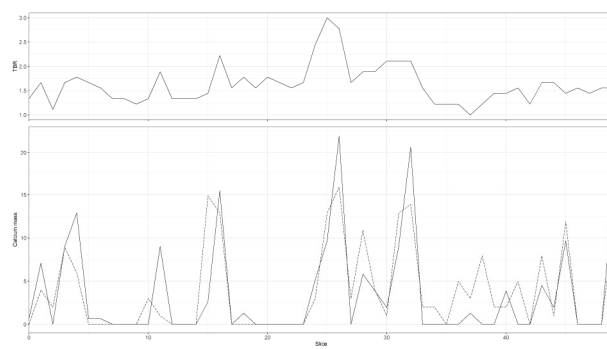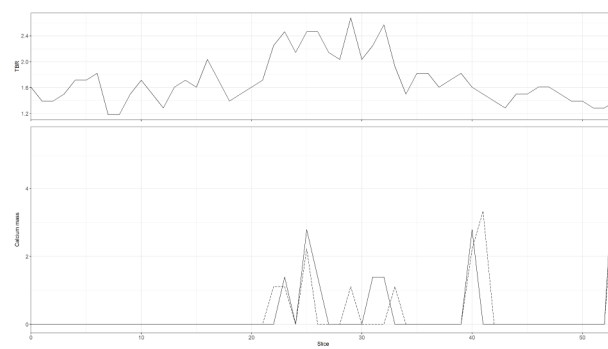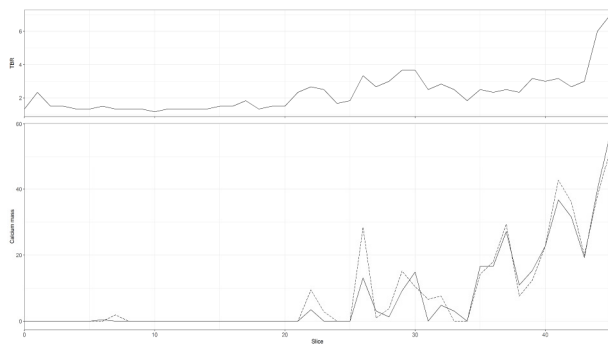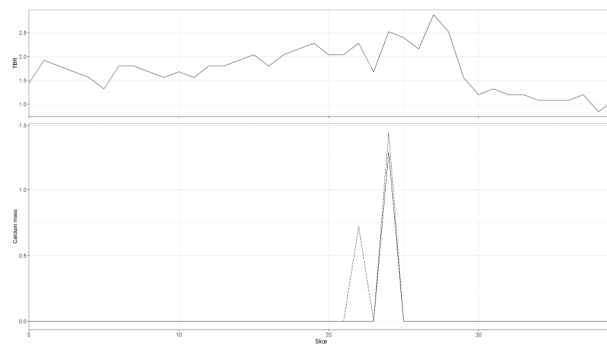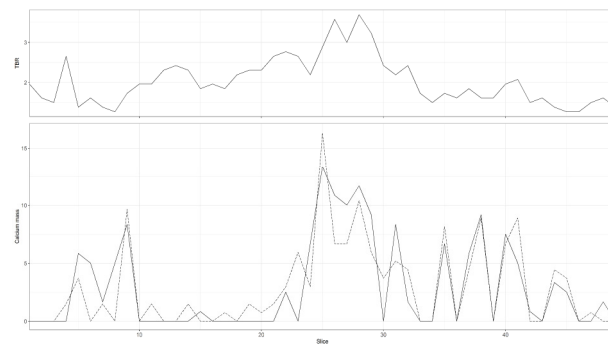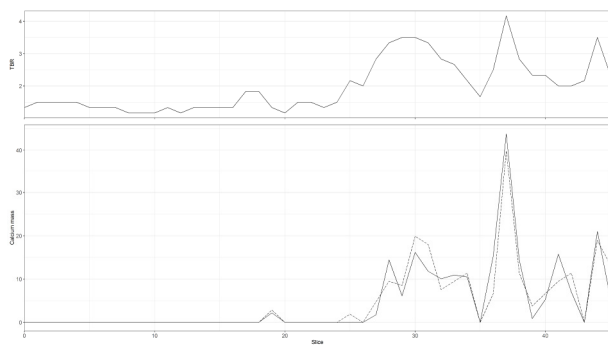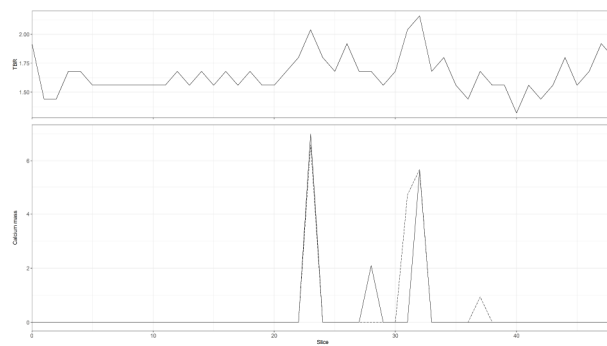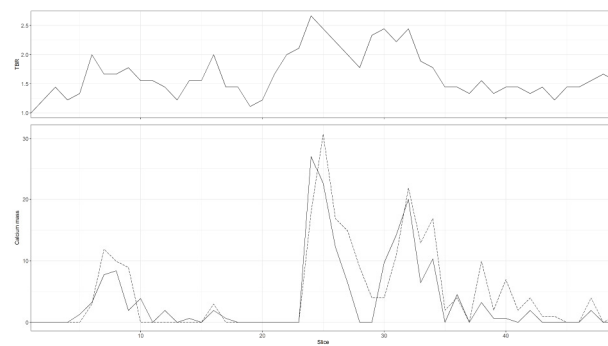

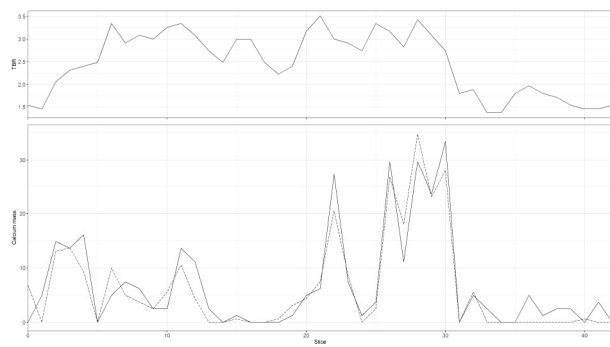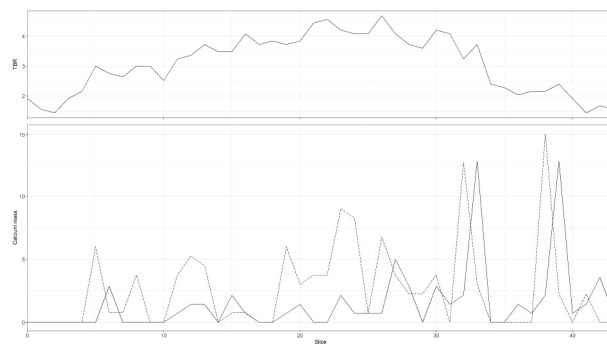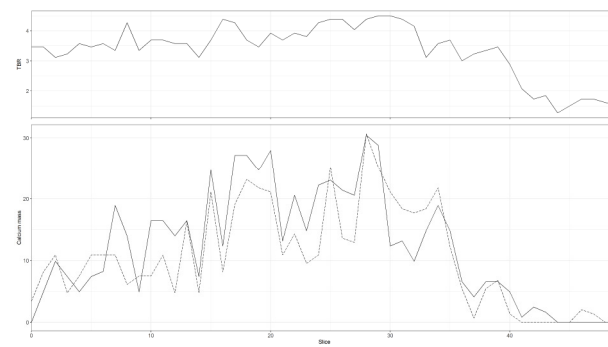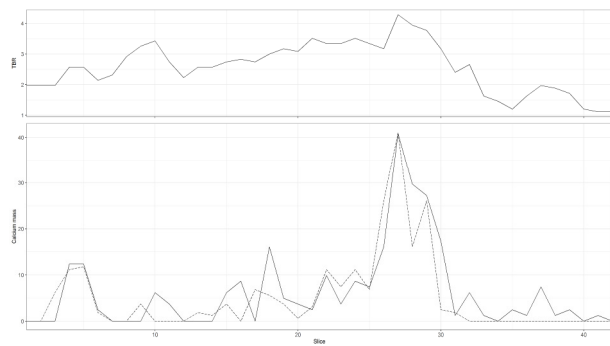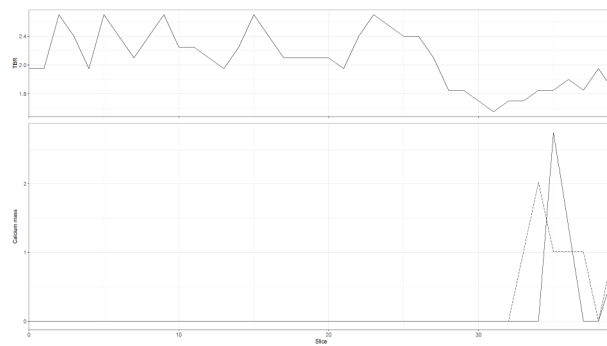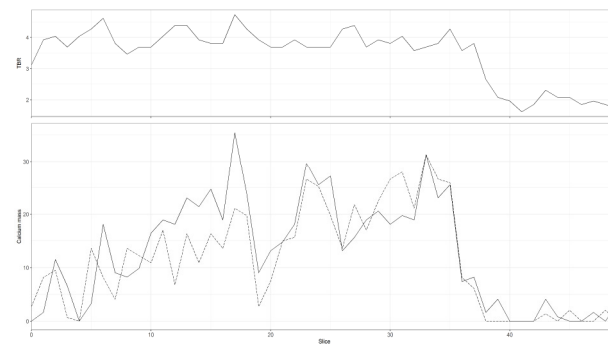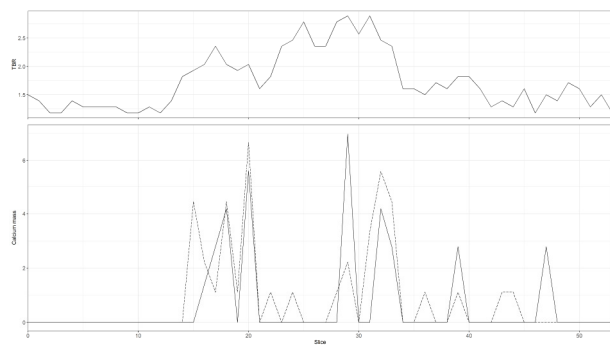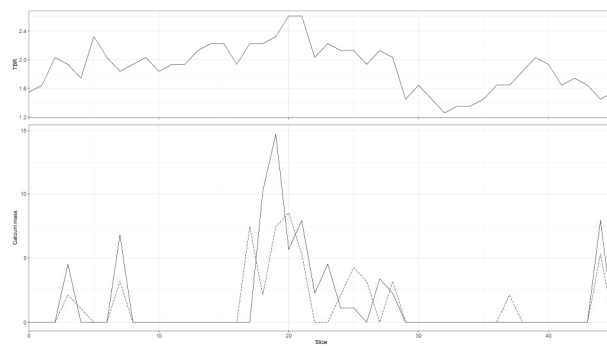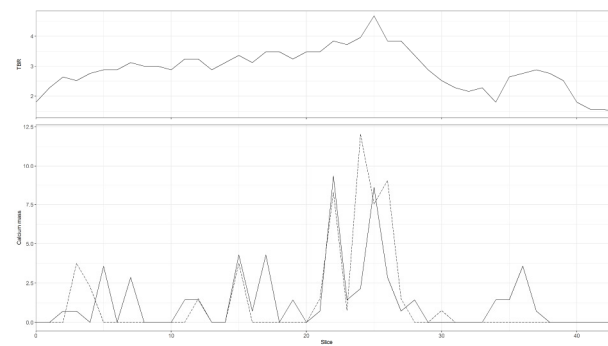

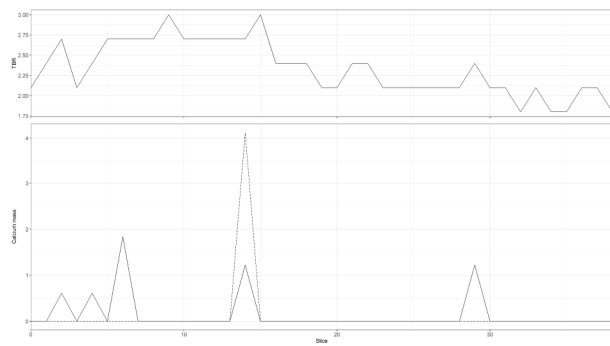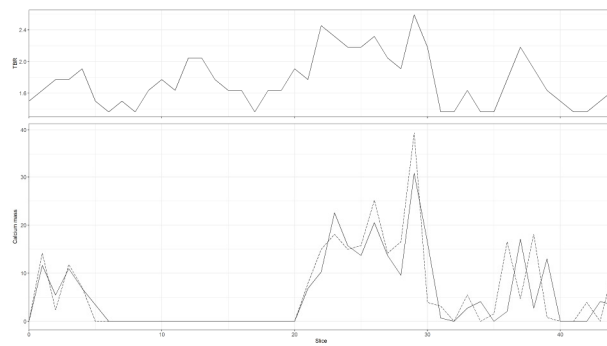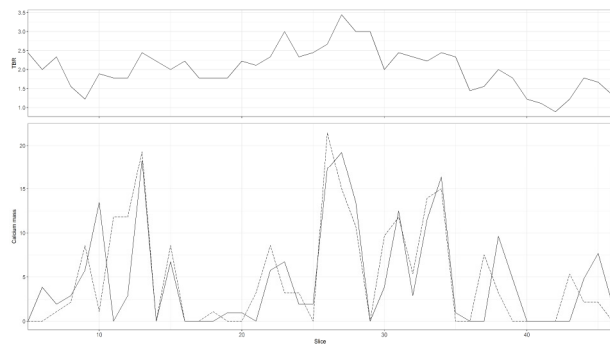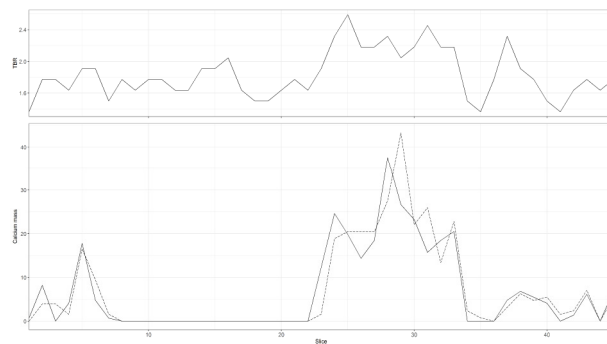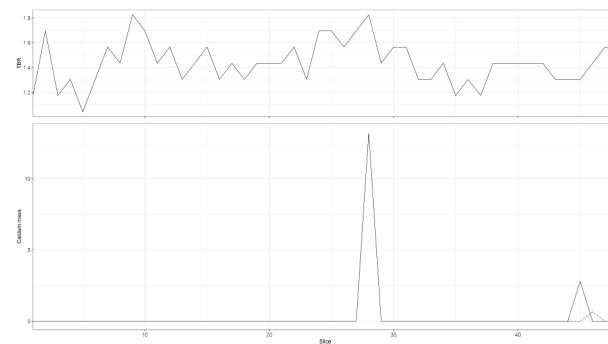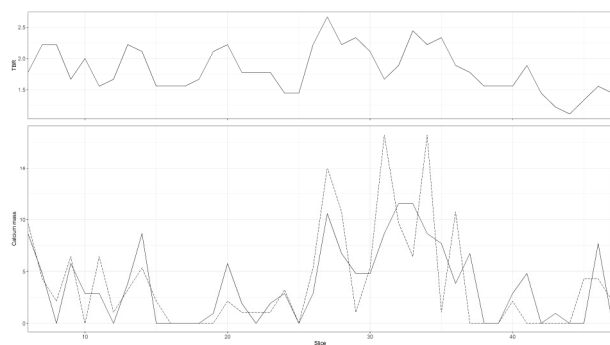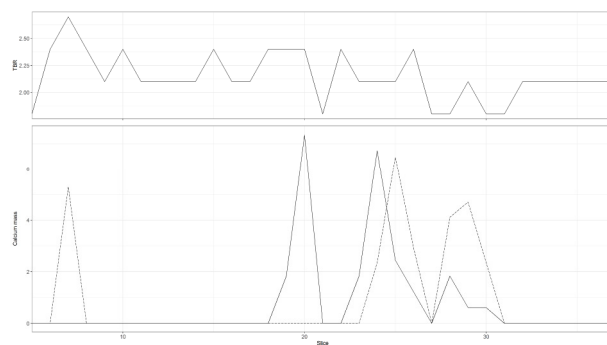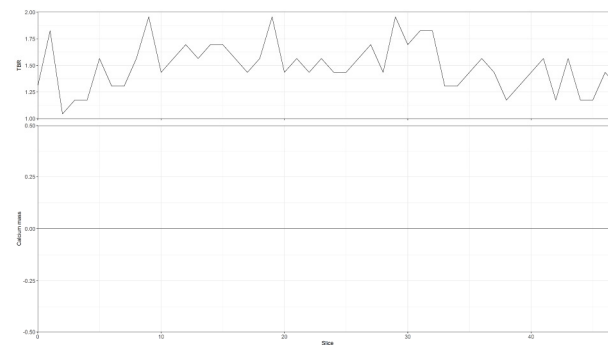

Supplement: Supplementary file 2 — Online Appendix 2: Plots of relationship between TBR at baseline and the calcium mass at baseline and follow-up for the VITACAL study (PDF 5181 kb) [file 12350_2020_2031_MOESM2_ESM.pdf]
