# Supplementary material for: Vascular uptake on 18F-sodium fluoride positron emission tomography: precursor of vascular calcification?
Source: J Nucl Cardiol. 2020 Jan 23;28(5):2244–54. doi: 10.1007/s12350-020-02031-5 (PMC8648691; doi:10.1007/s12350-020-02031-5)
Supplement: Supplementary file 3 — Online Appendix 3: Results of univariate linear regression (DOCX 14 kb) [file 12350_2020_2031_MOESM3_ESM.docx]

*Appendix 3:* Results of univariate linear regression.

| **Variable** | **Regression coefficient β [95% CI]** | **Correlation coefficient** | **p-value** |
| --- | --- | --- | --- |
| *TEMP study* | | | |
| Age (years) | 1.006 [1.004 – 1.007] | 0.161 | P<0.001 |
| Gender (male) | 1.023 [1.004 – 1.043] | Not applicable | 0.020 |
| BMI (kg/m2) | 0.993 [0.990 – 0.995] | -0.058 | P<0.001 |
| Creatinine level (µmol/l) | 1.001 [1.001 – 1.002] | 0.035 | P<0.001 |
| Baseline calcium mass | 1.006 [1.005 – 1.006] | 0.476 | P<0.001 |
| Delta calcium mass | 0.998 [0.997 – 0.999] | -0.070 | P<0.001 |
|  |  |  |  |
| *VITACAL study* | | | |
| Age (years) | 1.005 [1.003 – 1.006] | 0.079 | P<0.001 |
| Gender (male) | 0.981 [0.954 – 1.008] | Not applicable | 0.166 |
| BMI (kg/m2) | 1.010 [1.008 – 1.012] | 0.145 |  |
| Creatinine level (µmol/l) | 1.000 [1.000 – 1.001] | -0.039 | P<0.001 |
| Baseline calcium mass | 1.014 [1.013 – 1.014] | 0.493 | P<0.001 |
| Delta calcium mass | 1.004 [1.002 – 1.006] | 0.054 | P<0.001 |
