# Supplementary material for: Vascular uptake on 18F-sodium fluoride positron emission tomography: precursor of vascular calcification?
Source: J Nucl Cardiol. 2020 Jan 23;28(5):2244–54. doi: 10.1007/s12350-020-02031-5 (PMC8648691; doi:10.1007/s12350-020-02031-5)
Supplement: Supplementary file 4 — Online Appendix 4: Results of multilevel linear regression models stratified per treatment group for both studies (DOCX 20 kb) [file 12350_2020_2031_MOESM4_ESM.docx]

*Appendix 4:* Results of multilevel linear regression models stratified per treatment group for both studies.

| **Variable** | **Regression coefficient β [95% CI]** | **p-value** |
| --- | --- | --- |
| *TEMP study – placebo group* | | |
| Age (years) | 1.00\46 [0.993 – 1.016] | 0.481 |
| Gender (male) | 1.048 [0.843 – 1.302] | 0.391 |
| BMI (kg/m2) | 0.989 [0.961 – 1.018] | 0.463 |
| Creatinine level (µmol/l) | 0.996 [0.987 – 1.005] | 0.442 |
| Baseline calcium mass | 1.006 [1.006 – 1.007] | P<0.001 |
| Delta calcium mass | 1.003 [1.002 – 1.004] | P<0.001 |
|  |  |  |
| *TEMP study – etidronate group* | | |
| Age (years) | 0.999 [0.986 – 1.012] | 0.899 |
| Gender (male) | 1.019 [0.806 – 1.287] | 0.883 |
| BMI (kg/m2) | 0.985 [0.962 – 1.009] | 0.244 |
| Creatinine level (µmol/l) | 0.999 [0.991 – 1.007] | 0.841 |
| Baseline calcium mass | 1.005 [1.005 – 1.005] | P<0.001 |
| Delta calcium mass | 1.002 [1.001 – 1.002] | P<0.001 |
|  |  |  |
| *VITACAL study – placebo group* | | |
| Age (years) | 1.002 [0.989 – 1.015] | 0.792 |
| Gender (male) | 0.984 [0.744 – 1.301] | 0.914 |
| BMI (kg/m2) | 1.011 [0.989 – 1.034] | 0.362 |
| Creatinine level (µmol/l) | 0.999 [0.995 – 1.003] | 0.533 |
| Baseline calcium mass | 1.012 [1.011 – 1.013] | P<0.001 |
| Delta calcium mass | 1.005 [1.004 – 1.007] | P<0.001 |
|  |  |  |
| *VITACAL study – vitamin K group* | | |
| Age (years) | 1.004 [0.992 -1.016] | 0.524 |
| Gender (male) | 1.020 [0.810– 1.285] | 0.876 |
| BMI (kg/m2) | 1.010 [0.995 – 1.025] | 0.210 |
| Creatinine level (µmol/l) | 0.999 [0.995 – 1.003] | 0.659 |
| Baseline calcium mass | 1.015 [1.014 -1.016] | P<0.001 |
| Delta calcium mass | 1.007 [1.005 – 1.008] | P<0.001 |

.
