# Supplementary material for: Vascular uptake on 18F-sodium fluoride positron emission tomography: precursor of vascular calcification?
Source: J Nucl Cardiol. 2020 Jan 23;28(5):2244–54. doi: 10.1007/s12350-020-02031-5 (PMC8648691; doi:10.1007/s12350-020-02031-5)
Supplement: Supplementary file 6 — Supplementary material 6 (PPTX 284 kb) [file 12350_2020_2031_MOESM6_ESM.pptx]

## Slide 1
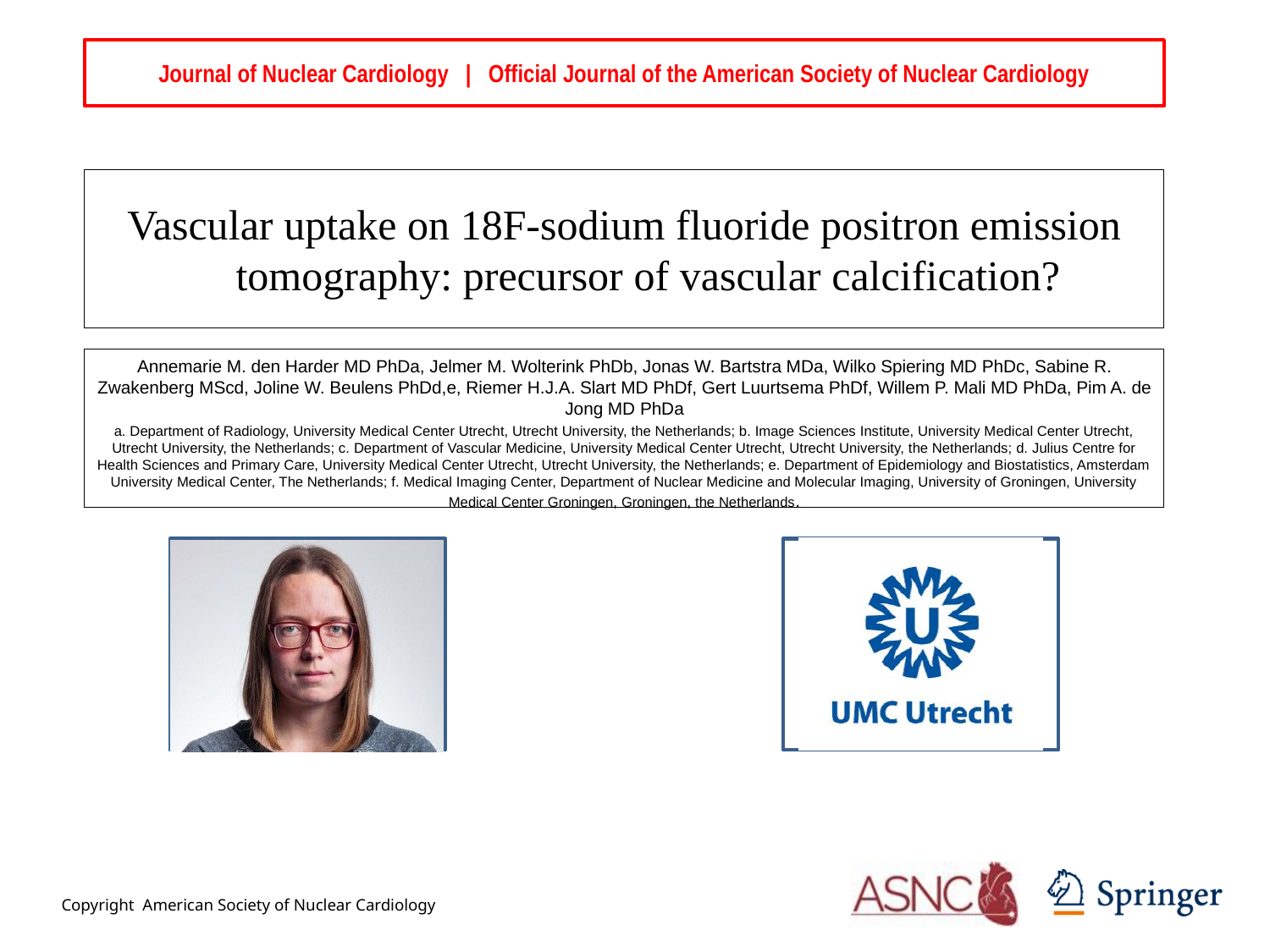

Journal of Nuclear Cardiology | Official Journal of the American Society of Nuclear Cardiology
# Vascular uptake on 18F-sodium fluoride positron emission tomography: precursor of vascular calcification?
Annemarie M. den Harder MD PhDa, Jelmer M. Wolterink PhDb, Jonas W. Bartstra MDa, Wilko Spiering MD PhDc, Sabine R. Zwakenberg MScd, Joline W. Beulens PhDd,e, Riemer H.J.A. Slart MD PhDf, Gert Luurtsema PhDf, Willem P. Mali MD PhDa, Pim A. de Jong MD PhDa
a. Department of Radiology, University Medical Center Utrecht, Utrecht University, the Netherlands; b. Image Sciences Institute, University Medical Center Utrecht, Utrecht University, the Netherlands; c. Department of Vascular Medicine, University Medical Center Utrecht, Utrecht University, the Netherlands; d. Julius Centre for Health Sciences and Primary Care, University Medical Center Utrecht, Utrecht University, the Netherlands; e. Department of Epidemiology and Biostatistics, Amsterdam University Medical Center, The Netherlands; f. Medical Imaging Center, Department of Nuclear Medicine and Molecular Imaging, University of Groningen, University Medical Center Groningen, Groningen, the Netherlands.
Copyright American Society of Nuclear Cardiology

## Slide 2
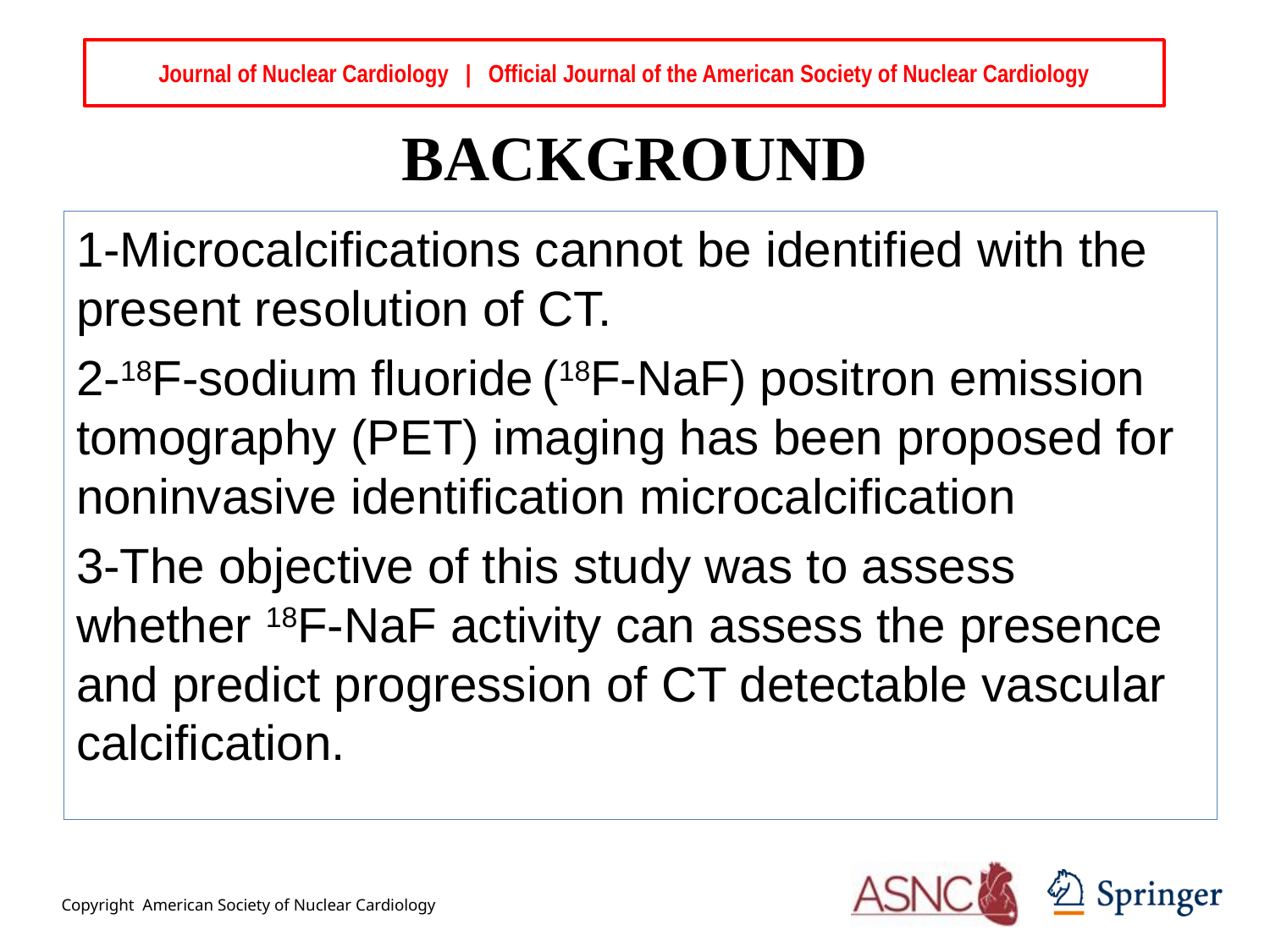

Journal of Nuclear Cardiology | Official Journal of the American Society of Nuclear Cardiology
# BACKGROUND
1-Microcalcifications cannot be identified with the present resolution of CT.
2-18F-sodium fluoride (18F-NaF) positron emission tomography (PET) imaging has been proposed for noninvasive identification microcalcification
3-The objective of this study was to assess whether 18F-NaF activity can assess the presence and predict progression of CT detectable vascular calcification.
Copyright American Society of Nuclear Cardiology

## Slide 3
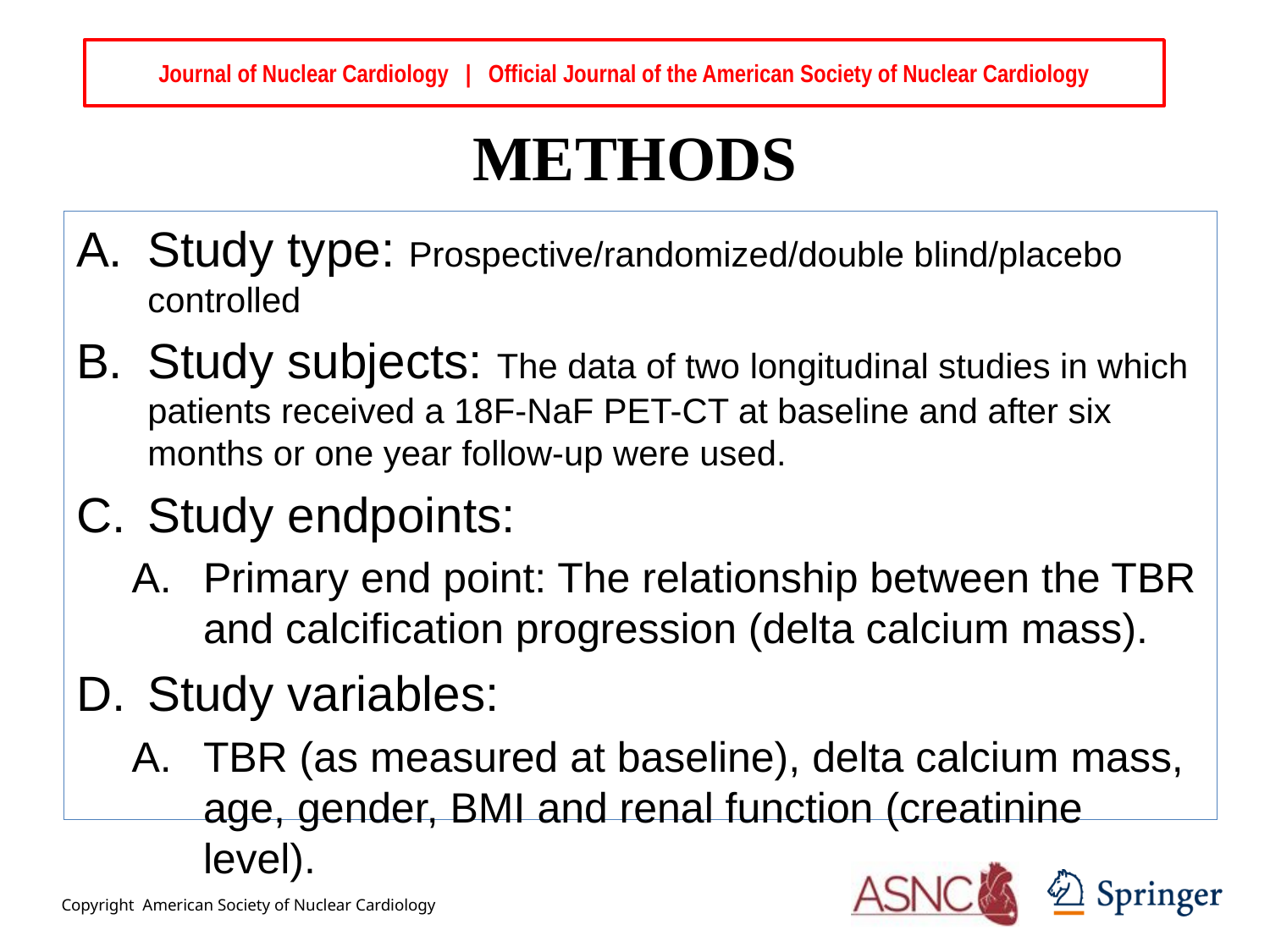

Journal of Nuclear Cardiology | Official Journal of the American Society of Nuclear Cardiology
# METHODS
Study type: Prospective/randomized/double blind/placebo controlled
Study subjects: The data of two longitudinal studies in which patients received a 18F-NaF PET-CT at baseline and after six months or one year follow-up were used.
Study endpoints:
Primary end point: The relationship between the TBR and calcification progression (delta calcium mass).
Study variables:
TBR (as measured at baseline), delta calcium mass, age, gender, BMI and renal function (creatinine level).
Copyright American Society of Nuclear Cardiology

## Slide 4
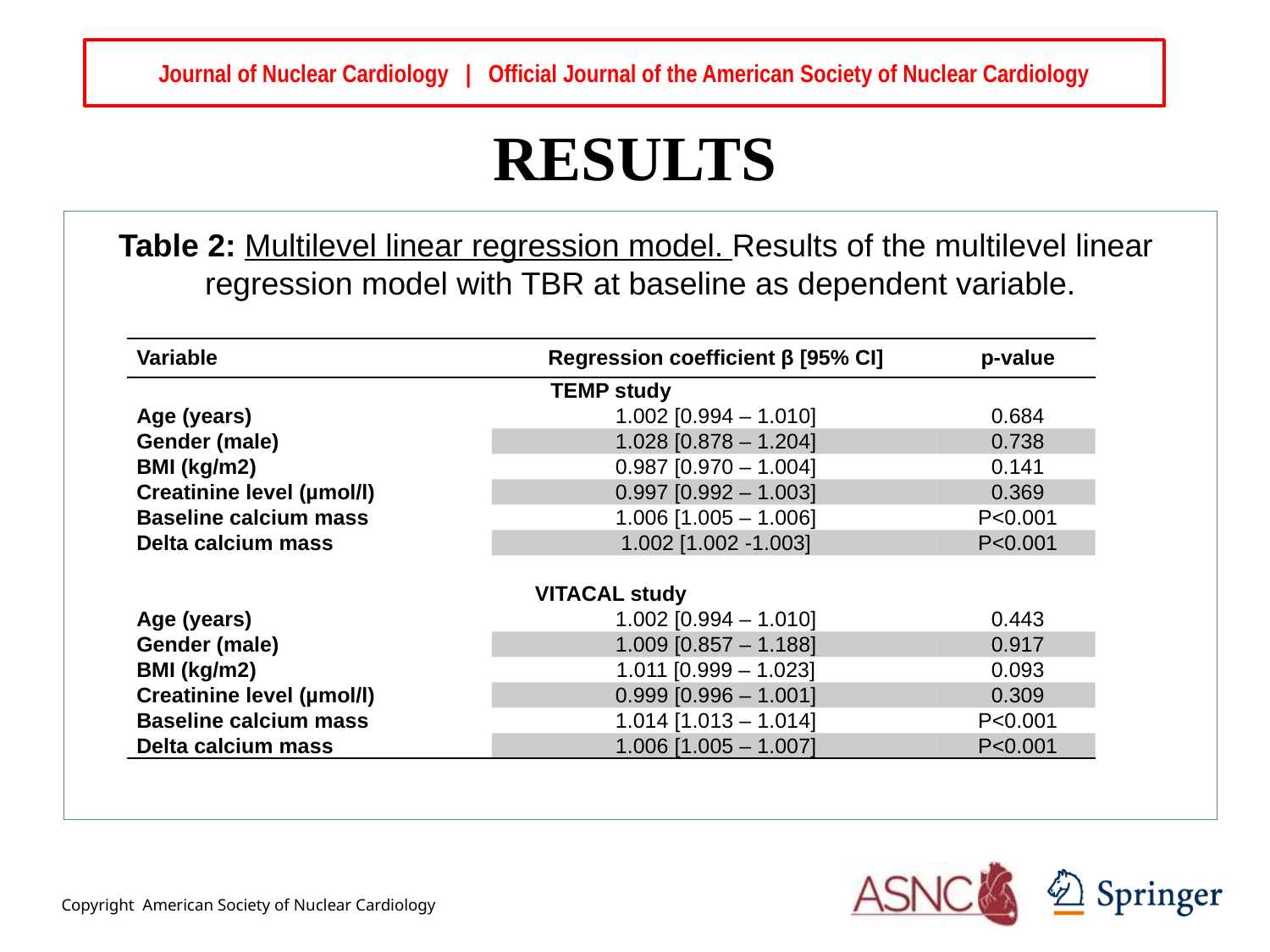

Journal of Nuclear Cardiology | Official Journal of the American Society of Nuclear Cardiology
# RESULTS
Table 2: Multilevel linear regression model. Results of the multilevel linear
regression model with TBR at baseline as dependent variable.
| Variable | Regression coefficient β [95% CI] | p-value |
| --- | --- | --- |
| TEMP study | | |
| Age (years) | 1.002 [0.994 – 1.010] | 0.684 |
| Gender (male) | 1.028 [0.878 – 1.204] | 0.738 |
| BMI (kg/m2) | 0.987 [0.970 – 1.004] | 0.141 |
| Creatinine level (µmol/l) | 0.997 [0.992 – 1.003] | 0.369 |
| Baseline calcium mass | 1.006 [1.005 – 1.006] | P<0.001 |
| Delta calcium mass | 1.002 [1.002 -1.003] | P<0.001 |
| | | |
| VITACAL study | | |
| Age (years) | 1.002 [0.994 – 1.010] | 0.443 |
| Gender (male) | 1.009 [0.857 – 1.188] | 0.917 |
| BMI (kg/m2) | 1.011 [0.999 – 1.023] | 0.093 |
| Creatinine level (µmol/l) | 0.999 [0.996 – 1.001] | 0.309 |
| Baseline calcium mass | 1.014 [1.013 – 1.014] | P<0.001 |
| Delta calcium mass | 1.006 [1.005 – 1.007] | P<0.001 |
Copyright American Society of Nuclear Cardiology

## Slide 5
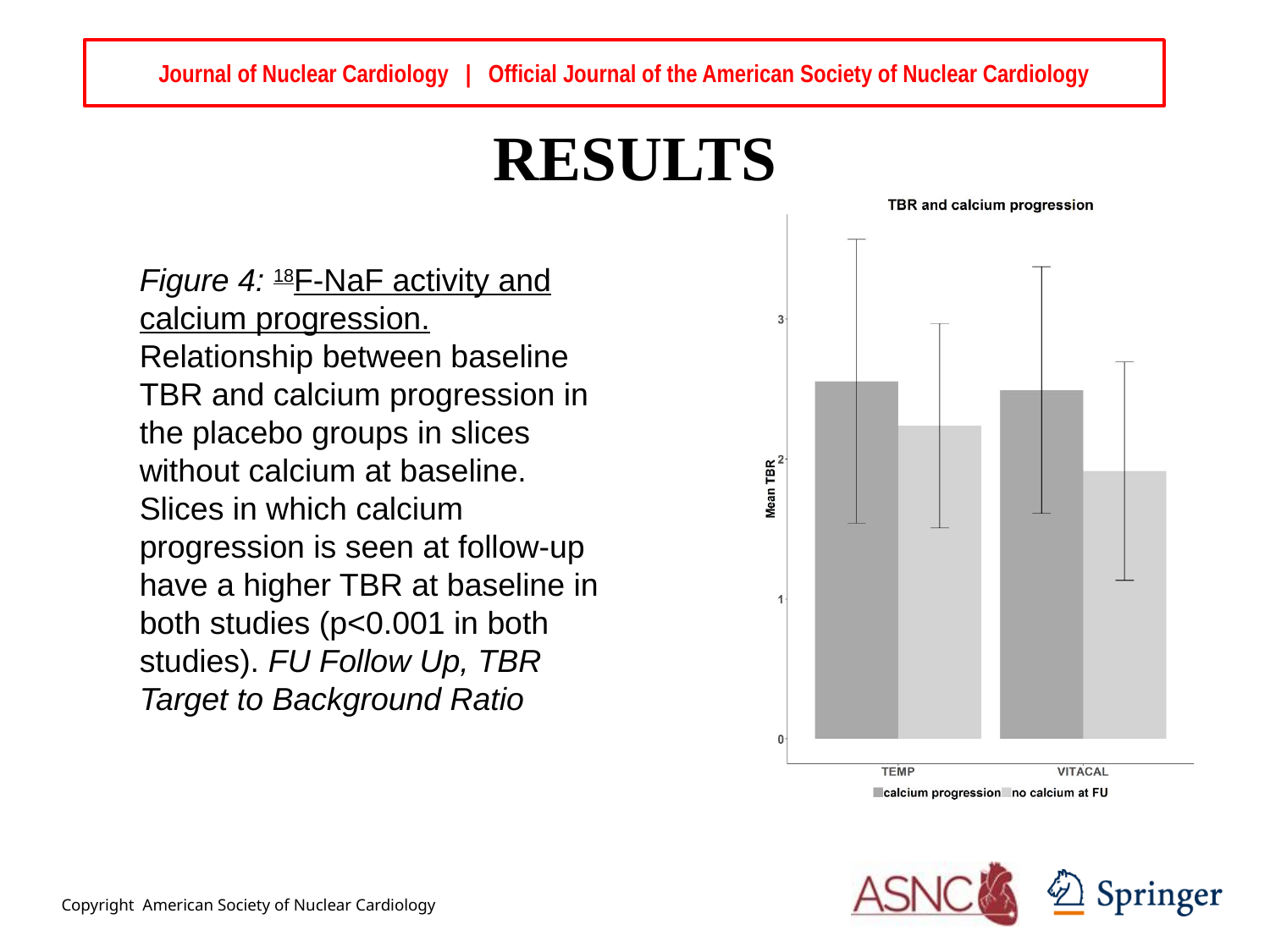

Journal of Nuclear Cardiology | Official Journal of the American Society of Nuclear Cardiology
# RESULTS
Figure 4: 18F-NaF activity and calcium progression. Relationship between baseline TBR and calcium progression in the placebo groups in slices without calcium at baseline. Slices in which calcium progression is seen at follow-up have a higher TBR at baseline in both studies (p<0.001 in both studies). FU Follow Up, TBR Target to Background Ratio
Copyright American Society of Nuclear Cardiology

## Slide 6
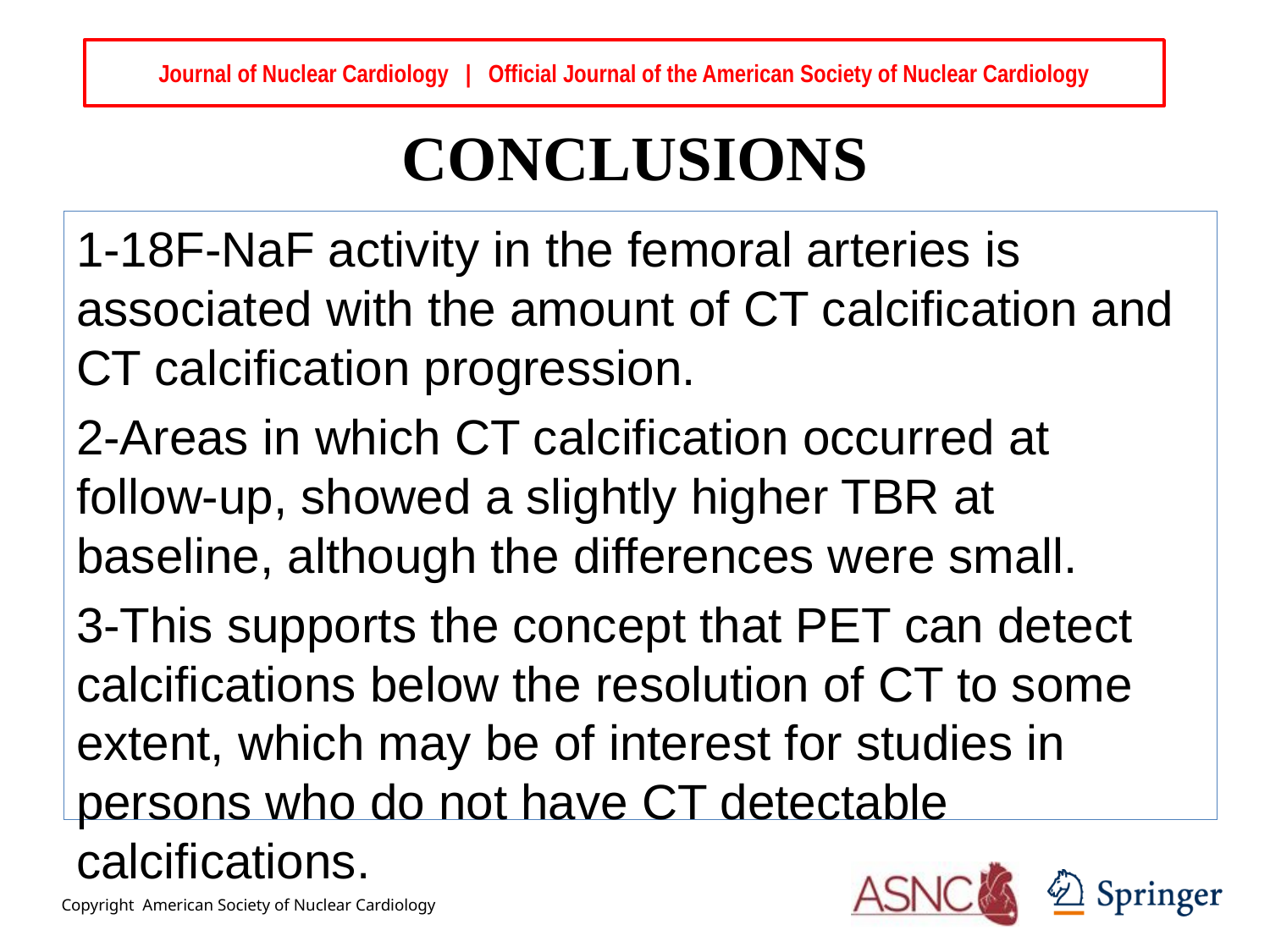

Journal of Nuclear Cardiology | Official Journal of the American Society of Nuclear Cardiology
# CONCLUSIONS
1-18F-NaF activity in the femoral arteries is associated with the amount of CT calcification and CT calcification progression.
2-Areas in which CT calcification occurred at follow-up, showed a slightly higher TBR at baseline, although the differences were small.
3-This supports the concept that PET can detect calcifications below the resolution of CT to some extent, which may be of interest for studies in persons who do not have CT detectable calcifications.
Copyright American Society of Nuclear Cardiology
